# Magnetoelectric nanoparticles drive TAF9B^+^ T_H_2 cell expansion to alleviate inflammation

**DOI:** 10.1126/sciadv.adz3199

**Published:** 2026-02-11

**Authors:** Jia Song, Lulu Liu, Ziqi Liu, Shuo Liu, Zhang Zhang, Xinyu Liu, Boon Chin Heng, Yaojin Wang, Dan Lu, Xuehui Zhang, Xuliang Deng

**Affiliations:** ^1^ Department of Dental Materials & Dental Medical Devices Testing Center, Peking University School and Hospital of Stomatology, Beijing 100081, PR China.; ^2^ Department of Geriatric Dentistry, Peking University School and Hospital of Stomatology, Beijing 100081, PR China.; ^3^ National Center for Stomatology, National Clinical Research Center for Oral Diseases, National Engineering Research Center of Oral Biomaterials and Digital Medical Devices, NMPA Key Laboratory for Dental Materials, Beijing Laboratory of Biomedical Materials & Beijing Key Laboratory of Digital Stomatology, Peking University School and Hospital of Stomatology, Beijing 100081, PR China.; ^4^ First Clinical Division, Peking University School and Hospital of Stomatology, Beijing 100081, PR China.; ^5^ School of Materials Science and Engineering, Nanjing University of Science and Technology, Jiangsu, Nanjing 210094, PR China.; ^6^ Central Laboratory, Peking University School and Hospital of Stomatology, Beijing 100081, PR China.; ^7^ Institute of Systems Biomedicine, School of Basic Medical Sciences, NHC Key Laboratory of Medical Immunology, Beijing Key Laboratory of Tumor Systems Biology, Peking University Health Science Center, Beijing 100191, PR China.; ^8^ Oral Translational Medicine Research Center, Joint Training base for Shanxi Provincial Key Laboratory in Oral and Maxillofacial Repair, Reconstruction and Regeneration, The First People’s Hospital of Jinzhong, Shanxi Province, Jinzhong 030600, PR China.

## Abstract

Stimuli-responsive nanomaterials represent a promising platform for immunomodulation. However, their application in orchestrating T cell responses remains limited. Here, we develop a biomimetic magnetoelectric nanoparticle (DC@CFO/BFO) by coating core-shell CoFe_2_O_4_@BiFeO_3_ particles with dendritic cell membranes to enable selective targeting of CD4^+^ T cells. Under magnetic field stimulation, DC@CFO/BFO localizes to ribosomes and enhances protein synthesis by modulating electrostatic interactions at the ribosomal exit tunnel. This ribosome-targeted modulation promotes type II immune response via IL-4 induction and TAF9B-dependent transcriptional programming, thereby enhancing T helper 2 (T_H_2) cell proliferation. In murine models of colitis and arthritis, both systemic administration of DC@CFO/BFO and adoptive transfer of magnetoelectricity-responsive T_H_2 cells attenuated inflammation and restored immune homeostasis. In contrast, these effects were abrogated in *Taf9b*-deficient T cells, underscoring the essential role of TAF9B in mediating this response. Collectively, our findings identify magnetoelectric nanocomposites as a potent tool for T cell engineering and highlight a translational strategy for the treatment of autoimmune inflammation.

## Teaser

Engineered membrane-coated magnetoelectric nanoparticles drive T_H_2 cell expansion to alleviate autoimmune inflammation.

## INTRODUCTION

CD4^+^ T helper (T_H_) cells orchestrate adaptive immune responses by differentiating into multiple subsets with distinct effector functions (*1*–*3*). Beyond the initial discovery of T_H_1 and T_H_2 cells, additional subsets have been identified, including T_H_17, T_H_9, T_H_22, T_H_25, T follicular helper (Tfh) cells, and regulatory T cells (T_reg_ cells). Many of these lineages, particularly T_H_17 and T_reg_ cell, exhibit plasticity, allowing functional repolarization in response to changing immune contexts, thus shifting the paradigm from rigid lineage stability to dynamic adaptability (*4*–*6*). Within this broader network, however, T_H_1 and T_H_2 remain among the most stable and reciprocally inhibitory subsets. T_H_1 cells, characterized by interferon-γ (IFN-γ) secretion, mediate pro-inflammatory responses and contribute to autoimmune pathogenesis such as type 1 diabetes, multiple sclerosis, and rheumatoid arthritis. By contrast, T_H_2 cells, via cytokines such as interleukin-4 (IL-4) and IL-13, dampen macrophage activation and promote tissue repair (*7*–*9*). Accordingly, restoring the T_H_1/T_H_2 balance continues to represent a robust therapeutic strategy in autoimmunity.

Recent efforts have focused on leveraging physical stimuli to locally modulate T cell fate, offering spatiotemporal precision and reduced systemic toxicity compared to conventional immunotherapies. External electric or magnetic fields have been shown to influence immune cell behavior, including T cell migration and cytokine production (*10*–*14*). These findings suggest that engineered materials capable of delivering controlled biophysical cues could direct T cell polarization without the need for exogenous cytokines.

Nanoparticle design plays a critical role in determining biological interactions and therapeutic outcomes. Coating nanoparticles with natural cell membranes, such as those from antigen-presenting cells, can confer immune evasion, tissue targeting, and enhanced functionality (*15*–*19*). When combined with stimuli-responsive properties, such as magnetoelectricity, these hybrid materials enable precise, noninvasive modulation of immune responses (*20*–*24*).

Protein synthesis in T cells is tightly linked to energy metabolism and functional state (*25*, *26*). Recent evidence suggests that local electrostatic environments, particularly within the ribosomal exit tunnel, can influence translation dynamics (*27*–*30*). However, whether electroactive materials can modulate ribosome function to reprogram T cell responses remains unknown.

**Fig. 1.**
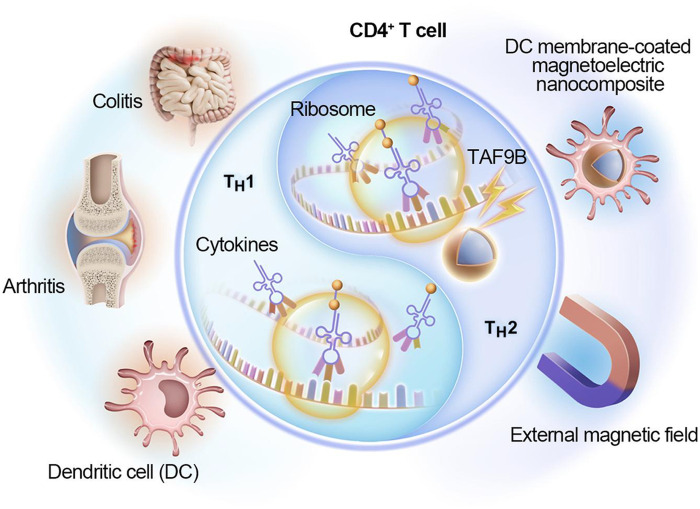
Schematic diagram of the DC membrane–camouflaged magnetoelectric nanocomposites in CD4^+^ T cell regulation. With external magnetic field loading, DC@CFO/BFO nanocomposites promoted T_H_2 cell proliferation and ameliorated T cell mediated autoimmune response, thereby maintaining host immune homeostasis.

Here, we report the development of a biomimetic magnetoelectric nanocomposite (DC@CFO/BFO), composed of a CoFe_2_O_4_@BiFeO_3_ core to shell nanoparticle coated with dendritic cell (DC) membrane, to remotely control CD4^+^ T cell fate. Upon magnetic stimulation, the magnetoelectric core generates localized electric fields that enhance ribosomal activity, promoting IL-4 production and activation of the T_H_2-associated transcription factor TAF9B. This drives robust T_H_2 polarization in vitro. In murine models of colitis and arthritis, treatment with stimulated DC@CFO/BFO nanocomposites induces T_H_2 deviation and alleviates inflammatory pathology, highlighting a strategy for immunomodulation via magnetoelectric nanostimulation (Fig. 1).

## RESULTS

### Fabrication and characterization of the DC membrane–coated magnetoelectric nanoparticles

**Fig. 2.**
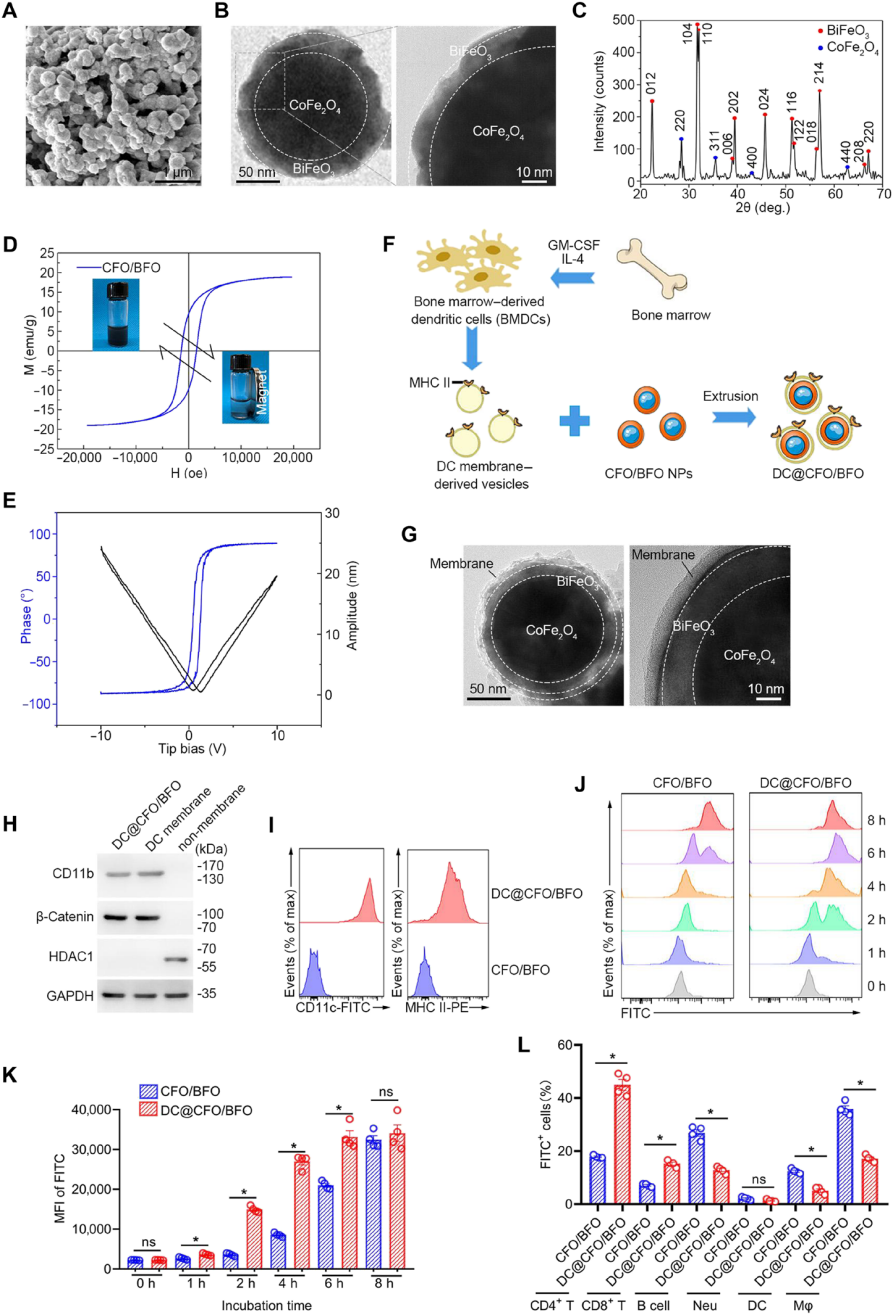
Fabrication and characterization of DC@CFO/BFO nanoparticles. (**A**) Representative SEM images of the CFO/BFO nanoparticles. Scale bar, 1 μm. (**B**) Representative high-resolution TEM images of the CFO/BFO nanoparticles. (**C**) XRD patterns of CFO/BFO nanoparticles. Red, BiFeO_3_ (BFO); blue, CoFe_2_O_4_ (CFO). (**D**) VSM of the CFO/BFO nanocomposites. (**E**) Piezoresponsive amplitude curve and phase curve of CFO/BFO nanocomposites. (**F**) Graphic illustration of the preparation of DC@CFO/BFO nanocomposites. Briefly, bone marrow derived from murine femur and tibia was first isolated, and recombinant granulocyte-macrophage colony-stimulating factor (GM-CSF) (20 ng/ml) and recombinant IL-4 (10 ng/ml) were used to induce DCs differentiation. DCs were sorted by flow cytometry. The harvested DCs were then lysed and the resulting vesicles were subsequently extruded serially through 400-nm and then 200-nm polycarbonate porous membranes using an Avanti mini extruder. (**G**) Representative high-resolution TEM images of the DC@CFO/BFO nanoparticles. (**H**) Immunoblot analysis of surface proteins of DC@CFO/BFO nanoparticles. Cell membrane–associated proteins (CD11b and β-catenin) and nuclear protein (HDAC1) were measured. (**I**) Nano-flow cytometry (NanoFCM, CytoFlex) analysis of expression levels of CD11c and MHC II on the surface of DC@CFO/BFO nanoparticles. (**J** and **K**) Fluorescein isothiocyanate (FITC)-conjugated CFO/BFO nanoparticles or DC@CFO/BFO nanocomposites (10 μg/ml) were incubated with murine naïve CD4^+^ T cells for indicated time durations, and the FITC^+^ cells were detected by flow cytometry. MFI, mean fluorescence intensity [*n* = 4, mean ± SEM; ns, not significant (*P* > 0.05) and *P = 0.0286, Mann-Whitney *U* test]. (**L**) FITC-conjugated CFO/BFO nanoparticles or DC@CFO/BFO nanocomposites (10 μg/ml) were incubated with murine splenocytes for 12 hours, and FITC^+^ cells were detected by flow cytometry [*n* = 4, means ± SEM; ns, not significant (*P* > 0.05) and *P = 0.0286, Mann-Whitney *U* test].

Previous studies have demonstrated the utility of magnetoelectric materials in modulating the function of epithelial cells and macrophages (*31*–*33*); however, their direct application to CD4^+^ T cells remains unexplored. Here, we synthesized nano-sized core-shell composites comprising magnetostrictive cobalt (II) ferrite CoFe_2_O_4_ (CFO) cores surrounded by piezoelectric bismuth (III) ferrite BiFeO_3_ (BFO) shells (CFO/BFO) (fig. S1A). Scanning electron microscopy (SEM) and high-resolution transmission electron microscopy (TEM) confirmed the morphology and clear core-shell structure of CFO/BFO nanocomposites (Fig. 2, A and B). By contrast, CFO particles exhibited a uniform spherical shape with visible crystal lattice, with the diameter of ~108.7 nm (fig. S1, B and C). X-ray diffraction (XRD) spectroscopy validated the crystalline composition, confirming the presence of both CoFe_2_O_4_ and BiFeO_3_ (Fig. 2C), while energy-dispersive spectroscopy (EDS) mapping indicated uniform elemental distribution (fig. S1D). Vibrating sample magnetometer (VSM) analysis demonstrated the excellent responsiveness of CFO/BFO nanocomposites to external magnetic fields, evident from their initial magnetization and saturation magnetization (Fig. 2D). Piezoresponse force microscopy (PFM) revealed detectable local piezoelectric response hysteresis loops at random positions of nanoparticles, characterizing their ferroelectricity and magnetoelectricity (Fig. 2E). In addition, electron paramagnetic resonance (EPR) spectroscopy using 5,5-dimethyl-1-pyrroline *N*-oxide (DMPO) detected oxide species (•OH and •O_2_^−^) generated in magnetoelectrocatalysis, with signals observed in CFO/BFO nanocomposites under an external magnetic field (fig. S2, A and B). Moreover, because of the piezoelectric properties of BFO, magnetoelectric CFO/BFO nanocomposites and BFO nanoparticles could produce the characteristic signals of •OH and •O_2_^−^ in the presence of exogenous ultrasound, whereas nanoparticles alone were unable to trigger reactive oxygen species (ROS) production (fig. S2, C to F). In addition, we used finite element analysis (FEA) simulations to further characterize the change in surface charge of the magnetoelectric composites under magnetic field treatment. As shown in fig. S3A, CFO/BFO nanoparticles became more negatively charged under magnetic field treatment. These findings thus elucidated the role of magnetic fields in inducing magnetostrictive strain of the CFO core and initiating the BFO shell’s potential polarization, thus confirming efficient magnetoelectric transduction.

To confer CD4^+^ T cell specificity, we coated nanoparticles with membranes from murine bone marrow–derived dendritic cells (BMDCs), known to interact specifically with CD4^+^ T cells via major histocompatibility complex class II (MHC II)–T cell receptor (TCR) engagement. Isolated BMDCs membranes were extruded with CFO/BFO nanoparticles to yield DC@CFO/BFO composites displaying intact membrane structures (~10-nm thick; Fig. 2, F and G, and fig. S4, A to E), and the nanoparticles exhibited excellent dispersibility (fig. S4F). As shown in fig. S3B, DC@CFO/BFO nanoparticles exhibited decreased surface charge without magnetic field treatment compared with CFO/BFO nanoparticles. However, no significant difference was observed between DC@CFO/BFO and CFO/BFO under magnetic field treatment. Western blotting, nano-flow cytometry, glycoprotein quantification, and proteomics analysis collectively confirmed successful membrane protein retention, including key MHC II components (Fig. 2, H and I, and fig. S4, G to J).

Cytotoxicity assays demonstrated excellent biocompatibility of DC@CFO/BFO nanoparticles below 100 μg/ml (fig. S5, A to C), guiding our selection of 10 μg/ml for further studies. Flow cytometry revealed enhanced nanoparticle uptake by CD4^+^ T cells facilitated by DC membrane coating, peaking within 8 hours (Fig. 2, J and K). To distinguish between surface binding and true internalization, we compared nanoparticle–T cell interactions at 37°C (binding + internalization) versus 4°C (binding only). As shown in fig. S5 (D to G), flow cytometry revealed that DC@CFO/BFO nanoparticles exhibited markedly increased uptake by naïve CD4^+^ T cells at 37°C, with uptake peaking around 8 hours. In contrast, fluorescence signals were significantly reduced at 4°C, indicating that binding alone could not account for the observed signal. These results suggest that the elevated fluorescence at 37°C primarily reflects active internalization of DC@CFO/BFO nanoparticles, rather than surface attachment. We also used A-286982, a nonpeptide antagonist of the LFA-1/intercellular adhesion molecule–1 (ICAM-1) interaction (*34*), which selectively block DC–T cell binding without activating T cells. As shown in fig. S5 (H and I), pretreatment of naïve CD4^+^ T cells with A-286982 markedly reduced the uptake of DC@CFO/BFO nanoparticles. These findings confirm that the enhanced uptake of DC@CFO/BFO by T cells is mediated, at least in part, by the LFA-1/ICAM-1 axis, thereby demonstrating that the DC membrane coating confers true targeting specificity to the magnetoelectric nanocomposites.

In addition, in murine splenocyte cultures, DC-coated nanoparticles were preferentially internalized by CD4^+^ T cells, whereas bare nanoparticles predominantly accumulated in macrophages (Fig. 2L). In vivo, intravenous administration in C57BL/6 mice confirmed selective targeting of DC@CFO/BFO nanoparticles to T cells rather than macrophages or DCs, underscoring the effectiveness of DC membrane coating for T cell specificity (fig. S5J). Collectively, these data confirm the successful fabrication and characterization of DC membrane–coated magnetoelectric nanoparticles specifically targeting CD4^+^ T cells.

### DC@CFO/BFO nanoparticles enhance protein synthesis and amino acid metabolism by targeting ribosomes in T cells

**Fig. 3.**
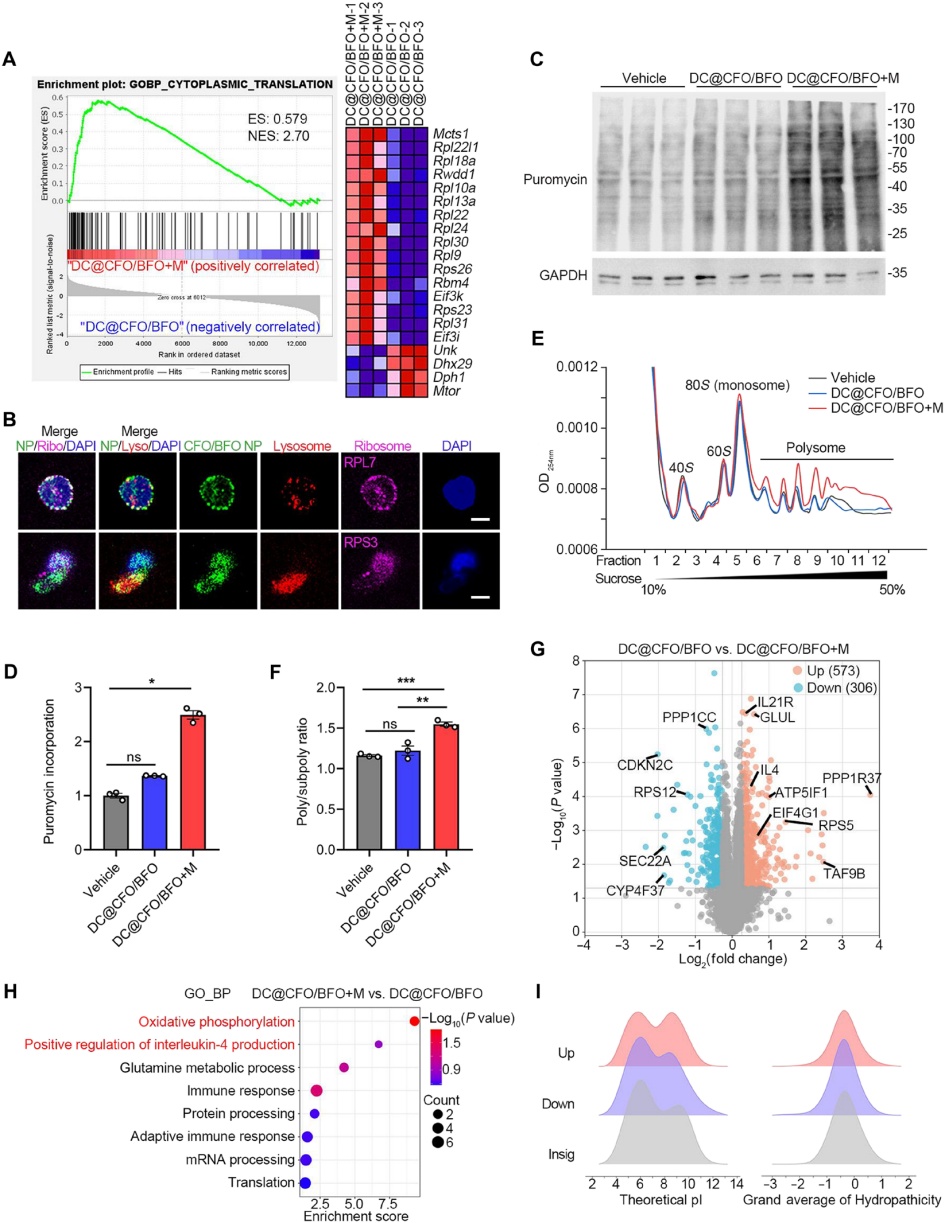
DC@CFO/BFO nanoparticles enhance protein translation in Th2 cells under magnetic field stimulation. (**A**) CD4^+^ T cells were activated with antibodies against CD3/CD28, treated by DC@CFO/BFO nanocomposites and subjected to magnetic field stimulation. Gene set enrichment analysis (GSEA) of genes expressed in CD4^+^ T cells treated by DC@CFO/BFO nanocomposites in the presence or absence of magnetic field stimulation (*n* = 3 biological replicates). ES, enrichment score; NES, normalized enrichment score. (**B**) Imaging of CD4^+^ T cells treated by FITC-conjugated CFO/BFO nanocomposites. RPS3 and RPL7 were used to label the ribosome. LysoTracker Red was used to track lysosome. Scale bar, 5 μm. (**C** and **D**) CD4^+^ T cells were activated with antibodies against CD3/CD28, treated with DC@CFO/BFO nanocomposites and subjected to magnetic field stimulation. Puromycin-labeled proteins in T cells were identified with immunoblot analysis [*n* = 3, means ± SEM; ns (*P* > 0.05) and *P = 0.0219, Kruskal-Wallis Test]. (**E** and **F**) CD4^+^ T cells were activated with antibodies against CD3/CD28, treated with DC@CFO/BFO nanocomposites and subjected to magnetic field stimulation. Polysome profiling analysis of T cells was performed [*n* = 3, means ± SEM; ns, not significant (*P* > 0.05); **P = 0.0020; and ***P = 0.0008 (Shapiro-Wilk test, *P* > 0.1; Brown-Forsythe test, *P* > 0.1) one-way analysis of variance (ANOVA)]. (**G**) Volcano plot analysis of pairwise comparison of quantitative proteomics analysis between the DC@CFO/BFO+M group and DC@CFO/BFO group. (**H** and **I**) CD4^+^ T cells were activated with antibodies against CD3/CD28, treated with DC@CFO/BFO nanocomposites, and subjected to magnetic field stimulation. Proteins that were significantly up-regulated in T cells with DC@CFO/BFO+M treatment compared to T cells with DC@CFO/BFO treatment were analyzed using DAVID with GO terms (H) Theoretical isoelectric points (pI) and hydrophilicity of up-regulated proteins, down-regulated proteins, and insignificant proteins were shown, respectively (I). OD_450nm_, optical density at 450 nm.

To evaluate the impact of DC@CFO/BFO nanoparticles on T cell fate under magnetic stimulation, we activated T cells using anti-CD3/CD28 antibodies in the presence or absence of these nanoparticles. Transcriptome analysis identified notable enrichment of pathways related to “cytoplasmic translation” and “ribosome” following treatment with DC@CFO/BFO nanoparticles combined with magnetic stimulation (Fig. 3A and fig. S6A). Untargeted metabolomics revealed widespread metabolic alterations in T cells induced by DC@CFO/BFO + M treatment (fig. S7A). Specifically, differential metabolite analysis showed that DC@CFO/BFO nanoparticles with magnetic stimulation substantially enhanced cellular metabolism, evidenced by 398 metabolites up-regulated and 61 metabolites down-regulated (fig. S7, B and C). Notably, amino acid metabolic pathways—including “tyrosine metabolism” and “glycine, serine, and threonine metabolism”—emerged as central hubs within the T cell metabolic network (fig. S7, D and E). Representative differential metabolites are shown in fig. S7 (F to J).

Subsequent subcellular localization analysis using confocal microscopy demonstrated that fluorescein isothiocyanate (FITC)–labeled CFO/BFO nanoparticles not only localized in lysosomes but also colocalized with ribosomal proteins large ribosomal subunit protein uL30 (RPL7) and small ribosomal subunit protein uS3 (RPS3) (Fig. 3B). Ribosomes, the essential cellular machinery for protein synthesis, were directly targeted by these nanoparticles. Consistent with this finding, puromycin incorporation assays indicated accelerated protein synthesis rates in T cells treated with DC@CFO/BFO nanoparticles under external magnetic fields (Fig. 3, C and D). Polysome profiling further confirmed increased polysome formation, suggesting enhanced translation efficiency in the DC@CFO/BFO+M group (Fig. 3, E and F).

To clarify mechanisms underlying magnetoelectric nanoparticle–induced translational regulation, we conducted quantitative proteomic analysis of T cells subjected to DC@CFO/BFO nanoparticles and magnetic field stimulation. Principal components analysis (PCA) distinguished the DC@CFO/BFO+M group from control groups (fig. S8A). Heatmap clustering revealed heterogeneous protein expression patterns induced specifically by DC@CFO/BFO+M treatment (fig. S8B). Analysis identified 573 up-regulated and 306 down-regulated proteins in response to nanoparticles and magnetic field treatment (Fig. 3G). Clusters of orthologous groups analysis of the differential proteins is presented in fig. S8C. Gene Ontology (GO) enrichment analysis highlighted activation of pathways such as “oxidative phosphorylation,” “IL-4 production,” and “translation” following DC@CFO/BFO+M treatment (Fig. 3H and fig. S8D). Given the importance of protein physicochemical properties in translation rates, we analyzed the isoelectric points (pI) and hydrophilicity of differentially expressed proteins. Notably, up-regulated proteins exhibited notably higher pI values compared to down-regulated or unchanged proteins (Fig. 3I). Electrostatic interactions at the ribosomal exit tunnel influence nascent peptide release kinetics, and peptides enriched in positively charged residues typically experience slower ejection (*30*, *35*, *36*). Thus, we propose that DC@CFO/BFO nanoparticles, under magnetic field stimulation, directly target ribosomes and modulate electrostatic interactions within the peptide exit tunnel, enhancing protein translation efficiency.

### DC@CFO/BFO nanoparticles promote T_H_2 cell polarization and proliferation under an external magnetic field

**Fig. 4.**
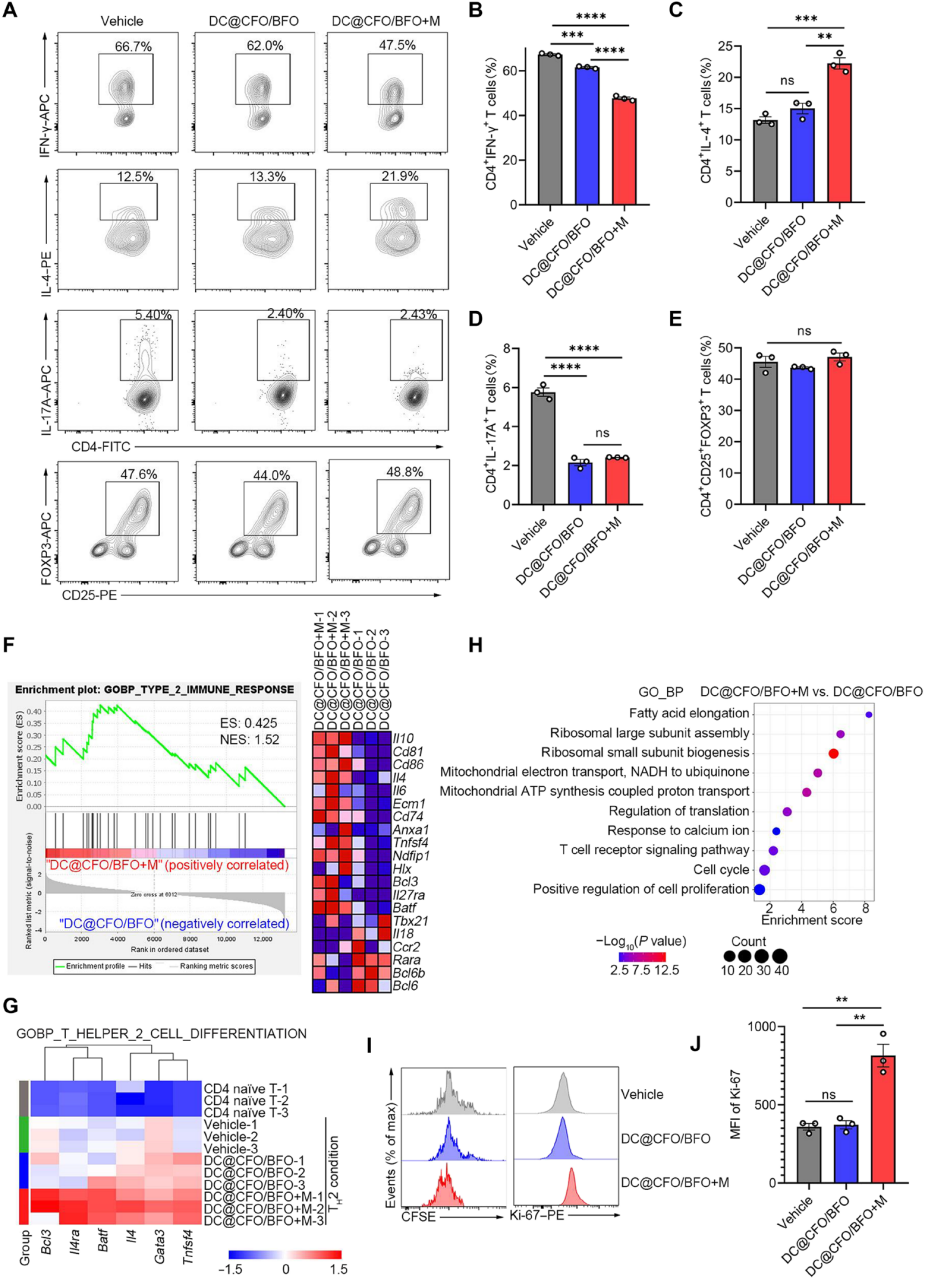
DC@CFO/BFO nanocomposites promote T_H_2 cell polarization and proliferation under external magnetic field stimulation. (**A** to **E**) Flow cytometric analysis of the staining of IFN-γ, IL-4, IL-17A, FOXP3, and CD25 (assessing differentiation efficiency) in naïve T cells (CD4^+^CD25^−^CD62L^hi^CD44^lo^) activated with plate-bound anti-CD3 plus anti-CD28 and cultured under various polarizing condition with or without DC@CFO/BFO nanocomposites and magnetic field treatment and then restimulated for 5 hours with phorbol 12-myristate 13-acetate (PMA) and ionomycin [*n* = 3, means ± SEM, ns, not significant (*P* > 0.05); **P = 0.0014; ***P < 0.001; and ****P < 0.0001 (Shapiro-Wilk test, *P* > 0.1; Brown-Forsythe test, *P* > 0.1) one-way ANOVA]. (**F** to **H**) CD4^+^ T cells were activated with antibodies against CD3/CD28 and were induced to differentiate into T_H_2 cells. These cells were then treated with DC@CFO/BFO nanocomposites in the presence or absence of magnetic field stimulation. GSEA of genes expressed in CD4^+^ T cells under T_H_2-skewing condition treated by DC@CFO/BFO nanocomposites, in the presence or absence of magnetic field (*n* = 3 biological replicates) (F). Heatmap of genes associated with T_H_2 cell differentiation expressed in CD4^+^ T cells in various groups was shown (*n* = 3 biological replicates) (G). Genes that were significantly up-regulated in T cells with DC@CFO/BFO+M treatment, compared with T cells with DC@CFO/BFO treatment, were analyzed using DAVID with GO terms (H). (**I** and **J**) CD4^+^ T cells were activated with antibodies against CD3/CD28 and induced to differentiate into T_H_2 cells. These cells were then treated with DC@CFO/BFO nanocomposites in the presence or absence of magnetic field stimulation. Flow cytometric analysis of CFSE dilution and Ki-67 expression in CD4^+^ T cells was performed [*n* = 3, means ± SEM, ns, not significant (*P* > 0.05) (Shapiro-Wilk test, *P* > 0.1; Brown-Forsythe test, *P* > 0.1) one-way ANOVA].

Given the enhanced IL-4 production induced by DC@CFO/BFO nanoparticles (Fig. 3H), we hypothesized that these nanoparticles could facilitate T_H_2 cell differentiation. To investigate this, naïve CD4^+^ T cells were activated using anti-CD3 and anti-CD28 antibodies under various T_H_ cell–polarizing conditions. Flow cytometric analysis revealed increased IL-4 production and decreased IFN-γ production in T cells treated with DC@CFO/BFO nanoparticles under magnetic stimulation (DC@CFO/BFO+M), indicating enhanced T_H_2 differentiation and suppressed T_H_1 differentiation (Fig. 4, A to C). In addition, IL-17A production was reduced in both DC@CFO/BFO and DC@CFO/BFO+M groups, while induced T_reg_ cell (iT_reg_ cell) proportions remained unchanged across all groups (Fig. 4, A, D and E). To more rigorously assess lineage commitment, we evaluated lineage-defining transcription factors by flow cytometry. As shown in fig. S9, DC@CFO/BFO nanoparticles under magnetic stimulation (DC@CFO/BFO+M) significantly increased the proportion of GATA3^+^CD4^+^ T cells while reducing T-bet^+^CD4^+^ T cells, consistent with enhanced T_H_2 differentiation and suppressed T_H_1 differentiation (fig. S9, A to C). In addition, expression of RORγt was decreased in both DC@CFO/BFO and DC@CFO/BFO+M groups, whereas iT_reg_ cell frequencies (FOXP3^+^) remained unchanged across all groups (fig. S9, A, D and E). In addition, we examined follicular helper T (Tfh) cells and observed no significant changes in Tfh differentiation in vitro (fig. S9, F and G). Luminex assays further validated these findings, showing reduced T_H_1 cytokine levels (IL-2, tumor necrosis factor, and IFN-γ) and elevated T_H_2 cytokine levels (IL-4, IL-5, IL-10, and IL-13) in the DC@CFO/BFO+M treatment group compared with the DC@CFO/BFO group (fig. S9, H to N), thus confirming enhanced T_H_2 polarization. To determine whether the effect of DC@CFO/BFO nanoparticles depends on T_H_2 skewing or is more broadly related to CD4^+^ T cell activation, we measured IL-4 production in naïve CD4^+^ T cells, anti-CD3/CD28–activated T cells, and T_H_2-polarized T cells using the Luminex platform. As shown in fig. S9O, DC@CFO/BFO plus magnetic field treatment significantly enhanced IL-4 production in both activated T cells and T_H_2 cells, with the strongest effect observed under T_H_2-polarizing conditions, but had no impact on naïve T cells. These findings indicate that the activity of magnetoelectric nanocomposites requires T cell activation and is further potentiated by T_H_2-polarizing signals, rather than being strictly dependent on them.

To further confirm this result, we performed RNA sequencing (RNA-seq) on T cells treated with DC@CFO/BFO nanoparticles under T_H_2-polarizing conditions combined with magnetic stimulation. PCA and differential gene expression analysis indicated similar transcriptional profiles for activated T cells treated with or without DC@CFO/BFO nanoparticles in the absence of magnetic stimulation (fig. S10, A and B). However, cells from the DC@CFO/BFO+M group exhibited distinct transcriptional profiles compared to control groups (fig. S10, A and B). GO enrichment analysis also highlighted notable up-regulation of genes involved in “DNA replication,” “T cell differentiation,” and “T cell proliferation” in activated T cells compared to naïve T cells, indicative of robust cellular activation and proliferation under T_H_2-polarizing conditions (fig. S10, C and D).

Subsequently, we profiled the overall gene expression change of T cells treated by DC@CFO/BFO in the presence or absence of magnetic field and found 898 up-regulated genes and 1703 down-regulated genes in T cells upon DC@CFO/BFO+M treatment (fig. S10E). GO enrichment analysis revealed notable up-regulation of genes associated with “type II immune response” and “T_H_2 cell differentiation” (Fig. 4, F and G). In addition, DC@CFO/BFO nanocomposites promoted signaling pathways associated with T cell proliferation upon magnetic field loading (Fig. 4H). Ensued flow cytometry analyses using carboxyfluorescein diacetate succinimidyl ester (CFSE) dilution and Ki-67 staining assays subsequently confirmed increased proliferation of CD4^+^ T cells treated with DC@CFO/BFO+M under T_H_2-skewing conditions (Fig. 4, I and J). Collectively, these results demonstrate that magnetoelectric nanoparticle treatment combined with magnetic field stimulation promotes T_H_2 polarization and enhances T cell proliferative capacity in vitro.

### Magnetoelectricity-responsive T_H_2 cells exhibit anti-inflammatory effects

**Fig. 5.**
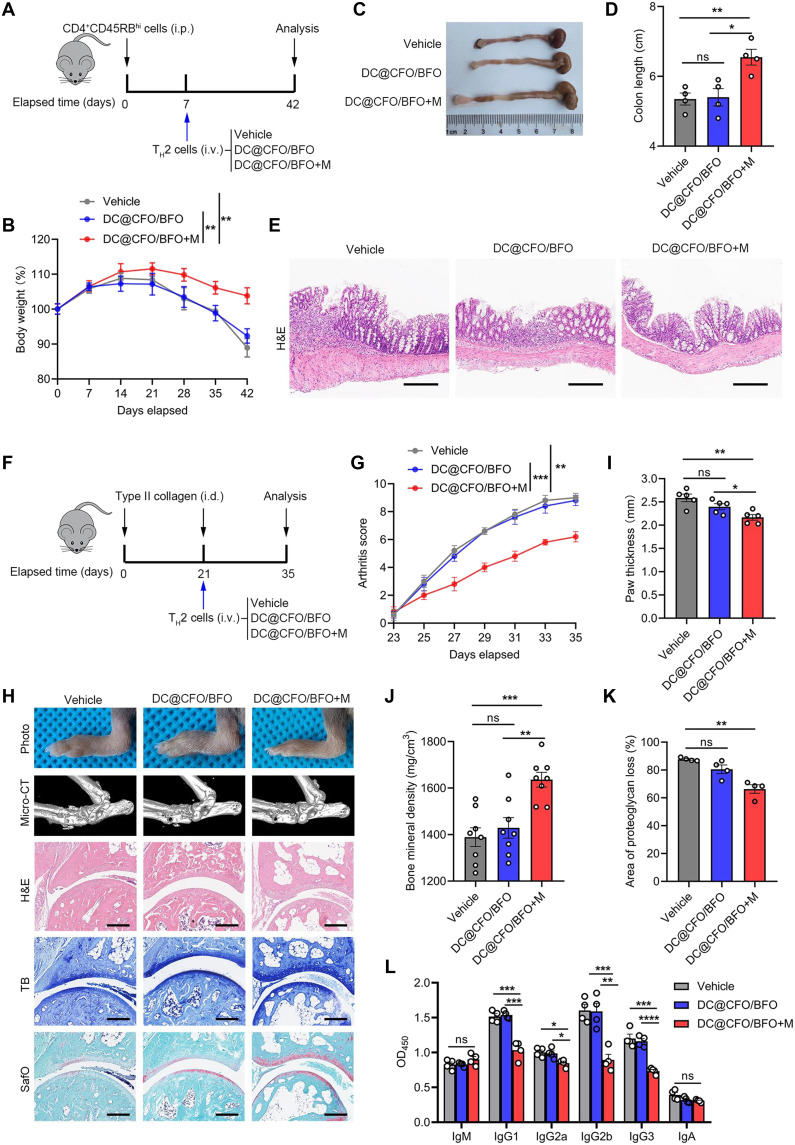
Magnetoelectric nanoparticle–treated T_H_2 cells inhibit inflammatory response. (**A**) Schematic illustration of the study design of DC@CFO/BFO nanoparticle-pretreated T_H_2 cell administration in T cell transfer–induced colitis mice model. (**B** to **D**) Body weight (B), macroscopic evaluation (C), and colon length (D) of mice were assessed [*n* = 4, means ± SEM; ns, not significant (*P* > 0.05); *P = 0.0140; and **P < 0.01 (Shapiro-Wilk test, *P* > 0.1; *F* test, *P* > 0.1), two-tailed unpaired Student’s *t* test]. (**E**) Representative H&E staining images of colon tissues from mice on day 42. Scale bars, 200 μm. (**F**) Schematic illustration of the study design of DC@CFO/BFO nanoparticle-pretreated T_H_2 cells administration in CIA mouse model. (**G**) Arthritis score of arthritic animals were assessed every other day (*n* = 5, means ± SEM; **P = 0.0012 and ***P = 0.0004, two-tailed unpaired Student’s *t* test). (**H**) Representative images including gross photographs, micro–computed tomography (micro-CT) images, H&E staining pictures, toluidine blue (TB) staining images, and safranin-O/fast green (SafO) staining images of ankle joints were shown. Scale bars, 200 μm. (**I** and **J**) Paw thickness of arthritic mice (*n* = 5) and bone mineral density (BMD) of ankle joints (*n* = 8) were measured [means ± SEM; ns, not significant (*P* > 0.05); *P = 0.0329; **P < 0.01; and ***P = 0.0003 (Shapiro-Wilk test, *P* > 0.1; F test, *P* > 0.1), two-tailed unpaired Student’s *t* test]. (**K**) Proteoglycan loss of ankle joints was measured according to safranin-O/fast green staining [*n* = 4, means ± SEM, ns, not significant (*P* > 0.05) and **P = 0.0051, Kruskal-Wallis test]. (**L**) Anti-CII–specific antibodies were quantified by enzyme-linked immunosorbent assay (ELISA) [*n* = 4, means ± SEM; ns, not significant (*P* > 0.05); *P < 0.05; **P = 0.0025; ***P < 0.001; and ****P < 0.0001, two-tailed unpaired Student’s *t* test]. i.v., intravenous, i.p., intraperitoneal; i.d., intradermal.

T_H_2 cells have been reported to exert immunoregulatory functions by limiting T_H_1-mediated immune responses. To investigate whether magnetoelectricity-responsive T_H_2 cells have similar properties, we adoptively transferred CD4^+^CD45RB^hi^ naïve T cells into CB-17 severe combined immunodeficient (SCID) mice to induce colitis. In parallel, murine naïve CD4^+^ T cells were treated with DC@CFO/BFO nanocomposites and exposed to magnetic field stimulation under T_H_2-polarizing conditions. These treated T_H_2 cells were subsequently adoptively transferred into colitic mice after disease onset (Fig. 5A). Mice receiving magnetoelectricity-responsive T_H_2 cells exhibited markedly reduced colonic inflammation, as evidenced by less weight loss, longer colon length, and improved histological features on hematoxylin and eosin (H&E) staining (Fig. 5, B to E).

Beyond colitis, we further assessed the therapeutic potential of magnetoelectric nanocomposites in the collagen-induced arthritis (CIA) model, a widely used murine model of inflammatory arthritis. The progression of disease was dynamically monitored following T_H_2 cell adoptive transfer (Fig. 5F). Consistent with the findings in colitis, the adoptive transfer of Th2 cells treated with DC@CFO/BFO nanoparticles under magnetic field stimulation significantly reduced both the incidence and severity of arthritis, as determined by clinical scoring (Fig. 5G), gross examination, and histopathology (Fig. 5, H to K). In addition, mice receiving DC@CFO/BFO+M-treated T_H_2 cells exhibited reduced levels of anti–collagen type II (anti-CII) antibodies, further supporting the anti-inflammatory effect of the treatment (Fig. 5L). Together, these results demonstrate that magnetoelectricity-responsive T_H_2 cells exert potent immunoregulatory effects and ameliorate inflammatory disease in vivo.

### TAF9B is required for DC@CFO/BFO nanoparticle-mediated T_H_2 cell regulation

**Fig. 6.**
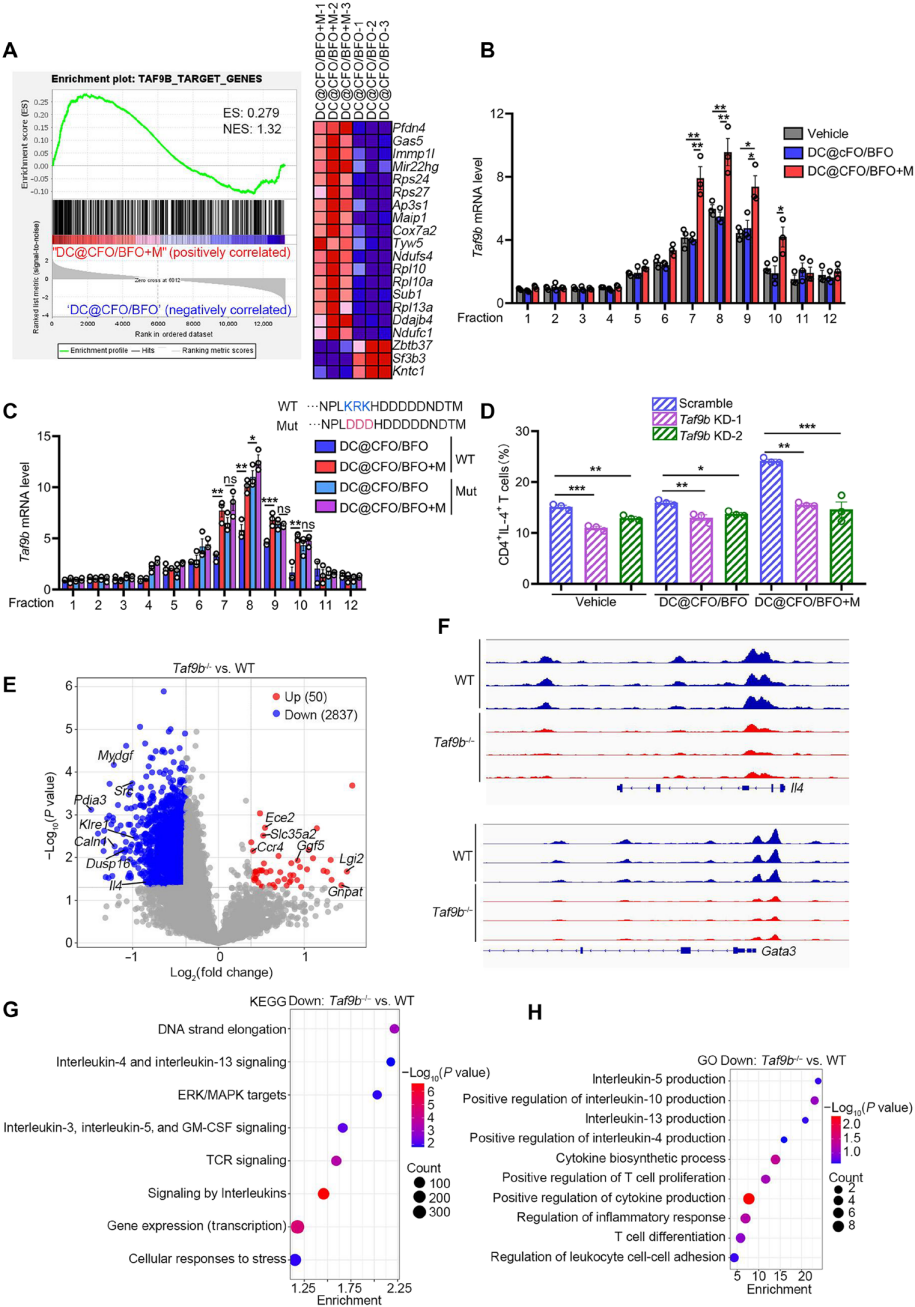
TAF9B is required for magnetoelectric nanoparticle–mediated T cell response. (**A**) CD4^+^ T cells were activated with antibodies against CD3/CD28 and induced to differentiate into T_H_2 cells. These cells were then treated with DC@CFO/BFO nanocomposites in the presence or absence of magnetic field stimulation. GSEA of genes expressed in CD4^+^ T cells under T_H_2-skewing condition treated with DC@CFO/BFO nanocomposites in the presence or absence of magnetic field (*n* = 3 biological replicates). (**B** and **C**) mRNA levels of WT *Taf9b* or mutant *Taf9b* in each fraction derived from polysome profiling were detected by quantitative real-time PCR. Fractions 6 to 12 were translationally active polysome fractions [*n* = 3, means ± SEM; ns, not significant (*P* > 0.05); *P < 0.05; **P < 0.01; and ***P = 0.0007 (Shapiro-Wilk test, *P* > 0.1; Brown-Forsythe test, *P* > 0.1), one-way ANOVA]. (**D**) Naïve CD4^+^ T cells were activated with antibodies against CD3/CD28 and infected with retrovirus containing *Taf9b* siRNA. T cells were cultured under T_H_2-polarizing condition with or without DC@CFO/BFO nanocomposites and magnetic field treatment and then restimulated for 5 hours with PMA and ionomycin. Proportion of CD4^+^IL-4^+^ cells in different group was analyzed by flow cytometry [*n* = 3, means ± SEM; *P = 0.018, **P < 0.01, and ***P < 0.001 (Shapiro-Wilk test, *P* > 0.1; Brown-Forsythe test, *P* > 0.1), one-way ANOVA]. (**E**) Volcano plot analysis of pairwise comparison of ATAC-seq density between WT and *Taf9b*^−/−^ T cells. (**F**) Integrative Genomics Viewer analysis of ATAC-seq coverage peaks at the *Il4* and *Gata3* locus in WT and *Taf9b*^−/−^ T cells. (**G**) ATAC peaks down-regulated in the *Taf9b*^−/−^ T cells were analyzed with KEGG database. (**H**) Genes that were significantly down-regulated in *Taf9b*^−/−^ T cells, compared with WT T cells, were analyzed using DAVID with GO terms. ERK, extracellular signal–regulated kinase; MAPK, mitogen-activated protein kinase.

To uncover the molecular mechanism by which magnetoelectric stimulation regulates T_H_2 differentiation, we performed gene set enrichment analysis (GSEA) on transcriptomic data and identified the transcription initiation factor TAF9B as a candidate of interest (Fig. 6A). The analysis of the Human Protein Atlas database revealed that TAF9B expression is largely restricted to T cell subsets (fig. S11A). Quantitative proteomics confirmed that TAF9B expression was up-regulated upon treatment with DC@CFO/BFO nanoparticles in the presence of an external magnetic field (fig. S11B). Polysome profiling of T cell lysates revealed increased levels of TAF9B mRNA in translationally active polysome fractions (fractions 6 to 12) following DC@CFO/BFO+M treatment (Fig. 6B). To assess whether this translational up-regulation depended on ribosomal electrostatics, we substituted the C-terminal basic amino acids (arginine and lysine) of TAF9B with the acidic amino acid aspartic acid. Polysome profiling combined with quantitative polymerase chain reaction (PCR) showed diminished translation efficiency of the mutant TAF9B under DC@CFO/BFO+M treatment (Fig. 6C), suggesting that electrostatic interactions at the ribosomal exit tunnel contribute to magnetoelectric modulation of TAF9B translation.

To evaluate the role of TAF9B in T cell function, we silenced TAF9B expression in murine CD4^+^ T cells (fig. S11C) and assessed their response to DC@CFO/BFO+M treatment. TAF9B knockdown significantly reduced T_H_2 polarization induced by magnetoelectric nanoparticles (Fig. 6D) while increasing the proportions of T_H_1 and T_H_17 cells (fig. S11, D and E). iT_reg_ cell proportions remained unchanged (fig. S11F). Furthermore, T_H_2 proliferation was markedly impaired upon TAF9B knockdown, as indicated by reduced Ki-67 expression (fig. S11G). These findings suggest that TAF9B is essential for T_H_2 cell differentiation and expansion in response to DC@CFO/BFO+M treatment.

To further investigate TAF9B function, we generated *Taf9b* knockout mice (fig. S11, H and I) and performed assay for transposase-accessible chromatin sequencing (ATAC-seq) and RNA-seq analyses. ATAC-seq profiles showed that TAF9B deficiency led to global reductions in chromatin accessibility, particularly around transcription start sites (TSSs) (Fig. 6E and fig. S12, A and B). Key T_H_2-related genes including *Il4*, *Gata3*, *Il13*, and *Stat6* exhibited decreased accessibility in *Taf9b^−/−^* T cells (Fig. 6F and fig. S12C). In wild-type T cells, open chromatin regions were enriched for genes involved in DNA replication, interleukin signaling, and TCR signaling (Fig. 6G and fig. S12D), whereas *Taf9b*-deficient cells showed increased accessibility at loci linked to protein localization and ion transport (fig. S12E). Transcriptomic analysis further revealed 1493 up-regulated and 1460 down-regulated genes in *Taf9b^−/−^* T cells (fig. S13, A and B). GO enrichment highlighted down-regulation of genes involved in interleukin production and T cell differentiation (Fig. 6H and fig. S13C), while pathways associated with fatty acid metabolism and T cell apoptosis were up-regulated (fig. S13D). Collectively, these findings establish TAF9B as a critical regulator of T cell differentiation and function, essential for mediating the immunoregulatory effects of DC@CFO/BFO nanoparticles under magnetic stimulation.

### Biodistribution profiles and safety evaluation of DC@CFO/BFO nanoparticle

Given the ability of DC@CFO/BFO nanoparticles to promote T_H_2 cell differentiation in vitro, we next investigated their potential to exert similar effects in vivo. To assess biodistribution, FITC-labeled DC@CFO/BFO nanoparticles were intravenously injected into C57BL/6 mice. In vivo imaging revealed that the nanoparticles accumulated in the liver, kidney, lung, and spleen and persisted in these organs for up to 4 days postinjection (fig. S14, A and B). Consistent with the low cytotoxicity observed in vitro, the administration of a high dose (50 mg/kg) of DC@CFO/BFO nanoparticles, with or without magnetic field stimulation, had minimal effects on murine peripheral blood cells at day 7 postinjection (fig. S14, C to Q). In addition, treatment with DC@CFO/BFO nanoparticles did not significantly alter key liver and kidney function parameters (fig. S15, A to F), and the histological analysis of major organs revealed no notable abnormalities (fig. S15G). These results demonstrate a favorable safety profile and biocompatibility of DC@CFO/BFO nanoparticles in vivo, supporting their further application for immunomodulation.

### DC@CFO/BFO nanoparticles exert anti-inflammatory effects under external magnetic field

**Fig. 7.**
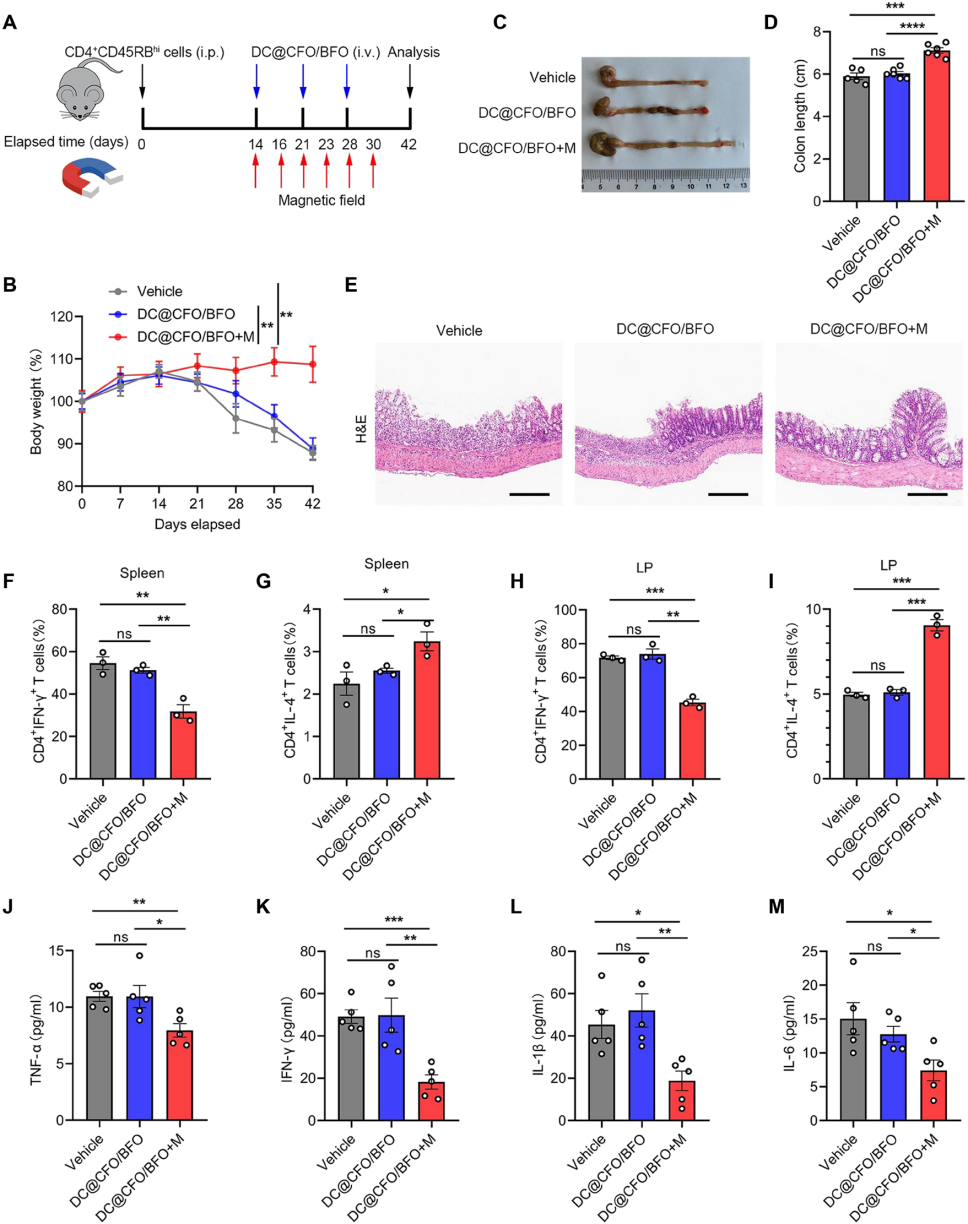
Magnetoelectric nanoparticles inhibit T cell transfer–induced colitis under external magnetic field stimulation. (**A**) Schematic illustration of the study design of DC@CFO/BFO nanoparticle treatment in the T cell transfer–induced colitis mouse model. (**B**) Body weights of mice from different treatment groups were assessed [vehicle, *n* = 5; DC@CFO/BFO, *n* = 6; DC@CFO/BFO+M; *n* = 6, means ± SEM; **P < 0.01 (Shapiro-Wilk test, *P* > 0.1; *F* test, *P* > 0.1), two-tailed unpaired Student’s *t* test]. (**C** and **D**) Macroscopic evaluation (C) and colon length (D) of mice from different treatment groups on day 42 [vehicle, *n* = 5; DC@CFO/BFO, *n* = 6; DC@CFO/BFO+M; *n* = 6, means ± SEM; ns, not significant (*P* > 0.05); ***P = 0.01; and ****P < 0.0001 (Shapiro-Wilk test, *P* > 0.1; *F* test, *P* > 0.1), two-tailed unpaired Student’s *t* test]. (**E**) Representative H&E staining images of colon tissues from mice of different treatment groups on day 42. Scale bars, 200 μm. (**F** and **G**) Flow cytometric analysis of the proportion of CD4^+^IFN-γ^+^ cells and CD4^+^IL-4^+^ cells in mice spleen from different treatment groups [*n* = 3, means ± SEM; *P < 0.05 and **P < 0.01 (Shapiro-Wilk test, *P* > 0.1; F test, *P* > 0.1), two-tailed unpaired Student’s *t* test]. (**H** and **I**) Flow cytometric analysis of the proportion of CD4^+^IFN-γ^+^ cells and CD4^+^IL-4^+^ cells in mice lamina propria (LP) [*n* = 3, means ± SEM; **P = 0.0013 and ***P < 0.001 (Shapiro-Wilk test, *P* > 0.1; *F* test, *P* > 0.1), two-tailed unpaired Student’s *t* test]. (**J** to **M**) Mice serum was obtained and inflammatory cytokines were detected by ELISA [*n* = 5, means ± SEM; ns, not significant (*P* > 0.05); *P < 0.05; **P < 0.01; and ***P < 0.001 (Shapiro-Wilk test, *P* > 0.1; *F* test, *P* > 0.1), two-tailed unpaired Student’s *t* test].

We next examined whether DC@CFO/BFO nanoparticles could mitigate inflammation in vivo when combined with repeated magnetic field stimulation. In a murine colitis model, recipient mice received intravenous administration of DC@CFO/BFO nanoparticles with or without magnetic field loading (Fig. 7A). Mice in the vehicle and DC@CFO/BFO groups developed wasting disease and severe colitis, while the DC@CFO/BFO+M group showed notably attenuated disease progression, as evidenced by reduced body weight loss and longer colon length (Fig. 7, B to D). Histological analysis further revealed diminished colonic inflammation and reduced immune cell infiltration in the DC@CFO/BFO+M group (Fig. 7E). The immune profiling of spleen and colonic lamina propria (LP) demonstrated increased frequencies of IL-4–producing CD4^+^ T cells and decreased IFN-γ^+^ and IL-17A^+^ T cells in mice treated with DC@CFO/BFO+M (Fig. 7, F to I, and fig. S16, A to D). Correspondingly, levels of key inflammatory cytokines were significantly reduced (Fig. 7, J to M).

**Fig. 8.**
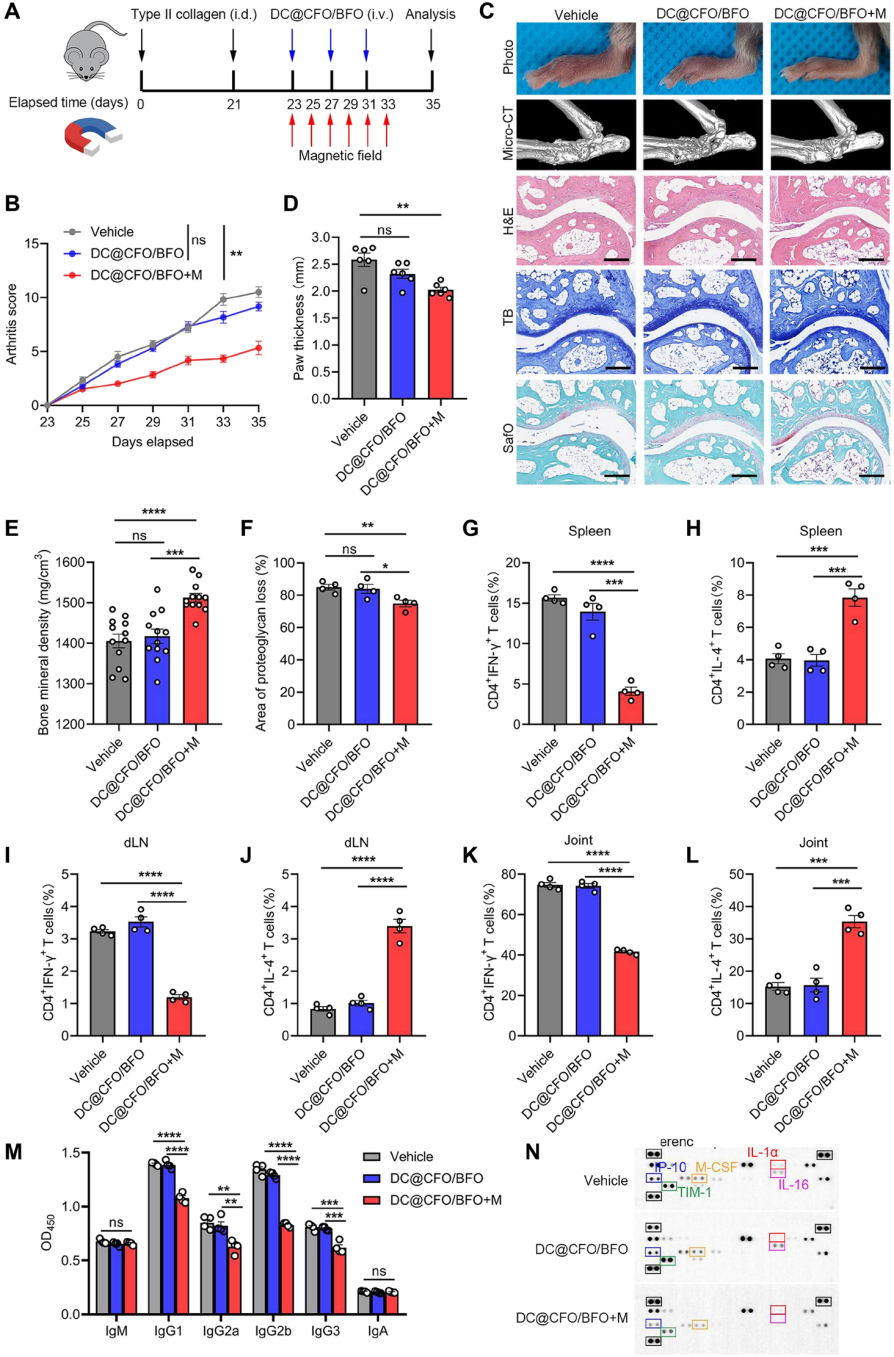
Magnetoelectric nanoparticles ameliorate CIA under external magnetic field stimulation. (**A**) Graphic illustration of the study design of DC@CFO/BFO nanoparticles treatment in a CIA mouse model. (**B**) Arthritis score of arthritic animals were assessed every other day [*n* = 6, means ± SEM; ns, not significant (*P* > 0.05) and **P = 0.0015, Kruskal-Wallis test]. (**C**) Representative images including gross photographs, micro-CT images, H&E staining images, toluidine blue staining images, and safranin-O/fast green staining images of ankle joints were shown. Scale bars, 200 μm. (**D**) Paw thickness of arthritic mice were measured [*n* = 6, means ± SEM; ns, not significant (*P* > 0.05) and **P = 0.0051, Kruskal-Wallis test]. (**E** and **F**) BMD of ankle joints (*n* = 12) (E) and proteoglycan loss of ankle joints (*n* = 4) (F) from different treatment groups [means ± SEM; ns, not significant (*P* > 0.05); *P = 0.0366; **P = 0.0089; ***P = 0.0001; and ****P < 0.0001, (Shapiro-Wilk test, *P* > 0.1; *F* test, *P* > 0.1), two-tailed unpaired Student’s *t* test]. (**G** to **L**) Flow cytometric analysis of the proportion of CD4^+^IFN-γ^+^ cells and CD4^+^IL-4^+^ cells in mice spleen [(G) and (H)], draining LN (dLN) [(I) and (J)], and ankle joints [(K) and (L)] from different treatment groups [*n* = 4, means ± SEM; ***P < 0.001 and ****P < 0.0001 (Shapiro-Wilk test, *P* > 0.1; *F* test, *P* > 0.1], two-tailed unpaired Student’s *t* test). (**M**) Mice serum was obtained, and anti-CII–specific antibodies were measured by ELISA [*n* = 4, means ± SEM; ns, not significant (*P* > 0.05); **P < 0.01; ***P < 0.001; and *P* < 0.0001 (Shapiro-Wilk test, *P* > 0.1; *F* test, *P* > 0.1), two-tailed unpaired Student’s *t* test]. (**N**) Mice serum was obtained, and cytokine array was performed to evaluate inflammatory factors.

We further validated the therapeutic potential of DC@CFO/BFO nanoparticles in a CIA model (Fig. 8A). Mice treated with DC@CFO/BFO+M showed reduced arthritis scores post disease onset (Fig. 8B). Gross observations and micro–computed tomography (micro-CT) revealed alleviated joint inflammation, preserved joint structure, and restored bone mineral density in the DC@CFO/BFO+M group (Fig. 8, C to E). The histological analysis confirmed reduced synovitis and protection against cartilage and bone destruction (Fig. 8F). Flow cytometry consistently showed increased IL-4^+^ CD4^+^ T cells and decreased IFN-γ^+^ CD4^+^ T cells in the spleen (Fig. 8, G and H, and fig. S17A), draining lymph nodes (Fig. 8, I and J, and fig. S17B), and ankle joints (Fig. 8, K and L, and fig. S17C) of DC@CFO/BFO+M-treated mice. Moreover, a modest reduction in IL-17A^+^ T cells and an increase in FOXP3^+^ T cells were observed in CIA mice following DC@CFO/BFO+M treatment (fig. S18, A to F). DC@CFO/BFO+M treatment also reduced serum levels of pathogenic anti-CII immunoglobulin G (IgG) and pro-inflammatory cytokines (Fig. 8, M and N, and fig. S18G). Since T peripheral helper (Tph) cells and Tfh cells have been shown to promote the formation of tertiary lymphoid structures within inflamed tissues, thereby facilitating local B cell activation and antibody production (*37*–*40*), we further analyzed the proportion of these cells in arthritic joint and observed no significant changes in Tph or Tfh cells within CIA ankle joints (fig. S18, H to J), suggesting that local Tph/Tfh expansion may not be a major feature in this model.

To further assess the therapeutic relevance of magnetoelectric nanoparticles, we performed additional experiments in the CIA model by initiating treatment at the fifth week, after clinical disease had been established (fig. S19A). Consistent with our earlier results, DC@CFO/BFO+M treatment significantly reduced both the incidence and severity of arthritis, as assessed by clinical scores (fig. S19B), gross examination of joints, and histopathological analysis (fig. S19, C to E). These findings demonstrate that the therapeutic effect of DC@CFO/BFO+M is not restricted to prophylactic settings but also extends to established disease. In addition, we performed cytokine blockade experiments using a neutralizing antibody against IL-4 to directly evaluate T_H_2 dependency. In T cell transfer-induced colitis, the protective effect of DC@CFO/BFO+M treatment was largely abrogated in mice receiving anti–IL-4, as evidenced by increased body weight loss, shortened colonic length, and impaired histological outcomes (fig. S20, A to D). Similar results were observed in the CIA model (fig. S20, E to G). Together, these findings confirm that DC@CFO/BFO nanoparticles, when coupled with magnetic field stimulation, elicit robust anti-inflammatory responses in both colitis and arthritis models, largely through the induction of T_H_2 cell–mediated immune regulation.

### TAF9B is required for therapeutic effect of magnetoelectric nanoparticles under an external magnetic field

**Fig. 9.**
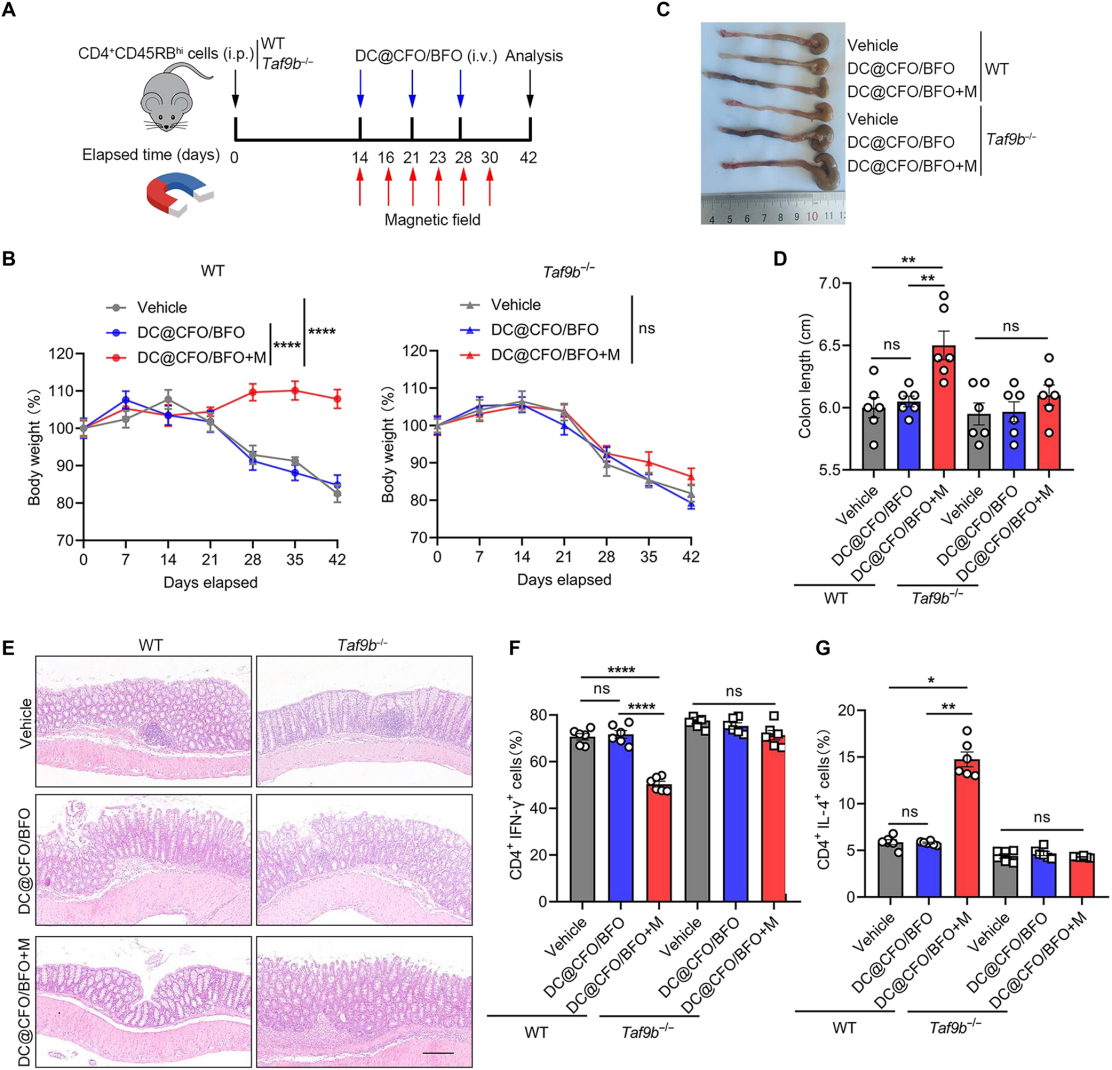
TAF9B is critical for therapeutic role of magnetoelectric nanoparticle upon on magnetic field stimulation. (**A**) Schematic illustration of the study design of DC@CFO/BFO nanoparticle treatment in the T cell transfer–induced colitis mouse model. Briefly, CB17-SCID mice were intraperitoneally injected with WT or *Taf9b*^−/−^ CD4^+^CD45RB^hi^ cells to induce colitis. DC@CFO/BFO nanoparticles (10 mg/kg) were intravenously injected into mice, and 1.5-mT meganetic field was loaded for 30 min every day after nanoparticle injection at indicated time points. (**B**) Body weights of mice from different treatment groups were assessed every week [*n* = 6, means ± SEM; ns, not significant (*P* > 0.05) and ****P < 0.0001 (Shapiro-Wilk test, *P* > 0.1; Brown-Forsythe test, *P* > 0.1), one-way ANOVA]. (**C** and **D**) Macroscopic evaluation (C) and colon length (D) of mice from different treatment groups on day 42 [*n* = 6, means ± SEM; ns, not significant (*P* > 0.05) and **P < 0.01 (Shapiro-Wilk test, *P* > 0.1; Brown-Forsythe test, *P* > 0.1), one-way ANOVA]. (**E**) Representative H&E staining images of colon tissues from mice of different treatment groups on day 42. Scale bars, 200 μm. (**F**) Flow cytometric analysis of the proportion of CD4^+^IFN-γ^+^ cells in mice LP from different treatment groups [*n* = 6, means ± SEM; ns, not significant (*P* > 0.05) and ****P < 0.0001 (Shapiro-Wilk test, *P* > 0.1; Brown-Forsythe test, *P* > 0.1), one-way ANOVA]. (**G**) Flow cytometric analysis of the proportion of CD4^+^IL-4^+^ cells in mice LP from different treatment groups [*n* = 6, means ± SEM; ns, not significant (*P* > 0.05); *P = 0.0206; and **P = 0.0051, Kruskal-Wallis test].

To assess the essential role of TAF9B in vivo, we used *Taf9b* gene knockout mice and conducted T cell transfer experiments. In detail, we adoptively transferred wild-type (WT) or *Taf9b*^−/−^ CD4^+^CD45RB^hi^ naïve T cells into CB-17 SCID mice to induce colonic inflammation, and the recipient mice were administrated with DC@CFO/BFO nanoparticles along with magnetic field treatment (Fig. 9A). As determined by body weight loss and shortened colonic length, DC@CFO/BFO+M treatment significantly ameliorated colitis development in mice receiving WT CD4^+^CD45RB^hi^ T cells, whereas DC@CFO/BFO+M treatment exhibited little effect in those receiving *Taf9b*-deficient T cells (Fig. 9, B to D). Histological analysis also revealed that *Taf9b* deficiency reduced T cell response to DC@CFO/BFO+M treatment and impaired the anti-inflammatory effect of magnetoelectric nanoparticles plus magnetic field stimulation (Fig. 9E). Accordingly, DC@CFO/BFO+M failed to induce the T_H_2 phenotype in *Taf9b*^−/−^ CD4^+^ T cells (Fig. 9, F and G). Moreover, we pretreated murine WT or *Taf9b*^−/−^ naïve CD4^+^ T cells with DC@CFO/BFO nanocomposites and exposed to magnetic field stimulation under T_H_2-polarizing conditions. These prepolarized T_H_2 cells were then transferred into colitic mice after disease onset. Mice receiving WT T_H_2 cells showed significantly reduced colonic inflammation, reflected by attenuated body weight loss, preserved colon length, and improved histological features (fig. S21, A to D). In contrast, the transfer of *Taf9b*^−/−^ T_H_2 cells pretreated with DC@CFO/BFO+M failed to confer protection, demonstrating that TAF9B is indispensable for the therapeutic function of magnetoelectric nanoparticle–induced T_H_2 cells (fig. S21, A to D). The above data thus demonstrated the crucial role of TAF9B in the magnetoelectric nanoparticle–mediated T cell response.

## DISCUSSION

Magnetoelectric nanoparticle–based biomaterials exhibit much potential for clinical applications because of their unique capacity for tuning electrical properties via modulation of magnetic field parameters (*41*–*43*). The efficacy of magnetoelectric nanoparticles in promoting tissue regeneration and mesenchymal stem cell differentiation has been widely demonstrated (*44*–*48*). Nevertheless, little is known about the role of magnetoelectric nanocomposites in T cell–mediated immune response. Our present study reports that DC membrane–camouflaged magnetoelectric nanocomposites regulate T_H_1/T_H_2 paradigm and promote T_H_2 cell proliferation, thereby ameliorating T cell–mediated autoimmune response and maintaining host immune homeostasis.

Upon TCR engagement, naïve CD4^+^ T cells undergo differentiation into various lineages of T_H_ cells, with transcription factors playing a central role in determining lineage commitment within specific cytokine environments. TFIID components, known as TBP-associated factors (TAFs), are essential for preinitiation complex assembly and RNA polymerase II (Pol II)–mediated transcription initiation (*49*, *50*). Among these, TAF9B has been identified as a critical regulator of gene expression associated with apoptosis and essential for cell viability (*51*, *52*). It binds to both promoters and distal enhancers of neuronal genes, governing efficient motor neuron differentiation (*53*). Our multiomics analysis identified TAF9B as a pivotal protein regulated by DC@CFO/BFO nanoparticles and magnetic field stimulation, intimately linked with ribosomal interactions and protein structure. Unlike other genes modulated by these stimuli, TAF9B functions as a transcription factor. Prior research has elucidated TAF9B’s role in mediating the transcription of genes involved in metabolism, translation, and ribosomal protein synthesis (*53*), corroborating our findings. To validate our hypothesis, we depleted endogenous TAF9B in T cells, which resulted in a notable inhibition of T_H_2 cell polarization. In addition, we acknowledged the involvement of other genes—such as *Cdkn2c*, *Selenoi*, and *Lsm5*—in T_H_2 cell differentiation (*54*–*56*), suggesting that DC@CFO/BFO nanoparticles and magnetic field stimulation may affect this process through multiple pathways. However, our results underscore the critical importance of TAF9B activation as a primary mechanism in T_H_2 cell regulation. These findings enhance our understanding of the molecular mechanisms governing T_H_2 cell differentiation and highlight the therapeutic potential of targeting TAF9B. Further investigation is warranted to elucidate the intricate interplay between TAF9B and other regulatory genes in determining T_H_2 cell fate.

T cells undergo major transitions in protein synthesis activity upon activation, proliferation, and differentiation. The precise regulation of protein synthesis shapes the functional capabilities of T cell immune responses. There is evidence that translation of transcripts encoding ribosomal proteins is regulated during the differentiation of effector T cells, and translational suppression was more pronounced in terminal effector cells (*25*). The intracellular pathway of nanoparticles after cellular uptake is as follows: “endocytosis–endosome–fusion with lysosomes–lysosomal escape–entry into the cytoplasm–functional efficacy” (*57*, *58*). Designing nanoparticles capable of efficiently achieving endosomal/lysosomal escape remains one of the central challenge in nanomedicine. In our system, two mechanisms may contribute to cytoplasmic access: (i) Under an external magnetic field, the DC@CFO/BFO particles exhibit dynamic surface charge modulation, which can destabilize endosomal membranes and favor escape; and (ii) the biomimetic cell membrane coating is likely to mediate transient fusion or pore formation with endosomal/lysosomal membranes, facilitating release into the cytoplasm. These combined effects may explain how the nanocomposites bypass endosomal sequestration and ultimately engage ribosomes. In future work, we will further validate this proposed mechanism by directly tracking endosomal escape using pH-sensitive fluorescent probes and transmission electron microscopy and by implementing rational surface modifications (e.g., pH-responsive or fusogenic peptides) to enhance escape efficiency and targeting specificity, thereby promoting their regulatory efficacy in T cell differentiation.

T cells generate ROS during activation and proliferation, and oxidative stress is known to influence cellular metabolism. Magnetoelectric nanoparticles can catalyze the generation of ROS under an applied magnetic field and whether magnetic field–induced ROS may provide an alternative mechanism need further investigation. In this study, we found that magnetoelectric nanocomposites exhibited colocalization with ribosomal components and optimized amino acid metabolic fitness. By combining polysome profiling and proteome analysis, we examined the dynamics of protein synthesis and revealed that magnetoelectric nanocomposites regulated electrostatic interactions at ribosome and stimulated translation upon magnetic field loading, thereby facilitating T_H_2 cell polarization and proliferation.

The type 1 and type 2 immune response paradigm describes distinct immune responses, and the balance of T_H_ subsets plays an essential role in the regulation of host autoimmune responses. Recent studies illustrated that utilization of nanoparticle-based magnetoelectric biomaterials can potentially lead to future precision treatment and prevention of cancer, neurodegenerative disorders, and infectious diseases (*59*–*62*). However, most magnetoelectric particles lack cell-specific targeting, and the effects of magnetoelectric particles on T_H_ cell–mediated response are largely unknown. We thus used a cell membrane coating approach to camouflage magnetoelectric composites to achieve CD4^+^ T cell targeting. These biomimic magnetoelectric nanoparticles orchestrated T cell differentiation and proliferation and ameliorated host autoimmune responses in both the mouse CIA and T cell transfer-induced colitis models upon stimulation with an external magnetic field.

Together, our findings demonstrated that the DC membrane–camouflaged magnetoelectric nanoparticles regulated T cell polarization and inhibited T cell–mediated autoimmune responses, thus opening up an avenue for T cell biology and exhibiting promising development potential for clinical applications.

## MATERIALS AND METHODS

### Mice

DBA/1 mice and CB-17 SCID mice were purchased from Vital River Laboratory Animal Technology. *Taf9b*^─/─^ mice were purchased from GemPharmatech. All animals were housed and maintained under specific pathogen–free conditions. All sex- and age-matched animal experiments were performed in accordance with protocols approved by the Ethics Committee of Peking University Health Science Center (approved number PUIRB-LA2024005).

### Fabrication of CFO/BFO and DC@CFO/BFO nanoparticles

For the fabrication of the core layer structure CFO nanoparticles, 0.092 M FeCl_3_, 0.046 M CoCl_2_, and 0.14 M cetyltrimethylammonium bromide (CTAB) were dissolved in deionized water. Then, 6 M NaOH was added to the above solution with thorough mechanical stirring and ultrasonic dispersion before transfer of the resultant solution to a sealed polytetrafluoroethylene lined hydrothermal reactor and heated at 200°C for 24 hours. The black powder obtained was then washed with deionized water and ethanol, followed by drying overnight at 95°C.

For the fabrication of core-shell CFO/BFO nanoparticles, 0.011 M Bi(NO_3_)·5H_2_O and 0.01 M Fe(NO_3_)·9H_2_O were dissolved in ethylene glycol to prepare the precursor of BFO. A 0.15 g of dried CFO nanoparticles were then dispersed into 100 ml of BFO precursor solution and sonicated for 3 hours. The solution was then dried overnight, followed by annealing the dried powder at 600°C for 2.5 hours with a heating ramp rate of 10°C min^−1^. Last, the annealed powder was sonicated for 30 min and filtered through a 0.22-μm filter membrane to obtain the magnetoelectric core-shell CFO/BFO nanocomposites suitable for biomedical applications.

To obtain DC membrane–coated CFO/BFO nanocomposites, bone marrow derived from murine femur and tibia was first isolated, and recombinant granulocyte-macrophage colony-stimulating factor (20 ng/ml) and recombinant IL-4 (10 ng/ml) were used to induce DC differentiation. DCs (CD11c^+^MHC II^+^ cells) were then sorted by flow cytometry. The harvested DCs were prepared by three cycle of freezing and thawing in liquid nitrogen, and the lysate was sonicated for 5 min by a bath sonicator. The resulting vesicles were subsequently extruded serially through a 400- and then 200-nm polycarbonate porous membranes using an Avanti mini extruder.

### Characterization of CFO/BFO nanoparticles and DC@CFO/BFO nanoparticles

The surface morphology of the CFO/BFO nanoparticles was characterized by scanning electron microscopy (SEM) (JSM-7001F, Japan). The structures of core-shell CFO/BFO nanoparticles and DC membrane–coated CFO/BFO composites were observed with high-resolution transmission electron microscopy (TEM) (HRTEM, JEM-2100, JEOL, Japan). The composition with CFO/BFO nanoparticles was examined by EDS spectra with TEM (HRTEM, JEM-2100, JEOL, Japan) or XRD spectroscopy (XRD, Rigaku D/max 2500 VB2t/PC, Japan). PFM measurements were recorded by a commercial atomic force microscope (NTEGRA, NT-MDT, Russia) equipped with a ferroelectric test system. Measurements were made with a VSM on a Quantum Design PPMS-9 DynaCool system. A 1 mg of nanoparticles was placed in 200 μl of ddH_2_O or dimethyl sulfoxide with or without magnetic field stimulation or ultrasound treatment for ·OH and ·O_2_^−^ detection, respectively. A 20 μl of DMPO was added into the solution, and the reactive species created by the nanocomposites were detected by the EPR technique (ELEXSYS-E580, Bruker). The size and surface potential of the nanoparticles were measured by a Zeta Sizer Nano-ZS Instrument (Malvern Instruments, Worcestershire, WR, UK) in an aqueous environment at room temperature.

### FEA method

FEA simulations were conducted using the COMSOL software (COMSOL Multiphysics 6.1a) to model the generation of electric potential induced on the surface of the CFO/BFO nanoparticle with and without a magnetic field. The numerical models consist of three concentric spheres. The innermost is the magnetically permeable CFO/BFO nanoparticle. The middle spherical shell volume represents free space; this is set as the analysis domain and is the region of interest investigated in the models. The outside shell volume represents a region extending to infinity, modeled with an infinite element domain. When using infinite element domain features, the boundary condition on the exterior of the modeling domain does not significantly affect the solution. Specifically, to study the electromagnetic coupling, the magnetic physics field was meticulously considered. Furthermore, it is essential to simulate and analyze the physical model of the cell membrane–coated CFO/BFO nanoparticles, taking into account that the actual nanomaterial surface is enveloped by a layer of biological cell membrane medium. Incorporating the cell membrane medium into the existing model facilitates a comprehensive understanding of how this biological material influences the electrical properties of the nanoparticles under both the presence and absence of a magnetic field. The physical parameters (e.g., relative magnetic permeability, electrical conductivity, dielectric constant, etc.) of cell membrane, BFO, and CFO materials were derived from relevant references and experimental results (*61*, *63*–*66*). The geometrical configurations of the nanoparticle components were meticulously designed in accordance with specified requirements.

### Immunoblot analysis

Cells were lysed by radioimmunoprecipitation assay lysis buffer [50 mM tris (pH 7.4), 150 mM NaCl, 1% Triton X-100, 1% sodium deoxycholate, and 0.1% SDS] supplemented with protease inhibitor cocktail (Roche) and subjected to SDS–polyacrylamide gel electrophoresis. Antibodies used in this study were as follows: anti-CD11b (1:1000; Abcam, ab133357), anti–β-catenin (1:5000; Abcam, ab32572), anti-HDAC1 (1:1000; Santa Cruz Biotechnology, sc-81598), anti-puromycin (1:1000; Merck Millipore, MABE341), anti-TAF9B (1:1000; Signalway Antibody, 45238), and anti–glyceraldehyde-3-phosphate dehydrogenase (1:5000; RayAntibody, RM2002).

### Sample preparation and flow cytometric analysis

Immune cells from spleen or lymph nodes were isolated by grinding the tissues in phosphate-buffered saline containing 1% (v/v) fetal bovine serum, followed by filtering through a cell strainer. To analyze immune cells in joints, ankles were cut from 3 mm above the heel until mid-paw, minced, and incubated in Dulbecco’s modified Eagle’s medium containing collagenase A (1 mg/ml; Roche) at 37°C for 1 hour with occasional mixing. Dissociated cells were then washed and filtered through a cell strainer.

For lamina propria mononuclear cells isolation, mice intestines were removed and cut into 1.5-cm pieces. The pieces were then incubated in Hanks’ balanced salt solution with 5 mM EDTA for 20 min at 37°C. After that, the intestinal epithelial cells were removed by intensive vortexing and passing through a 100-μm cell strainer. Small pieces of tissues were then placed in digestion solution containing collagenase D and deoxyribonuclease I at 37°C for 40 min with slow rotation. After passing through filters, LP lymphocytes were isolated by percoll reagent (*67*, *68*).

To analyze the surface proteins of DC@CFO/BFO nanocomposites, DC@CFO/BFO nanocomposites were incubated with specific antibodies for 30 min at room temperature and analyzed by a CytoFlex Flow Cytometer. To detect cellular uptake of nanoparticles, CFO/BFO nanoparticles and DC@CFO/BFO nanocomposites were mixed with FITC aqueous solution of the same quality, separately, and then stirred in the dark for 12 hours. After magnetic separation and washing with deionized water for three times to remove excess FITC, FITC-conjugated nanoparticles were incubated with murine naïve T cells or splenocytes for indicated time durations, and the FITC^+^ cells were then detected by a FACSVerse Flow Cytometer.

To detect T cell proliferation, the isolated T cells were stained with 1 μM CFSE for 10 min at room temperature. The cells were then induced to activation and proliferation and collected at indicated time points, followed by flow cytometric analysis.

To analyze cell surface maker expression (CD45, CD4, CD8, CD25, B220, CD11b, Ly6G, CD11c, MHC II, and F4/80), the cells were incubated with specific antibodies for 30 min at room temperature. To perform intracellular marker staining (IFN-γ, IL-4, and IL-17A), the cells were treated with protein transport inhibitor cocktail (eBioscience, 00-4980-03) for 5 hours and then fixed and permeabilized, followed by staining with specific antibodies. To detect the expression of nuclear protein (Ki-67, FOXP3), cells were fixed and permeabilized, followed by staining with specific antibodies.

The following antibodies were used: anti-CD45 (eBioscience, 30-F11), anti-CD4 (BioLegend, GK1.5), anti-CD8 (eBioscience, 53–6.7), anti-CD25 (BioLegend, 3C7), anti-B220 (BioLegend, RA3-6B2), anti-CD11b (Biolegend, M1/70), anti-Ly6G (eBioscience, RB6-8C5), anti-CD11c (BioLegend, N418), anti-F4/80 (BioLegend, BM8), anti–MHC II (BioLegend, I-A/I-E, M5/114.15.2), anti–programmed cell death protein 1 (PD-1) (BioLegend, RMP1-30), anti-CXCR5 (BioLegend, L138D7), anti–IFN-γ (BioLegend, XMG1.2), anti–IL-4 (eBioscience, 11B11), anti–IL-17A (eBioscience, eBio17B7), anti–Ki-67 (BioLegend, 16A8), and anti-FOXP3 (eBioscience, FJK-16s).

### Confocal microscopy

CD4^+^ T cells were incubated with FITC-labeled CFO/BFO nanoparticles and attached to glass coverslips coated with poly-l-lysine. Cells were fixed with 4% (w/v) paraformaldehyde, permeabilized with 0.5% (v/v) Triton X-100 and blocked by 1% (w/v) bovine serum albumin, followed by staining with specific antibodies and 4′,6-diamidino-2-phenylindole. Images were acquired with a Nikon TCS A1 microscope. Antibodies against RPS3 (1:100; Abcam, ab128996) and RPL7 (1:500; Abcam, ab72550) were used to label ribosomes. LysoTracker Red (Solarbio, L8010) was used to track lysosome.

### In vivo imaging for DC@CFO/BFO nanocomposite

To study the in vivo distribution of the nanocomposites, FITC-labeled DC@CFO/BFO nanoparticles (10 mg/kg) were intravenously injected into C57BL/6 mice. Murine heart, liver, kidney, lung, and spleen were isolated at indicated time points, followed by bioluminescent imaging using a Xenogen IVIS-200 instrument (PerkinElmer). The images were analyzed using Living Image software v.4.1 (PerkinElmer)

### Biosafety evaluation

To perform in vitro cell cytotoxicity assay, murine naïve CD4^+^ T cells were exposed to a concentration series of nanoparticles for 24 hours, with or without external magnetic field stimulation, and the viability of T cells was assessed by Cell Counting Kit-8 (CK04) according to the manufacturer’s protocol.

For in vivo biosafety evaluation, C57BL/6 mice were administrated with a high concentration (50 mg/kg) of DC@CFO/BFO nanocomposites with magnetic field stimulation for 30 min every day. After 7 days postinjection, blood biochemistry tests and blood routine tests were performed. Major organs were also harvested for histological analysis.

### In vitro T cell differentiation

Murine naïve CD4^+^ T cells (CD4^+^CD25^−^CD62L^hi^CD44^lo^) were sorted by flow cytometry from peripheral lymph nodes and were then activated with plate-bound anti-CD3 antibody (2 μg/ml) and anti-CD28 antibody (1 μg/ml). For T_H_1 polarization, anti–IL-4 antibody (10 μg/ml) and IL-12 (10 ng/ml) were used. For T_H_2 polarization, anti–IFN-γ antibody (10 μg/ml) and IL-4 (20 ng/ml) were used. For T_H_17 polarization, IL-6 (20 ng/ml), transforming growth factor–β (TGF-β) (5 ng/ml), anti–IFN-γ antibody (10 μg/ml), and anti–IL-4 antibody (10 μg/ml) were used. For iT_reg_ cell differentiation, TGF-β (1 ng/ml) and IL-2 (4 ng/ml), neutralizing antibody against IFN-γ (10 μg/ml), and neutralizing antibody against IL-4 (10 μg/ml) were used. For Tfh polarization, TGF-β (1 ng/ml) and IL-12 (5 ng/ml) were used. T cells were incubated with DC@CFO/BFO nanoparticles (10 μg/ml) and subjected to 1.5-mT magnetic field for 30 min every day. After 48 or 72 hours of culture, the cells were collected for flow cytometric analysis.

### Puromycin incorporation assay

CD4^+^ T cells were isolated and treated with magnetoelectric nanocomposites and magnetic field stimulation under T_H_2-skewing condition, followed by pulsing with puromycin (50 μg/ml) for 30 min. Puromycin-labeled proteins were identified with immunoblot analysis.

### Polysome profiling

CD4^+^ T cells were isolated and incubated with magnetoelectric nanocomposites and magnetic field treatment under T_H_2-skewing condition, followed by treatment with cycloheximide (100 μg/ml) for 10 min. The cells were then resuspended in hypotonic buffer [20 mM Hepes (pH 7.4), 100 mM KCl, 5 mM MgCl_2_, 1 mM dithiothreitol, 0.5% NP-40, protease inhibitor cocktail (EDTA-free), and ribonuclease inhibitor (200 U/ml)] and passed through a 23-gauge needle for three times. The resulting homogenate was centrifuged at 12,000 rpm for 10 min to clarify lysate. After centrifugation, the supernatant was transferred to a new tube, and absorbance was measured at 260 nm. The expected absorbance value is around 15 to 20 U/ml. The samples were then diluted with lysis buffer to the same absorbance value around 15 to 20 U/ml, followed by loading the samples in a linear sucrose gradient (10 to 50%) prepared with a Gradient Master former (BioComp Instruments). The samples were then centrifuged for 2 hours at 260,000*g* at 4°C in a SW41Ti rotor (Beckman). The gradients were analyzed by a Piston Gradient Fractionator [BioComp Instruments; attached to the Model EM-1 Econo UV monitor (Bio-Rad)] for continuous measurement at an absorbance wavelength of 254 nm. Flowing liquid was collected sequentially every 1 ml, followed by quantitative real-time PCR to detect TAF9B mRNA levels within each fraction.

### T cell transfer model of colitis

Naïve CD4^+^CD45RB^hi^ cells were isolated by flow cytometry and were injected intraperitoneally into CB17-SCID recipients (1 × 10^6^ cells per mouse). DC@CFO/BFO nanoparticles (10 mg/kg) were intravenously injected into mice at indicated time points, and 1.5-mT magnetic field stimulation was applied for 30 min every day after nanoparticles injection. Body weight change of each mouse was monitored weekly after transfer. Mice were euthanized when significant weight loss occurred in the experimental groups (*69*).

### CIA model

DBA/1 mice were injected intradermally with 100 μg of bovine CII emulsified in Freund’s complete adjuvant containing heat-killed mycobacterium (4 mg/ml). On day 21, a booster emulsion prepared with CII and Freund’s incomplete adjuvant was administered intradermally near the primary injection site. DC@CFO/BFO nanoparticles (10 mg/kg) were intravenously injected into mice at indicated time points, and 1.5-mT magnetic field was loaded for 30 min every day after nanoparticle injection. Arthritic animals were clinically assessed every other day after disease onset. The clinical scores were assigned to evaluate disease as follows: 0 = no signs of arthritis; 1 = swelling and/or redness of the paw or one digit; 2 = two joints involved; 3 = more than two joints involved; and 4 = severe arthritis of the entire paw and digits. Each limb was graded, resulting in a maximal clinical score of 16 per animal.

### Enzyme-linked immunosorbent assay

Enzyme-linked immunosorbent assay for glycoprotein on cell membrane or nanoparticles was performed according to the manufacturer’s instructions (ELISA LAB, JYM0538Mo).

Anti-CII–specific antibodies in serum collected from immunized mice were measured by enzyme-linked immunosorbent assay (ELISA). Serum samples were added in plates precoated with CII (10 μg/ml), followed by goat antibody to mouse IgM, IgG1, IgG2a, IgG2b, IgG3, and IgA (Southern Biotech, SBA Clonotyping System-HRP Kit, 5300-01).

### Serum inflammatory cytokine detection

Murine serum samples were collected from CIA mice and inflammatory cytokines were measured with the Proteome Profiler Mouse Cytokine Array Kit (R&D Systems, ARY006) according to the manufacturer’s instructions (*70*).

### T cell cytokine profile analysis

CD4^+^ T cells were isolated and treated with magnetoelectric nanocomposites and magnetic field stimulation under T_H_1- or T_H_2-polarizing condition. Cell culture medium was collected, and multiplexed detection of indicated cytokines was performed using the MILLIPLEX Kit (MHSTCMAG-70KPMX, Merck Millipore, Germany) according to the manufacturer’s instructions on the FLEXMAP 3D (Merck Millipore, Germany) instrument.

### Histological analysis

For histological evaluation of mouse arthritis, mouse hind paws and femur were fixed with 4% (w/v) paraformaldehyde, decalcified in EDTA solution, dehydrated in a graded series of alcohol, paraffin embedded, and tissue sectioned to 5 μm for histological evaluation. H&E staining, safranin-O/fast green, and toluidine blue staining were performed for scoring the histopathology of inflammatory arthritis. For H&E staining of mice colon, the tissues were fixed with formaldehyde and then embedded in paraffin. Sections of 5-μm thickness were used for H&E staining with a standard protocol. Images were acquired using an Olympus IX51 microscope.

### RNA sequencing

To study the role of magnetoelectric nanocomposites in T cell–mediated response, CD4^+^ T cells were activated with anti-CD3/CD28 antibodies, incubated with magnetoelectric nanocomposites, and subjected to magnetic field treatment. To investigate the role of magnetoelectric nanocomposites in T_H_2 cell–mediated response, CD4^+^ T cells were incubated with magnetoelectric nanocomposites and subjected to magnetic field treatment under T_H_2-skewing condition. To envaluate the role of TAF9B in DC@CFO/BFO nanoparticle–mediated T cell immunity, WT and *Taf9b*^─/─^ naïve CD4^+^ T cells were incubated with magnetoelectric nanocomposites and subjected to magnetic field treatment under T_H_2-skewing condition. Total RNA was then purified using poly-T oligo-attached magnetic beads. RNA-seq libraries were constructed using a NEBNext Ultra RNA 24 Library Prep Kit for Illumina (NEB, USA) and sequenced on an Illumina platform with 125/150–base pair paired-end reads. According to the manufacturer’s instructions, clean data were obtained by removing reads containing adapter and poly-N as well as low-quality reads from raw data. Clean reads were mapped with the reference genome Hisat2 (version 2.0.5) based on the gene model annotation file. Fragments per kilobase per million mapped reads of each gene was calculated on the basis of the length of the gene and read count mapped to this gene. Gene sets from RNA-seq data were analyzed for overlap with curated datasets (C5.all.V6.2, H.all.V6.2) in the MSigDB using the web interface available at http://software.broadinstitute.org/gsea/index.jsp.

### ATAC sequencing

WT or *Taf9b*^─/─^ naïve CD4^+^ T cells were incubated with magnetoelectric nanocomposites and subjected to magnetic field treatment under T_H_2-skewing condition. The unfixed nuclei of these cells were tagged with tn5 transposase using TruePrep DNA Library Prep Kit V2 for Illumina (Vazyme, TD501), and the resulting library fragments were generated by 16 PCR cycles and sequenced on an Illumina NovaSeq 6000 instrument. After adapters were removed, the reads were aligned to mouse reference genome (mm10) and were annotated to the nearest gene TSSs using esATAC. All duplicated reads, mitochondrial reads, and low-quality reads were removed. The visualization of peak distribution along genomic regions of genes of interest was performed with the Integrative Genomics Viewer.

### Quantitative proteomics analysis

CD4^+^ T cells were isolated and then incubated with magnetoelectric nanocomposites and magnetic field treatment under T_H_2-skewing condition. A 100 μg of protein sample from each subject was subjected to liquid chromatography tandem mass spectrometry (LC-MS/MS) analysis by timsTOF Pro2.

The surface protein of DC@CFO/BFO nanoparticles and total protein of DCs were collected, and 100 μg of protein sample from each subject was performed LC-MS/MS analysis by timsTOF Pro2.

### Untargeted metabolomics analysis

CD4^+^ T cells were isolated and then incubated with magnetoelectric nanocomposites and magnetic field treatment under T_h_2-skewing condition. To extract metabolites of T cells, the cell pellet was resuspended with 500 μl of methanol (prechilled to −80°C) and frozen in liquid nitrogen. These quenched cells were then thawed and vortexed for 30 s. After centrifugation at 800*g* at 4°C for 1 min, the supernatant was transferred to a fresh tube and lyophilized under vacuum. The dried samples were then reconstituted in 80% (v/v) methanol and incubated at 4°C for 15 min. The samples were then centrifuged at 12,000*g* at 4°C for 20 min, and the supernatant was used for LC-MS/MS analysis.

### Statistical analysis

Statistical analysis was performed using the Prism GraphPad software v7.0. Differences between the two groups were analyzed using a Mann-Whitney *U* test or two-tailed Student’s *t* test. *P* < 0.05 was considered significant.
