# Supplementary material for: Magnetoelectric nanoparticles drive TAF9B+ TH2 cell expansion to alleviate inflammation
Source: Sci Adv. 2026 Feb 11;12(7):eadz3199. doi: 10.1126/sciadv.adz3199 (PMC12893300; doi:10.1126/sciadv.adz3199)
Supplement: Supplementary file 1 — Figs. S1 to S21 [file sciadv.adz3199_sm.pdf]

Science Advances
AAAS

Supplementary Materials for

Magnetoelectric nanoparticles drive TAF9B$^+$ T_H2 cell expansion to alleviate inflammation

Jia Song *et al.*

Corresponding author: Yaojin Wang, yjwang@njust.edu.cn; Dan Lu, taotao@bjmu.edu.cn; Xuehui Zhang, zhangxuehui@bjmu.edu.cn; Xuliang Deng, kqdengxuliang@bjmu.edu.cn

Sci. Adv. **12**, eadz3199 (2026)
DOI: 10.1126/sciadv.adz3199

This PDF file includes:

Figs. S1 to S21

Fig. S1

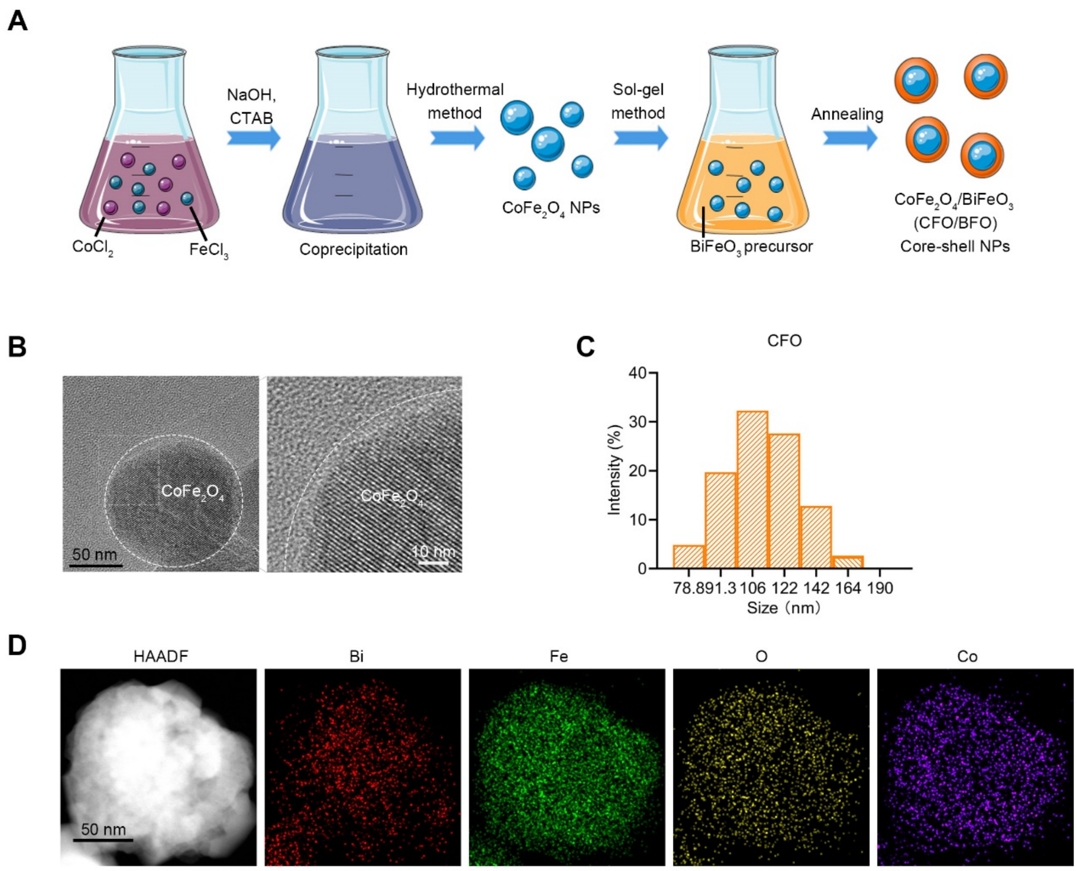

Fig. S1. Fabrication and characterization of CFO/BFO nanocomposites.

(A) Graphic illustration of the fabrication of CFO/BFO nanocomposites. Core layer structure CFO nanoparticles were fabricated by the hydrothermal method, and core-shell CFO/BFO nanoparticles were fabricated by the sol-gel method.

(B) Representative high-resolution TEM images of CFO nanoparticles showing uniform spherical morphology and visible lattice fringes.

(C) Hydrodynamic size distribution of CFO nanoparticles (0.1 mg/mL) measured using a Zetasizer Nano-ZS Instrument.

(D) High Angle Annular Dark Field (HAADF) and the corresponding energy-dispersive spectroscopy (EDS) elemental mapping images of CFO/BFO nanoparticles.

Fig. S2

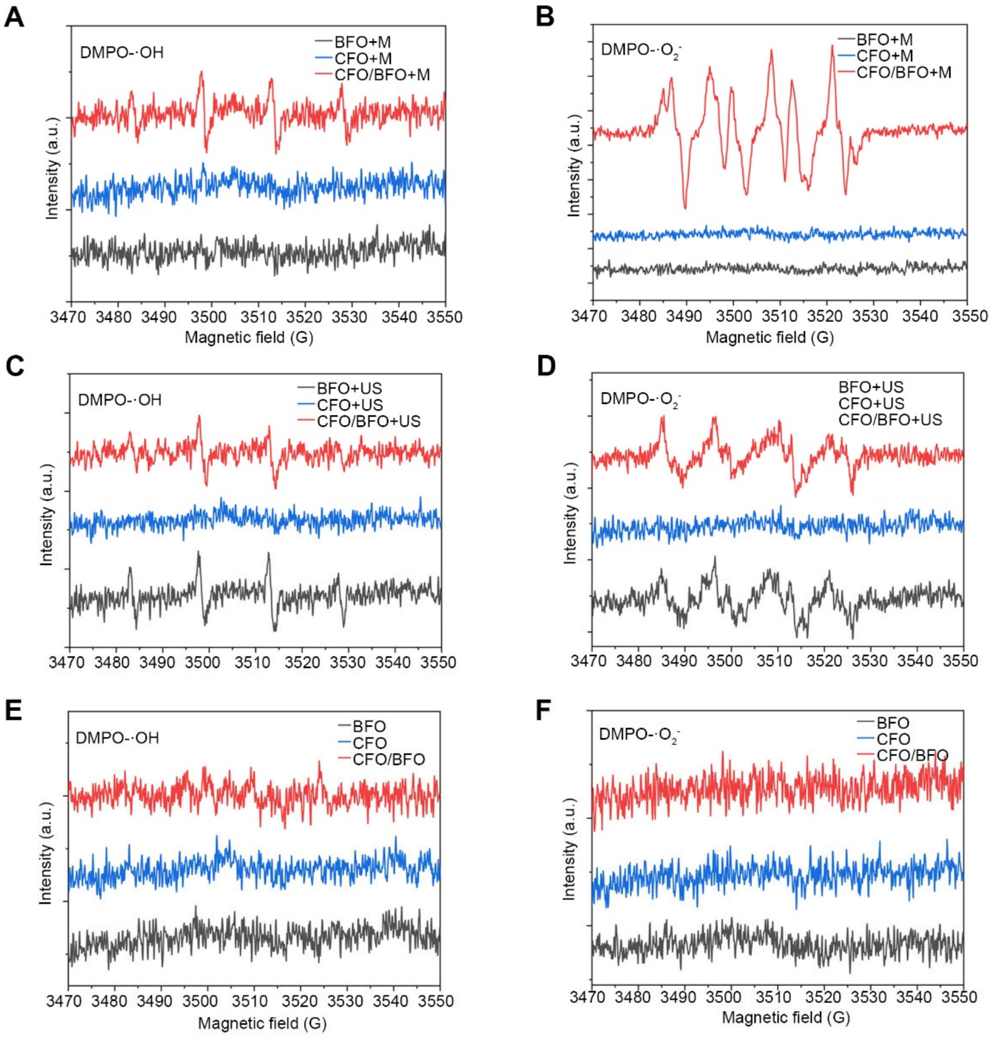

Fig. S2. Detection of Oxygen free radical by Electron Paramagnetic Resonance (EPR) spectroscopy.

(A, B) Electron Paramagnetic Resonance (EPR) of radical ·OH (g) and·O_2^- (h) created by CFO/BFO nanocomposites under 1.5 mT magnetic field loading.

(C, D) Electron Paramagnetic Resonance (EPR) of radical ·OH (B) and ·O_2^- (C) created by CFO/BFO nanocomposites under ultrasound.

(E, F) Electron Paramagnetic Resonance (EPR) of radical $\cdot OH$ (B) and $\cdot O_2^-$ (C) created by CFO/BFO nanocomposites.

Fig. S3

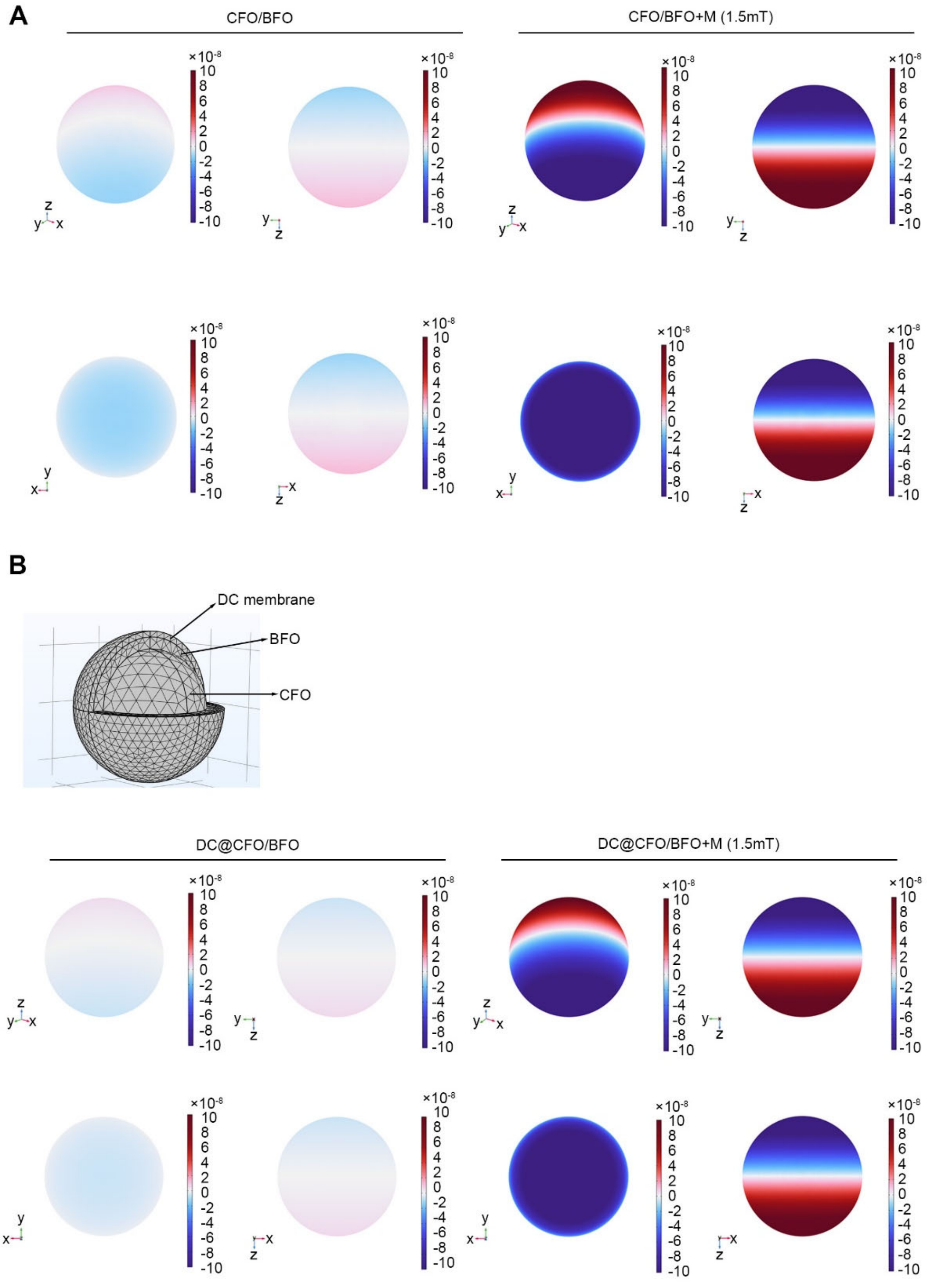

Fig. S3. Finite element analysis (FEA) of surface potential distribution of CFO/BFO and DC@CFO/BFO nanocomposites.

(A) The surface potential of CFO/BFO nanocomposites with or without magnetic field (1.5 mT) assessed by COMSOL simulations.

(B) The surface potential of DC@CFO/BFO nanocomposites with or without magnetic field (1.5 mT) assessed by COMSOL simulations.

Fig. S4

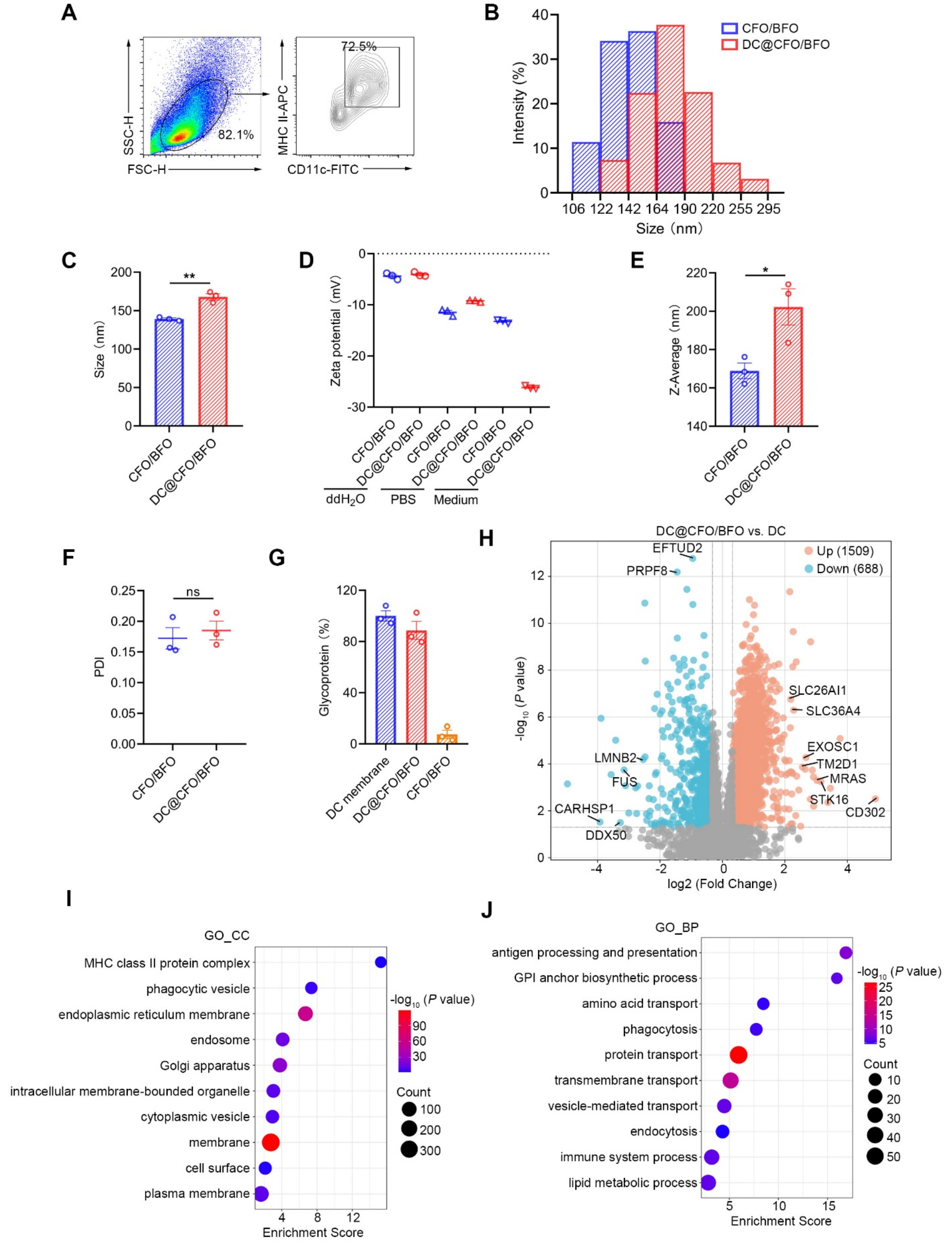
A
SSC-H
FSC-H
82.1%
MHC II-APC
CD11c-FITC
72.5%
B
Intensity (%)
Size（nm）
CFO/BFO
DC@CFO/BFO
C
Size（nm）
**
D
Zeta potential（mV）
ddH2O
PBS
Medium
E
Z-Average（nm）
*
F
PDI
ns
G
Glycoprotein（%）
DC membrane
H
DC@CFO/BFO vs. DC
Up (1509)
Down (688)
EFTUD2
PRPF8
SLC26AI1
SLC36A4
EXOSC1
TM2D1
MRAS
STK16
CD302
LMNB2
FUS
CARHSP1
DDX50
-log10 (P value)
log2 (Fold Change)
I
GO_CC
MHC class II protein complex
phagocytic vesicle
endoplasmic reticulum membrane
endosome
Golgi apparatus
intracellular membrane-bounded organelle
cytoplasmic vesicle
membrane
cell surface
plasma membrane
Enrichment Score
Count
J
GO_BP
antigen processing and presentation
GPI anchor biosynthetic process
amino acid transport
phagocytosis
protein transport
transmembrane transport
vesicle-mediated transport
endocytosis
immune system process
lipid metabolic process
Enrichment Score
Count

Fig. S4. Fabrication and characterization of DC@CFO/BFO nanocomposites.

(A) Flow cytometric analysis of surface markers of bone marrow derived dendritic cells (BMDCs).

(B, C) The particle sizes of CFO/BFO nanocomposites and DC@CFO/BFO nanocomposites with concentration of 0.1 mg/ml measured by the Zeta Sizer Nano-ZS Instrument (n = 3, mean ± s.e.m., ***P* = 0.0026, (Shapiro-Wilk test $P > 0.1$, F test $P > 0.1$) two-tailed unpaired Student's t-test).

(D) The zeta potentials of CFO/BFO nanocomposites and DC@CFO/BFO nanocomposites with concentration of 0.1 mg/ml in different solutions (n = 3, mean ± s.e.m.).

(E, F) The z-average sizes and Polydispersity Index (PDI) of CFO/BFO nanocomposites and DC@CFO/BFO nanocomposites with concentration of 0.1 mg/ml measured by the Zeta Sizer Nano-ZS Instrument (n = 3, mean ± s.e.m., ns, not significant ($P > 0.05$), **P* = 0.0319, (Shapiro-Wilk test $P > 0.1$, F test $P > 0.1$) two-tailed unpaired Student's t-test).

(G) The glycoprotein levels on DC membrane, CFO/BFO nanocomposites and DC@CFO/BFO nanocomposites were measured by ELISA (n = 3, mean ± s.e.m.).

(**H**) Volcano plot analysis of protein expression between DC@CFO/BFO surface and DC. Red, proteins upregulated in DC@CFO/BFO. Blue, proteins downregulated in DC@CFO/BFO.

(**I**, **J**) Proteins that were significantly enriched in DC@CFO/BFO surface, compared with DC cells, were analyzed using DAVID with GO terms.

Fig. S5

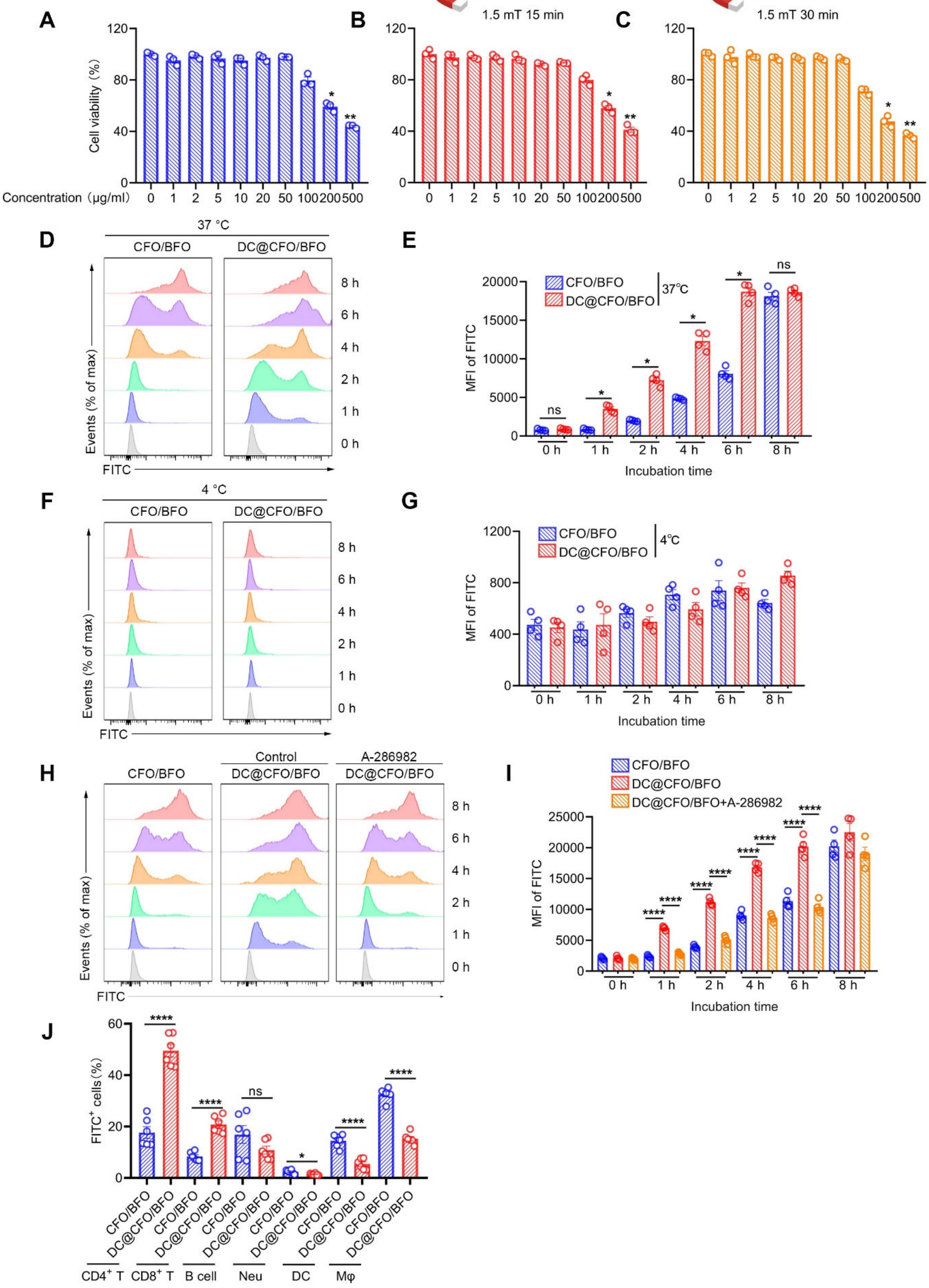
A
B
C
1.5 mT 15 min
1.5 mT 30 min
Cell viability (%)
Concentration (μg/ml)
0 1 2 5 10 20 50 100 200 500
D
37 °C
CFO/BFO
DC@CFO/BFO
Events (% of max)
FITC
8 h
6 h
4 h
2 h
1 h
0 h
E
CFO/BFO
DC@CFO/BFO
37°C
MFI of FITC
Incubation time
F
4 °C
G
4°C
H
Control
A-286982
I
DC@CFO/BFO+A-286982
J
FITC+ cells (%)
CD4+ T
CD8+ T
B cell
Neu
DC
Mφ

Fig. S5. Biosafety evaluation of the DC@CFO/BFO nanoparticles.

(A-C) Naïve $CD4^+$ T cells were isolated and incubated with DC@CFO/BFO nanoparticles for 24 hours with or without magnetic field. The viability of T cells was measured by CCK-8 assay at an absorbance wavelength of 450 nm (n = 3, mean ± s.e.m., **P* < 0.05, ***P* < 0.01, Kruskal-Wallis Test).

(D-G) FITC-conjugated CFO/BFO nanoparticles or DC@CFO/BFO nanocomposites (10 μg/mL) were incubated with murine naïve $CD4^+$ T cells for indicated time durations at 37 °C or 4 °C, and the $FITC^+$ cells were detected by flow cytometry. MFI, Mean Fluorescence Intensity (n = 4, mean ± s.e.m., ns, not significant (*P* > 0.05), **P* < 0.05, Mann-Whitney U test).

(H, I) FITC-conjugated CFO/BFO nanoparticles or DC@CFO/BFO nanocomposites (10 μg/mL) were incubated with murine naïve $CD4^+$ T cells for indicated time durations with or without A-286982 treatment, and the $FITC^+$ cells were detected by flow cytometry. MFI, Mean Fluorescence Intensity (n = 4, mean ± s.e.m., *****P* < 0.0001, (Shapiro-Wilk test, *P* > 0.1; Brown-Forsythe test, *P* > 0.1) one-way ANOVA).

(**J**) FITC-conjugated CFO/BFO nanoparticles or DC@CFO/BFO nanocomposites (10 μg/mL) were intravenously injected into mice, and splenocytes were harvested 24 hours after injection. $FITC^+$ cells were detected by flow cytometry (n = 6, mean ± s.e.m., ns, not significant (*P* > 0.05), **P* = 0.0156, *****P* < 0.0001, (Shapiro-Wilk test *P* > 0.1, F test *P* > 0.1) two-tailed unpaired Student's t-test).

Fig. S6

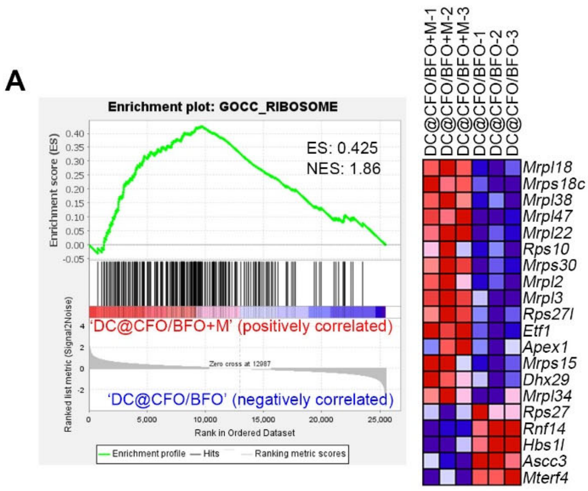

Fig. S6. Transcriptional analysis of T cell with DC@CFO/BFO nanocomposites treatment and magnetic field stimulation.

(A) $CD4^+$ T cells were activated with antibodies against CD3/CD28, treated with DC@CFO/BFO nanocomposites and stimulated with an external magnetic field. GSEA of genes expressed in $CD4^+$ T cells treated by DC@CFO/BFO nanocomposites in the presence or absence of magnetic field stimulation (n = 3 biological replicates). ES, enrichment score; NES, normalized enrichment score.

Fig. S7

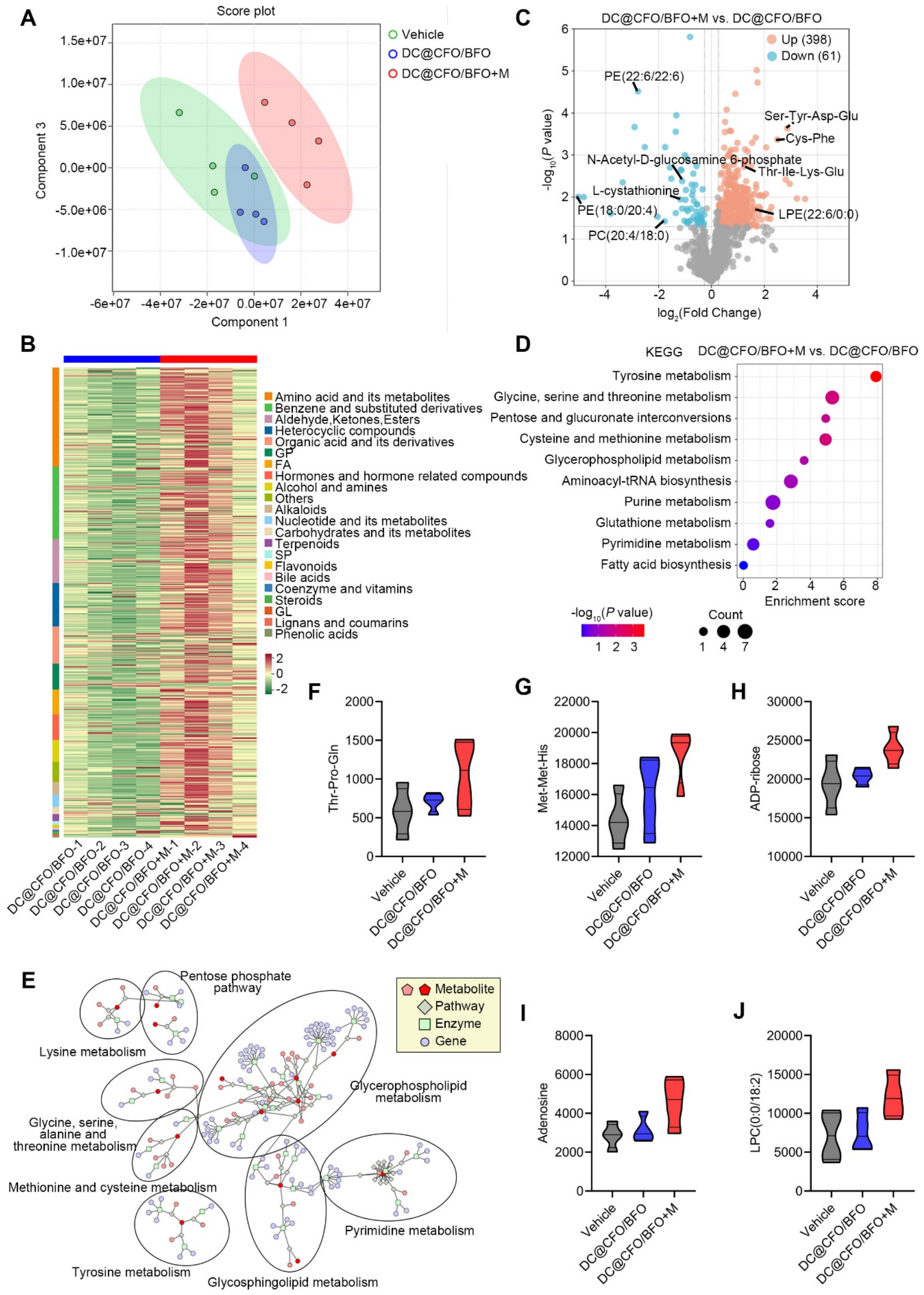
A
Score plot
Vehicle
DC@CFO/BFO
DC@CFO/BFO+M
Component 3
Component 1
C
DC@CFO/BFO+M vs. DC@CFO/BFO
Up (398)
Down (61)
PE(22:6/22:6)
Ser-Tyr-Asp-Glu
Cys-Phe
N-Acetyl-D-glucosamine 6-phosphate
Thr-Ile-Lys-Glu
L-cystathionine
PE(18:0/20:4)
LPE(22:6/0:0)
PC(20:4/18:0)
-log10(P value)
log2(Fold Change)
B
Amino acid and its metabolites
Benzene and substituted derivatives
Aldehyde,Ketones,Esters
Heterocyclic compounds
Organic acid and its derivatives
GP
FA
Hormones and hormone related compounds
Alcohol and amines
Others
Alkaloids
Nucleotide and its metabolites
Carbohydrates and its metabolites
Terpenoids
SP
Flavonoids
Bile acids
Coenzyme and vitamins
Steroids
GL
Lignans and coumarins
Phenolic acids
DC@CFO/BFO-1
DC@CFO/BFO-2
DC@CFO/BFO-3
DC@CFO/BFO-4
DC@CFO/BFO+M-1
DC@CFO/BFO+M-2
DC@CFO/BFO+M-3
DC@CFO/BFO+M-4
D
KEGG DC@CFO/BFO+M vs. DC@CFO/BFO
Tyrosine metabolism
Glycine, serine and threonine metabolism
Pentose and glucuronate interconversions
Cysteine and methionine metabolism
Glycerophospholipid metabolism
Aminoacyl-tRNA biosynthesis
Purine metabolism
Glutathione metabolism
Pyrimidine metabolism
Fatty acid biosynthesis
Enrichment score
-log10(P value)
Count
F
Thr-Pro-Gln
G
Met-Met-His
H
ADP-ribose
Vehicle
DC@CFO/BFO
DC@CFO/BFO+M
E
Pentose phosphate pathway
Lysine metabolism
Glycine, serine, alanine and threonine metabolism
Glycerophospholipid metabolism
Methionine and cysteine metabolism
Pyrimidine metabolism
Tyrosine metabolism
Glycosphingolipid metabolism
Metabolite
Pathway
Enzyme
Gene
I
Adenosine
J
LPC(0:0/18:2)

Fig. S7. DC@CFO/BFO nanoparticles promote T cell amino acid metabolic fitness.

(A) PCA analysis of metabolites of $CD4^+$ T cells in different treatment groups.

(B) Heatmap of metabolites of $CD4^+$ T cells in different treatment groups was shown (n = 4 biological replicates).

(C) Volcano plot analysis of pairwise comparison of metabolites between the DC@CFO/BFO+M group and DC@CFO/BFO group.

(D) Enrichment analysis of metabolites upregulated in the DC@CFO/BFO+M group, as compared with the DC@CFO/BFO group.

(E) Interaction of metabolic pathways upregulated in the DC@CFO/BFO+M group, as compared with the DC@CFO/BFO group.

(F-J) Representative metabolite levels in $CD4^+$ T cells in different treatment groups (n = 4 biological replicates).

Fig. S8

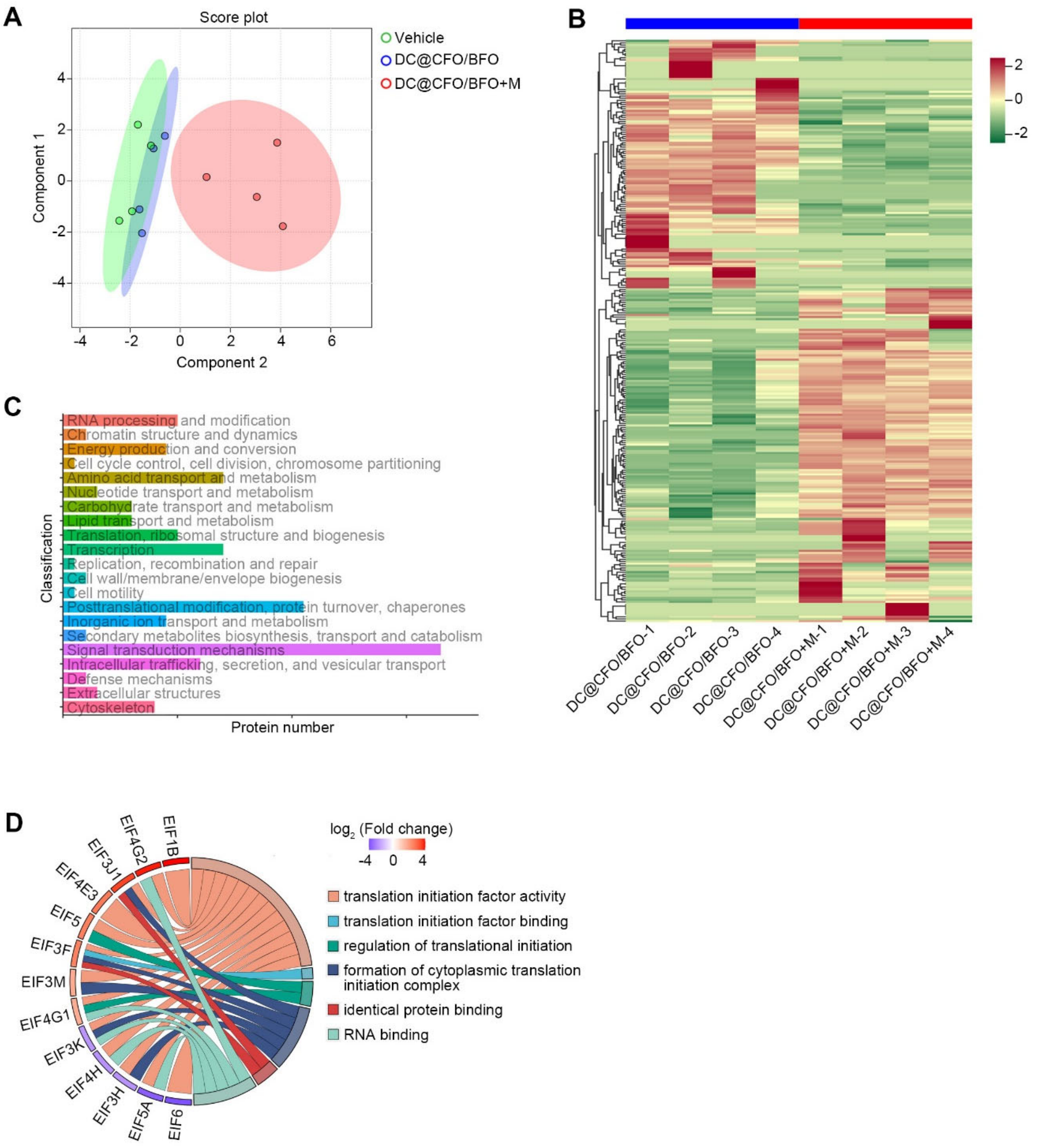

Fig. S8. Proteomic analysis of T cells with DC@CFO/BFO treatment and magnetic field stimulation.

(A) PCA analysis of the proteome of $CD4^+$ T cells in different treatment groups.

(B) Heatmap of the proteome of $CD4^+$ T cells in different treatment groups was presented (n = 4 biological replicates).

(C) Proteins that were significantly upregulated in the DC@CFO/BFO+M group, as compared with the DC@CFO/BFO group, by Cluster of Orthologous Groups (COG) analysis.

(D) Differentially expressed proteins (DEPs) between DC@CFO/BFO+M group and DC@CFO/BFO group were analyzed using DAVID with GO terms.

Fig. S9

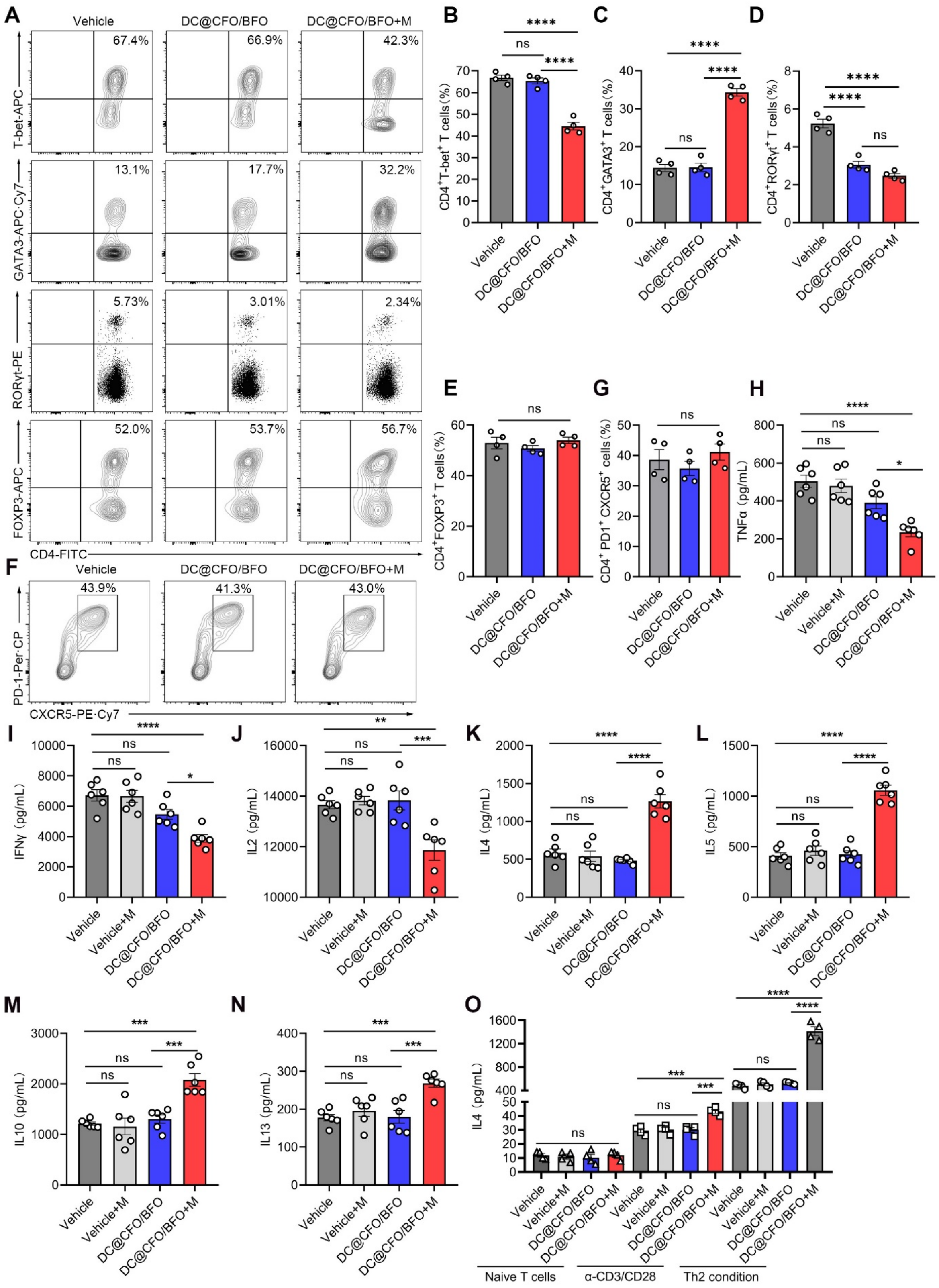
A
Vehicle
DC@CFO/BFO
DC@CFO/BFO+M
67.4%
66.9%
42.3%
13.1%
17.7%
32.2%
5.73%
3.01%
2.34%
52.0%
53.7%
56.7%
T-bet-APC
GATA3-APC·Cy7
RORγt-PE
FOXP3-APC
CD4-FITC
B
C
D
E
G
H
F
43.9%
41.3%
43.0%
PD-1-Per-CP
CXCR5-PE·Cy7
I
J
K
L
M
N
O
Naive T cells
α-CD3/CD28
Th2 condition
Vehicle
Vehicle+M
TNFα (pg/mL)
IFNγ (pg/mL)
IL2 (pg/mL)
IL4 (pg/mL)
IL5 (pg/mL)
IL10 (pg/mL)
IL13 (pg/mL)

Fig. S9. DC@CFO/BFO nanocomposites promote Th2 cell polarization under external magnetic field stimulation.

(A-E) Flow cytometric analysis of the staining of T-bet, GATA3, RORγt and FOXP3 (assessing differentiation efficiency) in naïve T cells ($CD4^+CD25^-CD62L^{hi}CD44^{lo}$) activated with plate-bound anti-CD3 plus anti-CD28 and cultured under various polarizing condition with or without DC@CFO/BFO nanocomposites and magnetic field treatment (n = 4, mean ± s.e.m., ns, not significant ($P > 0.05$), ****$P < 0.0001$, (Shapiro-Wilk test, $P > 0.1$; Brown-Forsythe test, $P > 0.1$) one-way ANOVA).

(F, G) Flow cytometric analysis of the staining of PD1 and CXCR5 (assessing differentiation efficiency) in naïve T cells ($CD4^+CD25^-CD62L^{hi}CD44^{lo}$) activated with plate-bound anti-CD3 plus anti-CD28 and cultured under Tfh polarizing condition with or without DC@CFO/BFO nanocomposites and magnetic field treatment (n = 4, mean ± s.e.m., ns, not significant ($P > 0.05$), (Shapiro-Wilk test, $P > 0.1$; Brown-Forsythe test, $P > 0.1$) one-way ANOVA).

(H-J) $CD4^+$ T cells were activated with antibodies against CD3/CD28 and were induced to differentiate into Th1 cells. These cells were then treated with DC@CFO/BFO nanocomposites in presence or absence of magnetic field stimulation. Cell culture medium was collected, and multiplexed detection of indicated cytokines was performed (n = 6, mean ± s.e.m., ns, not significant ($P > 0.05$), *$P < 0.05$, **$P = 0.0023$, ***$P = 0.0009$, ****$P < 0.0001$, (Shapiro-Wilk test, $P > 0.1$; Brown-Forsythe test, $P > 0.1$) one-way ANOVA).

(K-N) $CD4^+$ T cells were activated with antibodies against CD3/CD28 and were induced to differentiate into Th2 cells. These cells were then treated with DC@CFO/BFO nanocomposites in presence or absence of magnetic field stimulation. Cell culture medium was collected, and multiplexed detection of indicated cytokines was performed (n = 6, mean ± s.e.m., ns, not

significant ($P > 0.05$), **$P < 0.01$, ***$P < 0.001$, ****$P < 0.0001$, (Shapiro-Wilk test, $P > 0.1$; Brown-Forsythe test, $P > 0.1$) one-way ANOVA).

(O) $CD4^+$ T cells were activated with antibodies against CD3/CD28 or were induced to differentiate into Th2 cells. These cells were then treated with DC@CFO/BFO nanocomposites in presence or absence of magnetic field stimulation. Cell culture medium was collected, and multiplexed detection of indicated cytokines was performed (n = 4, mean ± s.e.m., ns, not significant ($P > 0.05$), ***$P < 0.001$, ****$P < 0.0001$, one-way ANOVA).

Fig. S10

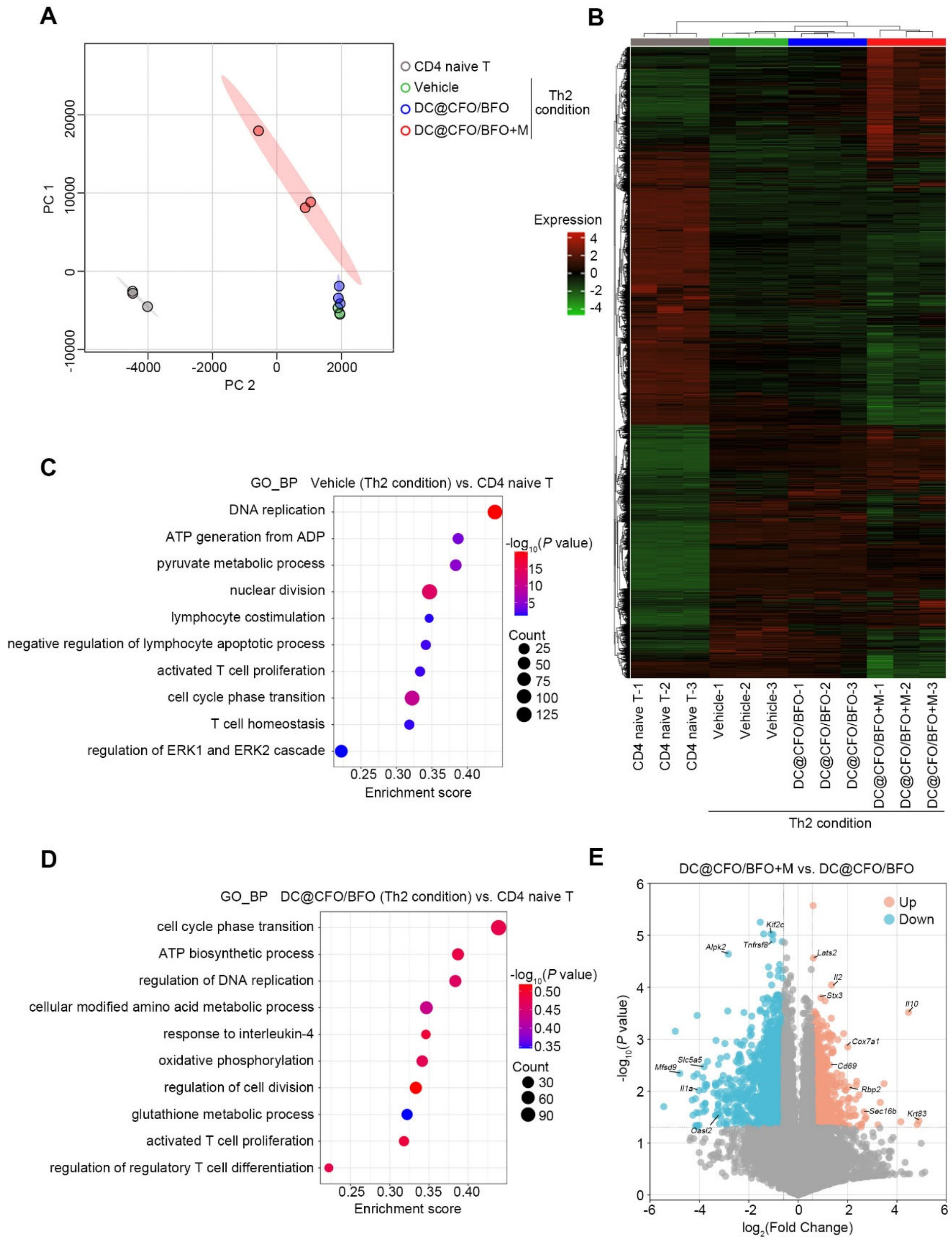
A
CD4 naive T
Vehicle
DC@CFO/BFO
DC@CFO/BFO+M
Th2 condition
PC 1
PC 2
B
Expression
CD4 naive T-1
CD4 naive T-2
CD4 naive T-3
Vehicle-1
Vehicle-2
Vehicle-3
DC@CFO/BFO-1
DC@CFO/BFO-2
DC@CFO/BFO-3
DC@CFO/BFO+M-1
DC@CFO/BFO+M-2
DC@CFO/BFO+M-3
Th2 condition
C
GO_BP Vehicle (Th2 condition) vs. CD4 naive T
DNA replication
ATP generation from ADP
pyruvate metabolic process
nuclear division
lymphocyte costimulation
negative regulation of lymphocyte apoptotic process
activated T cell proliferation
cell cycle phase transition
T cell homeostasis
regulation of ERK1 and ERK2 cascade
-log10(P value)
Count
Enrichment score
D
GO_BP DC@CFO/BFO (Th2 condition) vs. CD4 naive T
cell cycle phase transition
ATP biosynthetic process
regulation of DNA replication
cellular modified amino acid metabolic process
response to interleukin-4
oxidative phosphorylation
regulation of cell division
glutathione metabolic process
activated T cell proliferation
regulation of regulatory T cell differentiation
-log10(P value)
Count
Enrichment score
E
DC@CFO/BFO+M vs. DC@CFO/BFO
Up
Down
Kif2c
Tnfrsf8
Alpk2
Lats2
Il2
Stx3
Il10
Cox7a1
Cd69
Mfsd9
Slc5a5
Il1a
Rbp2
Sec16b
Krt83
Oasl2
-log10(P value)
log2(Fold Change)

Fig. S10. Transcriptional analysis of T cells with DC@CFO/BFO treatment and magnetic field stimulation.

(A) PCA analysis of transcripts of $CD4^+$ T cells in different treatment groups.

(B) Heatmap of gene expression of $CD4^+$ T cells with different treatment is presented (n = 3 biological replicates).

(C) Genes that were significantly upregulated in the Vehicle (Th2 condition) group, compared with the control group (CD4 naïve T cell), were analyzed using DAVID with GO terms.

(D) Genes that were significantly upregulated in the DC@CFO/BFO (Th2 condition) group, compared with the control group (CD4 naïve T cell), were analyzed using DAVID with GO terms.

(E) Volcano plot analysis of pairwise comparison of transcriptional analysis between DC@CFO/BFO+M group and DC@CFO/BFO group.

Fig. S11

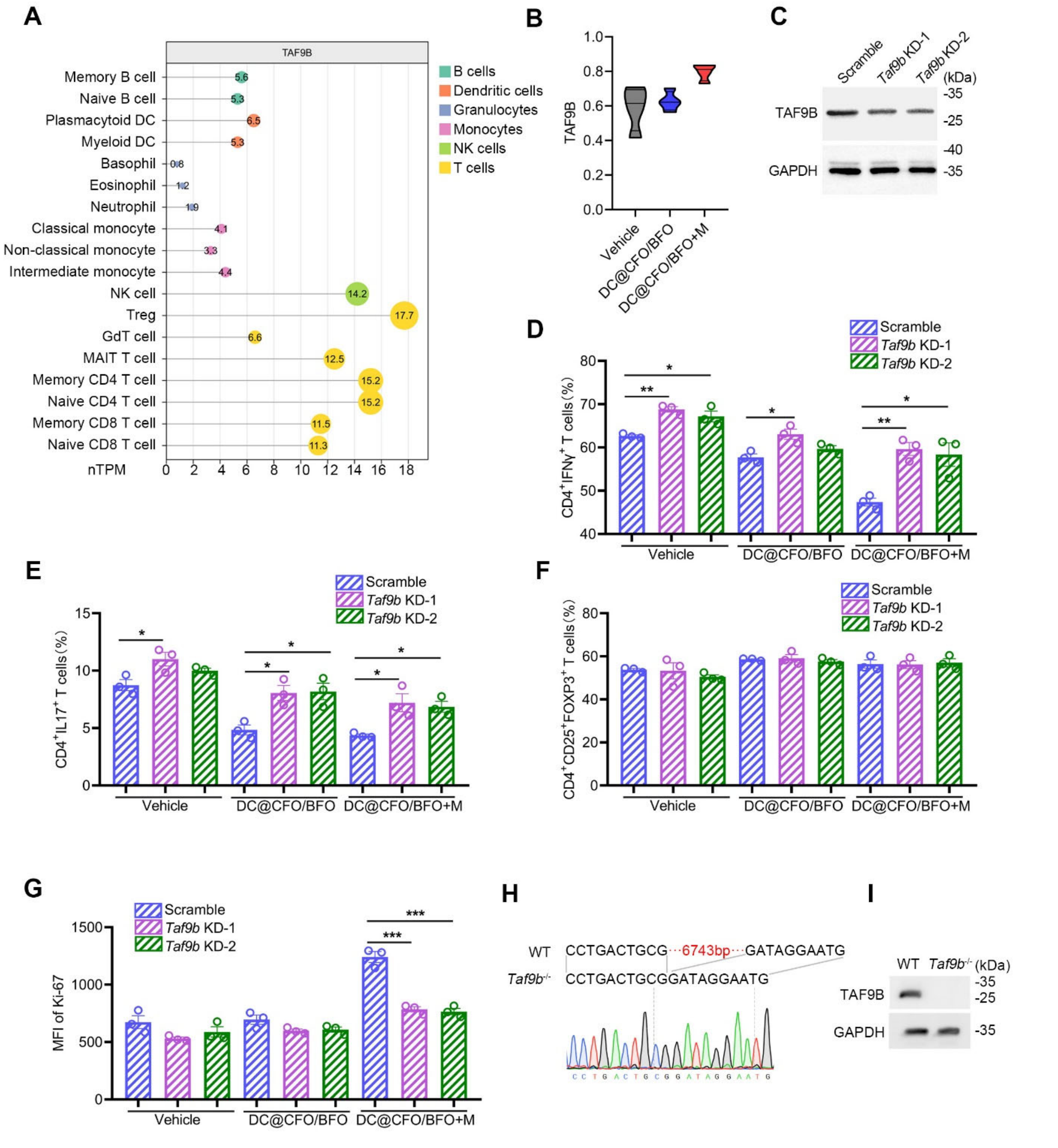

Fig. S11. TAF9B is essential for magnetoelectric nanoparticle-mediated T cell response

(A) TAF9B expression levels in indicated immune cell subsets analyzed by The Human Protein Atlas database.

(B) TAF9B protein levels in $CD4^+$ T cells in different treatment groups under Th2 skewing condition measured by quantitative proteomics analysis (n = 4 biological replicates).

(C) Immunoblot analysis of TAF9B expression in $CD4^+$ T cells infected with retrovirus containing *Taf9b* siRNA.

(D) Naïve $CD4^+$ T cells were activated with plate-bound anti-CD3 plus anti-CD28 for 24 hours and infected with retrovirus containing *Taf9b* siRNA for 24 hours. T cells were then cultured under Th1 polarizing condition with or without DC@CFO/BFO nanocomposites and magnetic field treatment, then re-stimulated for 5 hours with PMA and ionomycin. Proportion of $CD4^+IFN\gamma^+$ cells in different group was analyzed by flow cytometry (n = 3, mean ± s.e.m., *$P < 0.05$, **$P < 0.01$, (Shapiro-Wilk test, $P > 0.1$; Brown-Forsythe test, $P > 0.1$) one-way ANOVA).

(E) Naïve $CD4^+$ T cells were activated with plate-bound anti-CD3 plus anti-CD28 for 24 hours and infected with retrovirus containing *Taf9b* siRNA for 24 hours. T cells were then cultured under Th17 polarizing condition with or without DC@CFO/BFO nanocomposites and magnetic field treatment, followed by re-stimulation for 5 hours with PMA and ionomycin. Proportion of $CD4^+Il17A^+$ cells in different group was analyzed by flow cytometry (n = 3, mean ± s.e.m., *$P < 0.05$, (Shapiro-Wilk test, $P > 0.1$; Brown-Forsythe test, $P > 0.1$) one-way ANOVA).

(F) Naïve $CD4^+$ T cells were activated with plate-bound anti-CD3 plus anti-CD28 for 24 hours and infected with retrovirus containing *Taf9b* siRNA for 24 hours. T cells were then cultured under Treg polarizing condition, with or without treatment with DC@CFO/BFO nanocomposites and magnetic field stimulation. Proportion of iTregs in different group was analyzed by flow cytometry (n = 3, mean ± s.e.m.).

(G) Naïve $CD4^+$ T cells were activated with plate-bound anti-CD3 plus anti-CD28 for 24 hours and infected with retrovirus containing *Taf9b* siRNA for 24 hours. T cells were then cultured under

Th2 polarizing condition with or without DC@CFO/BFO nanocomposites and magnetic field treatment. Expression level of Ki-67 in different group was analyzed by flow cytometry. MFI, Mean Fluorescence Intensity (n = 3, mean ± s.e.m., ***$P < 0.001$, (Shapiro-Wilk test, $P > 0.1$; Brown-Forsythe test, $P > 0.1$) one-way ANOVA).

(**H**) Validation of *Taf9b*$^{-/-}$ mice by Western Blot.

(**I**) Sequencing for genomic DNA of *Taf9b*$^{-/-}$ mice.

Fig. S12

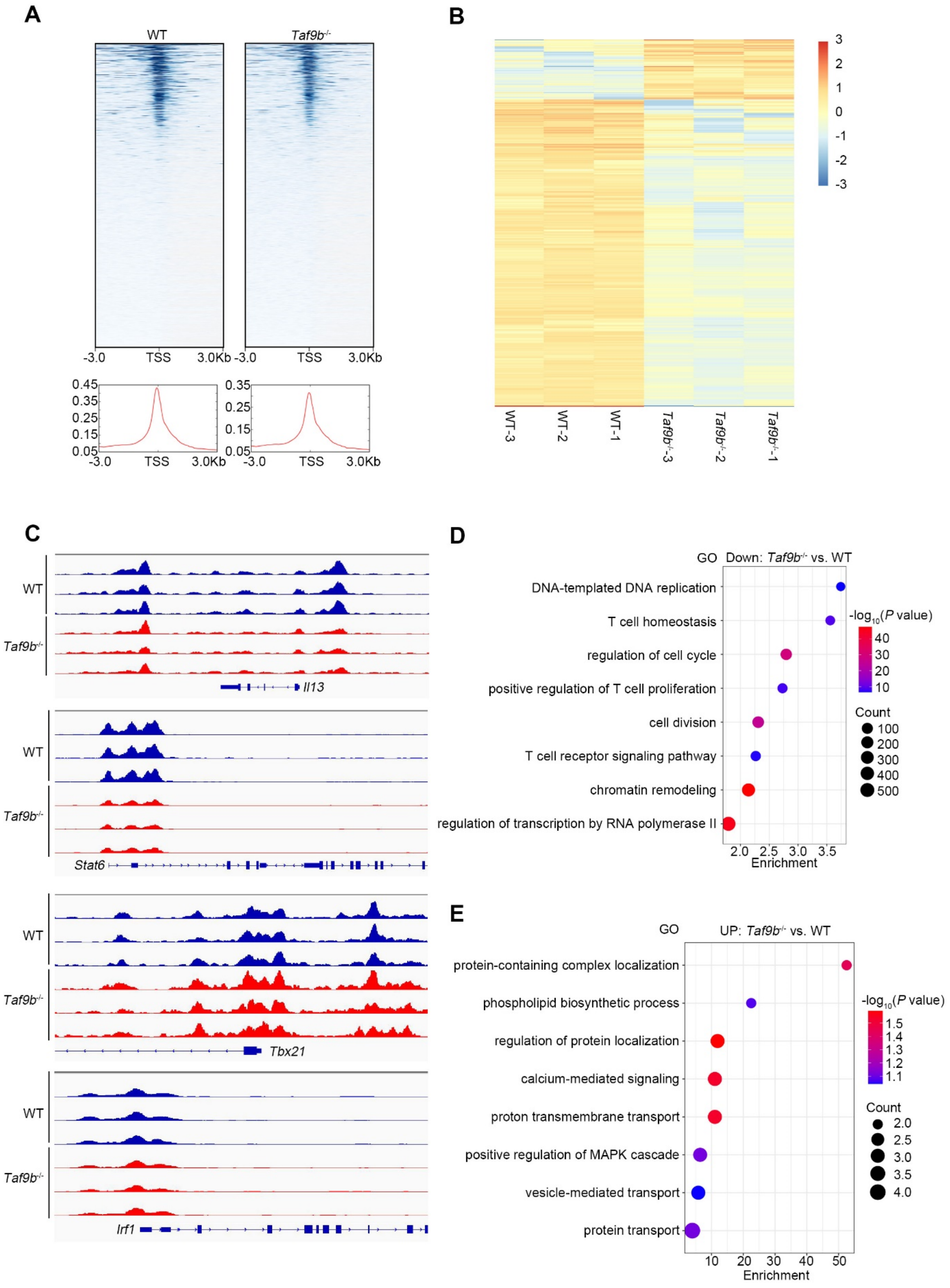
A
WT
Taf9b-/-
-3.0
TSS
3.0Kb
0.45
0.35
0.25
0.15
0.05
B
3
2
1
0
-1
-2
-3
WT-3
WT-2
WT-1
Taf9b-/--3
Taf9b-/--2
Taf9b-/--1
C
WT
Taf9b-/-
Il13
Stat6
Tbx21
Irf1
D
GO Down: Taf9b-/- vs. WT
DNA-templated DNA replication
T cell homeostasis
regulation of cell cycle
positive regulation of T cell proliferation
cell division
T cell receptor signaling pathway
chromatin remodeling
regulation of transcription by RNA polymerase II
-log10(P value)
40
30
20
10
Count
100
200
300
400
500
2.0 2.5 3.0 3.5
Enrichment
E
GO
UP: Taf9b-/- vs. WT
protein-containing complex localization
phospholipid biosynthetic process
regulation of protein localization
calcium-mediated signaling
proton transmembrane transport
positive regulation of MAPK cascade
vesicle-mediated transport
protein transport
-log10(P value)
1.5
1.4
1.3
1.2
1.1
Count
2.0
2.5
3.0
3.5
4.0
10 20 30 40 50
Enrichment

Fig. S12. Chromatin accessibility analysis in *Taf9b* deficient T cells. **(A)** $CD4^+$ WT or *Taf9b*$^{-/-}$ T cells were activated with antibodies against CD3/CD28 and induced to differentiate into Th2 cells, followed by ATAC-seq. Depletion of *Taf9b* impaired the ATAC-seq signal enrichment around the transcription start sites (TSSs).

(B) Heatmap analysis of differential accessibility of gene loci in WT or *Taf9b*$^{-/-}$ T cells.

(**C**) IGV analysis of ATAC-seq coverage peaks at indicated locus in WT and *Taf9b*$^{-/-}$ T cells.

(**D**) ATAC peaks downregulated in the *Taf9b*$^{-/-}$ T cells were analyzed with GO terms.

(**E**) ATAC peaks upregulated in the *Taf9b*$^{-/-}$ T cells were analyzed with GO terms.

Fig. S13

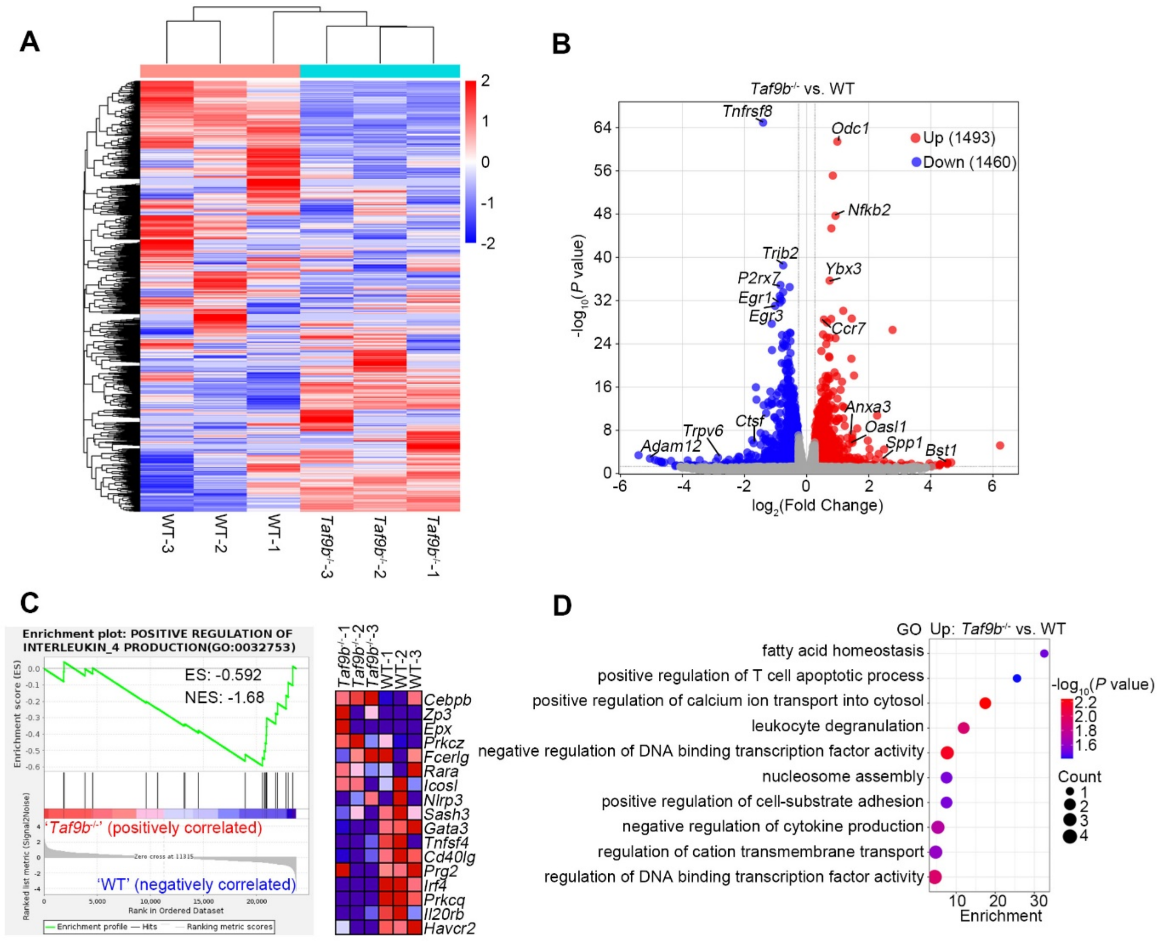

Fig. S13. Transcriptomic analysis of *Taf9b* deficient T cells.

(A) CD4^{+} WT or *Taf9b*$^{-/-}$ T cells were activated with antibodies against CD3/CD28 and induced to differentiate into Th2 cells, followed by RNA-seq. Heatmap analysis of differentially expressed genes in WT or *Taf9b*$^{-/-}$ T cells.

(B) Volcano plot analysis of differentially expressed genes between WT and *Taf9b*$^{-/-}$ T cells.

(C) GSEA of genes expressed in T cells derived from WT and *Taf9b*$^{-/-}$ mice. ES, enrichment score; NES, normalized enrichment score.

(D) Genes that were significantly upregulated in *Taf9b*$^{-/-}$ T cells, compared with WT T cells, were analyzed using DAVID with GO terms.

Fig. S14

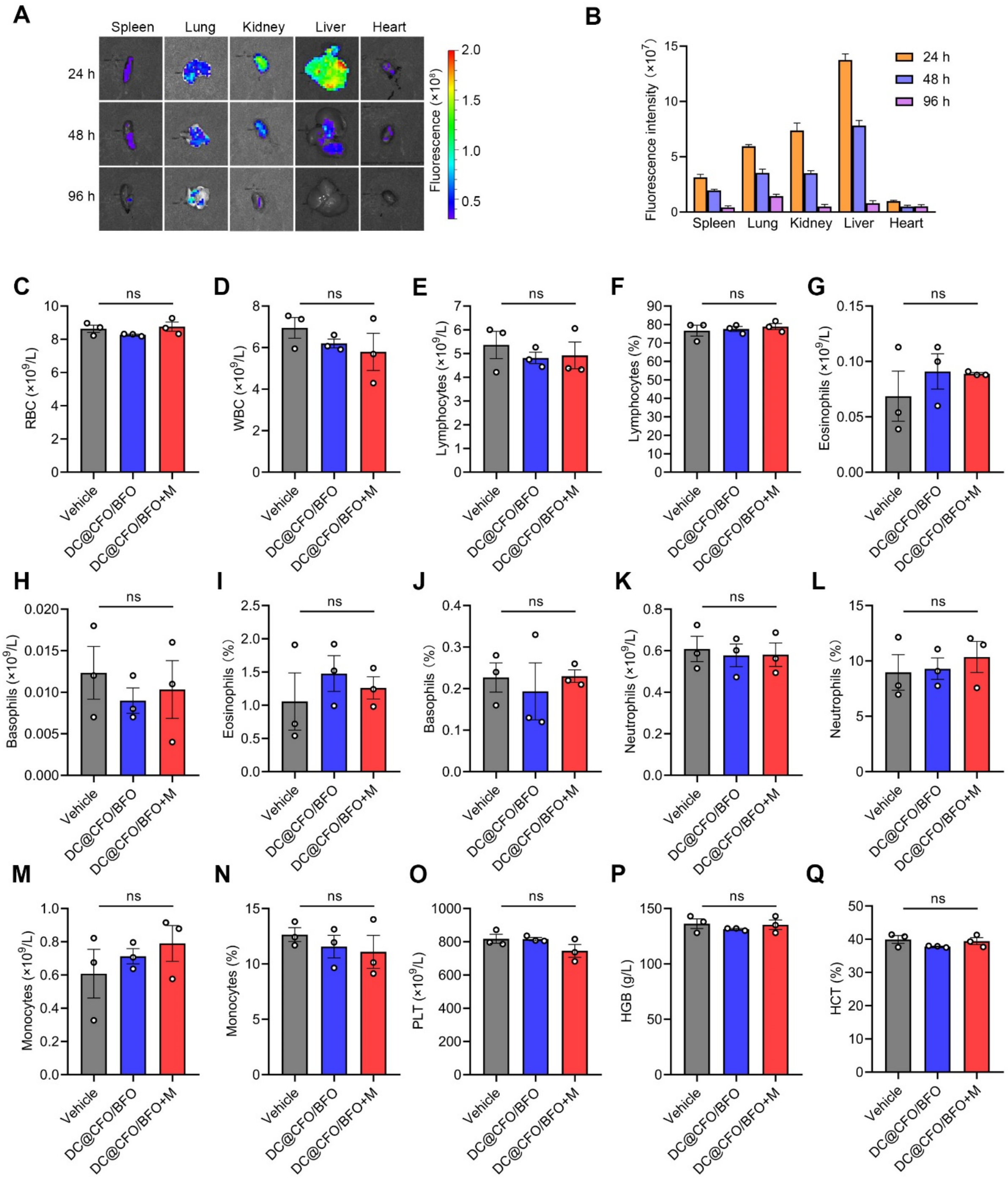

Fig. S14. Biosafety evaluation of DC@CFO/BFO nanoparticles by blood routine tests.

(A, B) FITC-labeled DC@CFO/BFO nanoparticles (10 mg/kg) were intravenously injected into mice and *in vivo* distribution of DC@CFO/BFO was assessed by bioluminescence imaging at indicated timepoints.

(C-Q) C57BL/6 mice were administrated with 50 mg/kg of the DC@CFO/BFO nanocomposites via intravenous injection with magnetic field stimulation (1.5 mT) for 30 minutes every day. After 7 days post injection, mice serum was collected and blood routine test was performed. RBC, Red blood cell; WBC, White blood cell; PLT, platelet; HGB, haemoglobin; MCV, Mean corpuscular volume; HCT, hematocrit (n = 3, mean ± s.e.m., ns, not significant ($P > 0.05$), Kruskal-Wallis Test).

Fig. S15

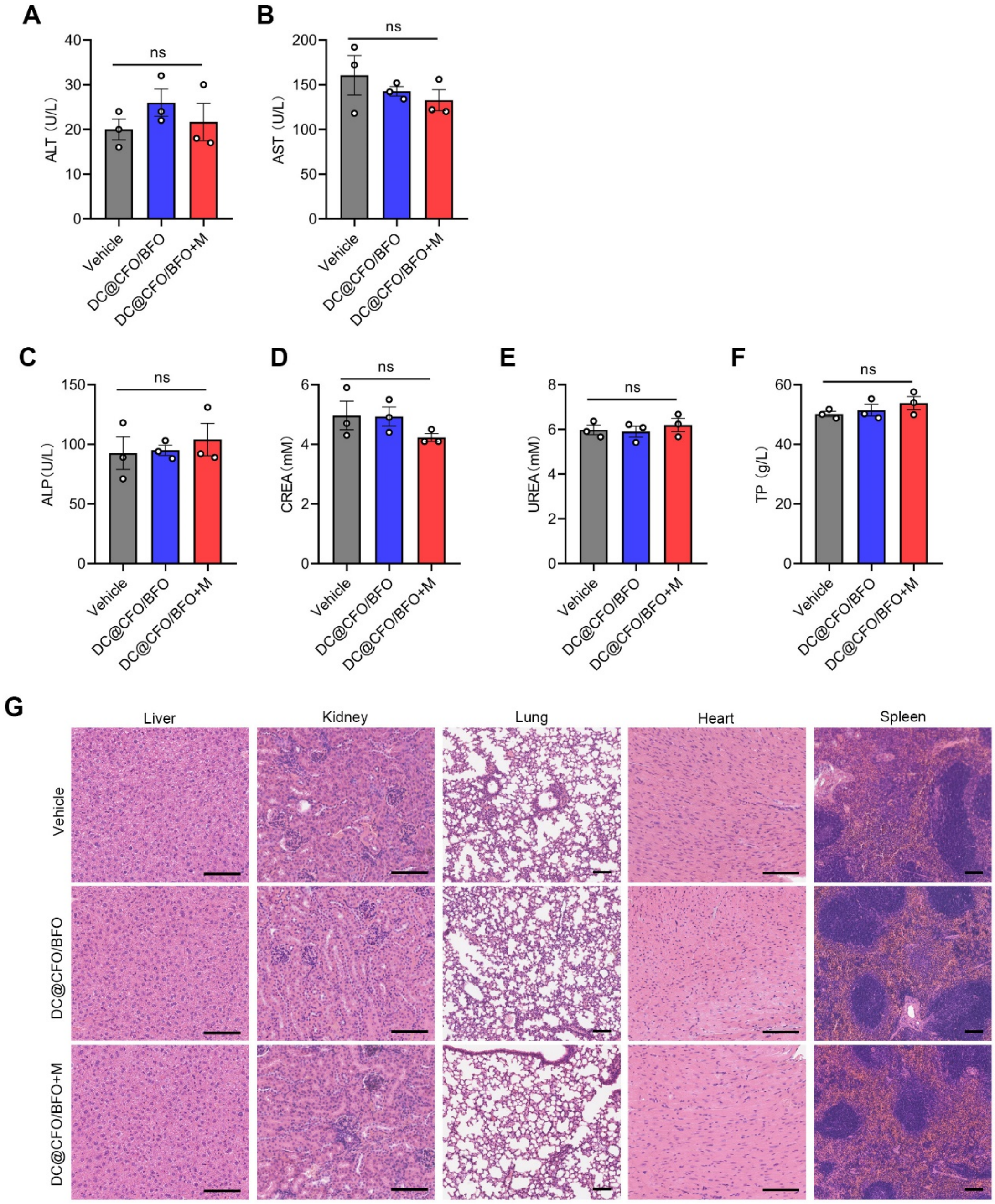

Fig. S15. Biosafety evaluation of DC@CFO/BFO nanoparticles by blood biochemistry tests. (**A-F**) C57BL/6 mice were administrated with 50 mg/kg of the DC@CFO/BFO nanocomposites via intravenous injection and subjected to magnetic field stimulation (1.5 mT) for 30 minutes every

day. After 7 days post injection, mice serum was collected and blood biochemistry test was performed. ALT, Alanine aminotransferase; AST, Aspartate aminotransferase; ALP, Alkaline phosphatase; CREA, Creatinine; TP, Total protein (n = 3, mean ± s.e.m., ns, not significant (P > 0.05), Kruskal-Wallis Test).

(G) C57BL/6 mice were administrated with 50 mg/kg of the DC@CFO/BFO nanocomposites via intravenous injection and subjected to magnetic field stimulation (1.5 mT) for 30 minutes every day. After 7 days post injection, the major organs of the mice were collected and representative H&E (Hematoxylin-eosin) staining images were shown. The scale bars represent 100 μm.

Fig. S16

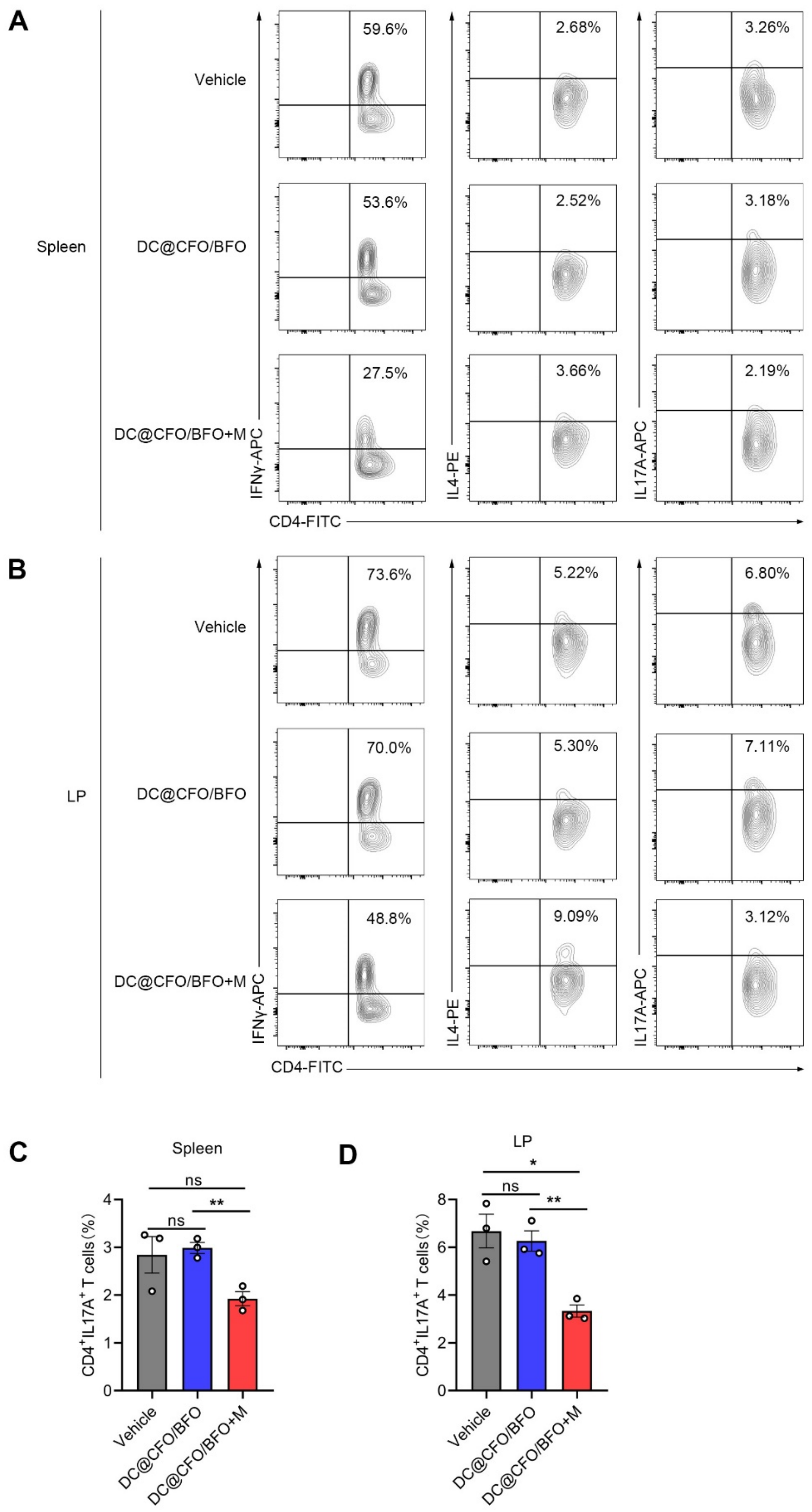

A
Spleen
Vehicle
DC@CFO/BFO
DC@CFO/BFO+M
59.6%
53.6%
27.5%
2.68%
2.52%
3.66%
3.26%
3.18%
2.19%
IFNγ-APC
IL4-PE
IL17A-APC
CD4-FITC
B
LP
Vehicle
DC@CFO/BFO
DC@CFO/BFO+M
73.6%
70.0%
48.8%
5.22%
5.30%
9.09%
6.80%
7.11%
3.12%
IFNγ-APC
IL4-PE
IL17A-APC
CD4-FITC
C
Spleen
ns
**
ns
CD4+IL17A+ T cells (%)
Vehicle
DC@CFO/BFO
DC@CFO/BFO+M
D
LP
*
ns
**
CD4+IL17A+ T cells (%)
Vehicle
DC@CFO/BFO
DC@CFO/BFO+M

Fig. S16. Flow cytometric analysis of $CD4^+$ T subsets in colitis mice.

(A) Flow cytometric analysis of the proportion of $CD4^+$ T cells in mice spleen from the different treatment groups.

(B) Flow cytometric analysis of the proportion of $CD4^+$ T cells in mice lamina propria (LP) from the different treatment groups.

(C, D) Flow cytometric analysis of the proportion of $CD4^+IL17A^+$ cells in mice spleen and LP from the different treatment groups (n = 3, mean ± s.e.m., ns, not significant ($P > 0.05$), *$P = 0.0110$, **$P < 0.01$, (Shapiro-Wilk test $P > 0.1$, F test $P > 0.1$) two-tailed unpaired Student's t-test).

Fig. S17

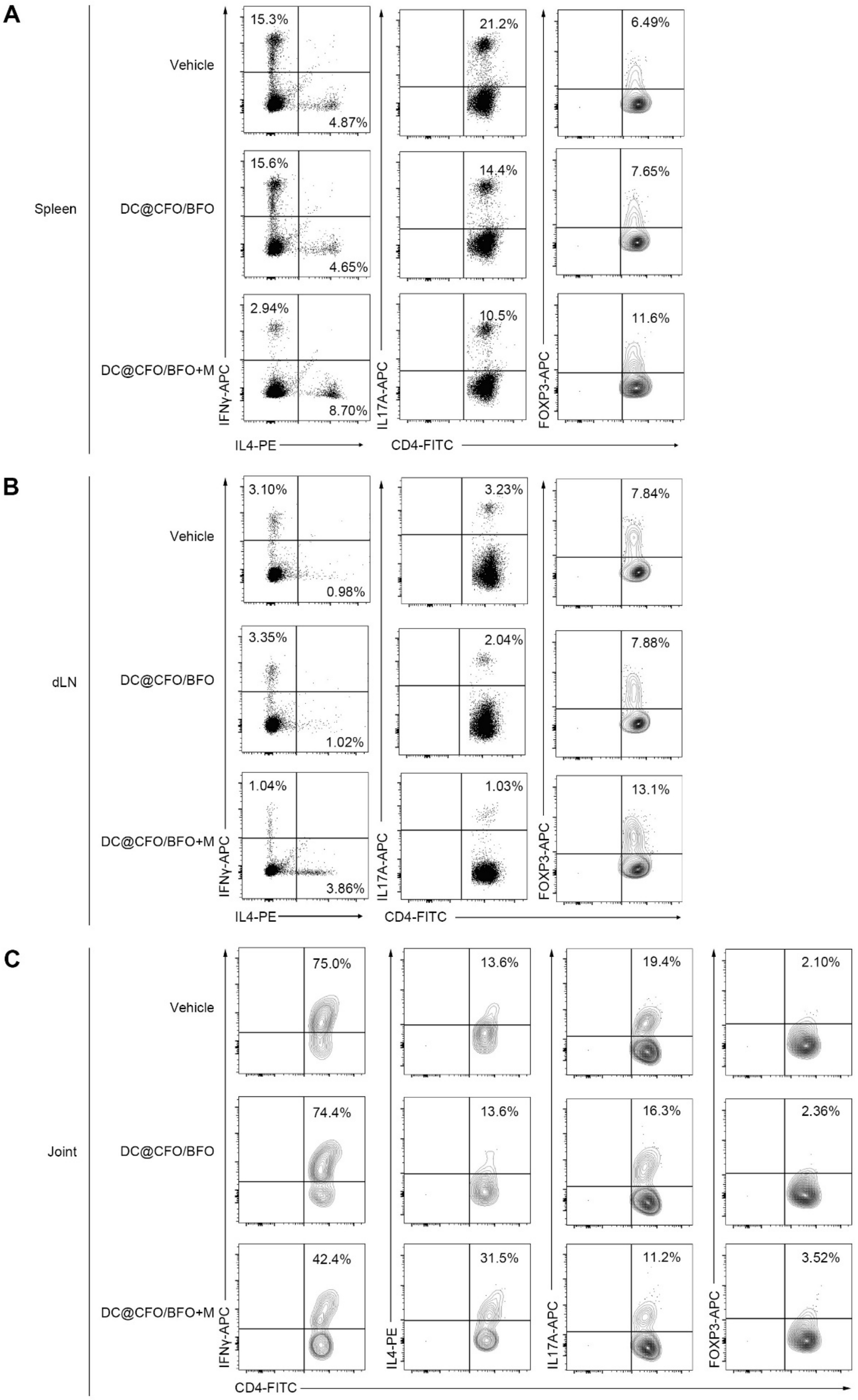
A
Spleen
Vehicle
DC@CFO/BFO
DC@CFO/BFO+M
15.3%
4.87%
21.2%
6.49%
15.6%
4.65%
14.4%
7.65%
2.94%
8.70%
10.5%
11.6%
IFNγ-APC
IL17A-APC
FOXP3-APC
IL4-PE
CD4-FITC
B
dLN
Vehicle
DC@CFO/BFO
DC@CFO/BFO+M
3.10%
0.98%
3.23%
7.84%
3.35%
1.02%
2.04%
7.88%
1.04%
3.86%
1.03%
13.1%
IFNγ-APC
IL17A-APC
FOXP3-APC
IL4-PE
CD4-FITC
C
Joint
Vehicle
DC@CFO/BFO
DC@CFO/BFO+M
75.0%
13.6%
19.4%
2.10%
74.4%
13.6%
16.3%
2.36%
42.4%
31.5%
11.2%
3.52%
IFNγ-APC
IL4-PE
IL17A-APC
FOXP3-APC
CD4-FITC

Fig. S17. Flow cytometric analysis of $CD4^+$ T cell subsets in arthritic mice.

(A) Flow cytometric analysis of the proportion of $CD4^+$ T cells in mice spleen from the different treatment groups.

(B) Flow cytometric analysis of the proportion of $CD4^+$ T cells in mice draining LN (dLN) from the different treatment groups.

(C) Flow cytometric analysis of the proportion of $CD4^+$ T cells in mice ankle joints from the different treatment groups.

Fig. S18

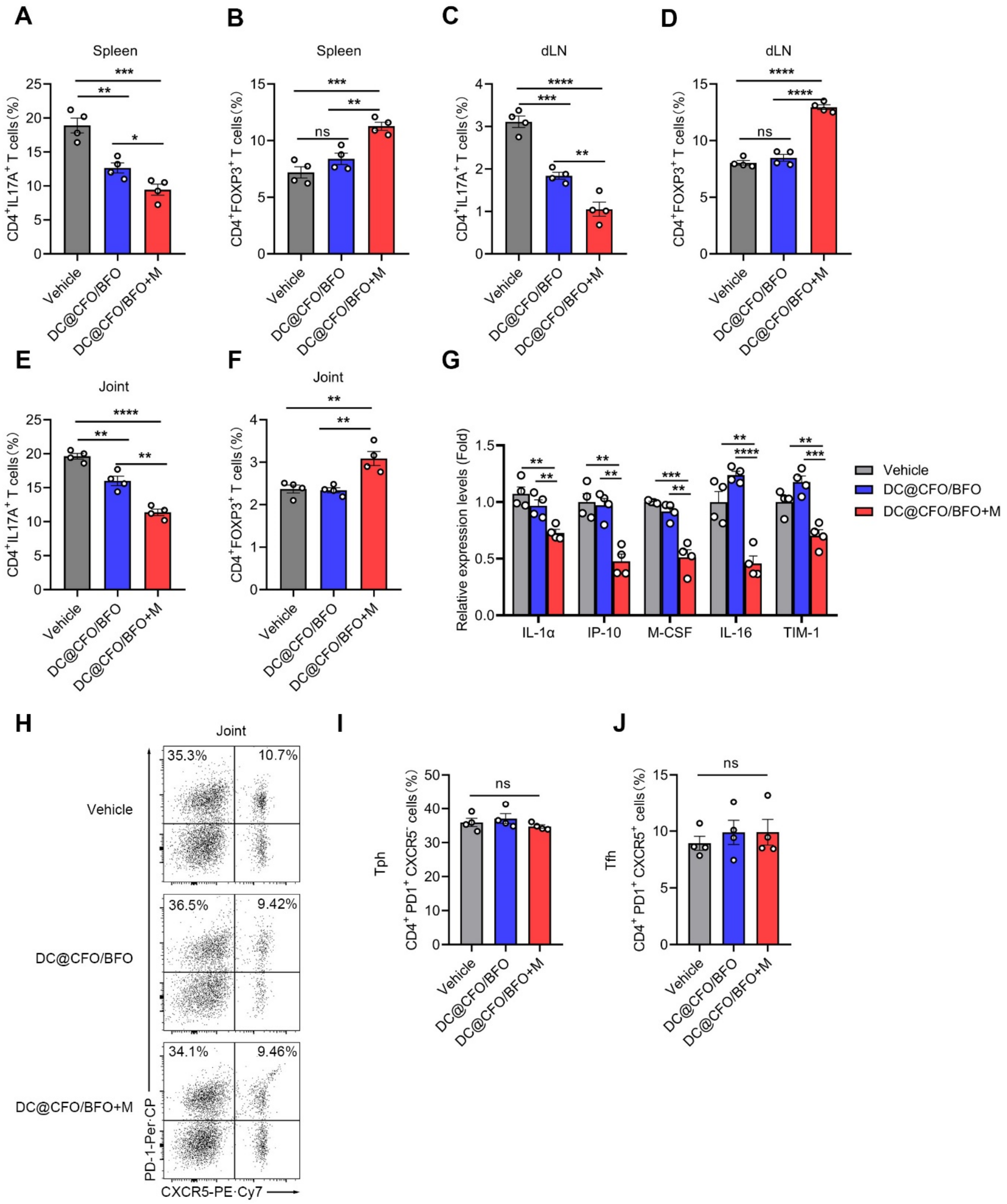

Fig. S18. T cell subset analysis and serum inflammation evaluation of arthritic mice.

(A, B) Flow cytometric analysis of the proportion of $CD4^{+}IL17A^{+}$ cells and $CD4^{+}FOXP3^{+}$ cells in mice spleen from the different treatment groups (n = 4, mean ± s.e.m., $**P < 0.01$, $***P < 0.001$ (Shapiro-Wilk test $P > 0.1$, F test $P > 0.1$) two-tailed unpaired Student's t-test).

(C, D) Flow cytometric analysis of the proportion of $CD4^{+}IL17A^{+}$ cells and $CD4^{+}FOXP3^{+}$ cells in mice draining LN (dLN) from the different treatment groups (n = 4, mean ± s.e.m., $**P = 0.0053$, $***P = 0.0002$, $***P < 0.0001$, (Shapiro-Wilk test $P > 0.1$, F test $P > 0.1$) two-tailed unpaired Student's t-test).

(E, F) Flow cytometric analysis of the proportion of $CD4^{+}IL17A^{+}$ cells and $CD4^{+}FOXP3^{+}$ cells in mice ankle joints from the different treatment groups (n = 4, mean ± s.e.m., $**P < 0.01$, $****P < 0.0001$, (Shapiro-Wilk test $P > 0.1$, F test $P > 0.1$) two-tailed unpaired Student's t-test).

(G) Mice serum was obtained and cytokine array was performed to evaluate indicated inflammatory factors (n = 4, mean ± s.e.m., $**P < 0.01$, $***P < 0.001$, $****P < 0.0001$, (Shapiro-Wilk test $P > 0.1$, F test $P > 0.1$) two-tailed unpaired Student's t-test).

(H-J) Flow cytometric analysis of the proportion of $CD4^{+}PD\text{-}1^{+}CXCR5^{-}$ and $CD4^{+}PD\text{-}1^{+}CXCR5^{+}$ cells in mice ankle joints from the different treatment groups (n = 4, mean ± s.e.m., ns, not significant ($P > 0.05$), (Shapiro-Wilk test $P > 0.1$, F test $P > 0.1$) two-tailed unpaired Student's t-test).

Fig. S19

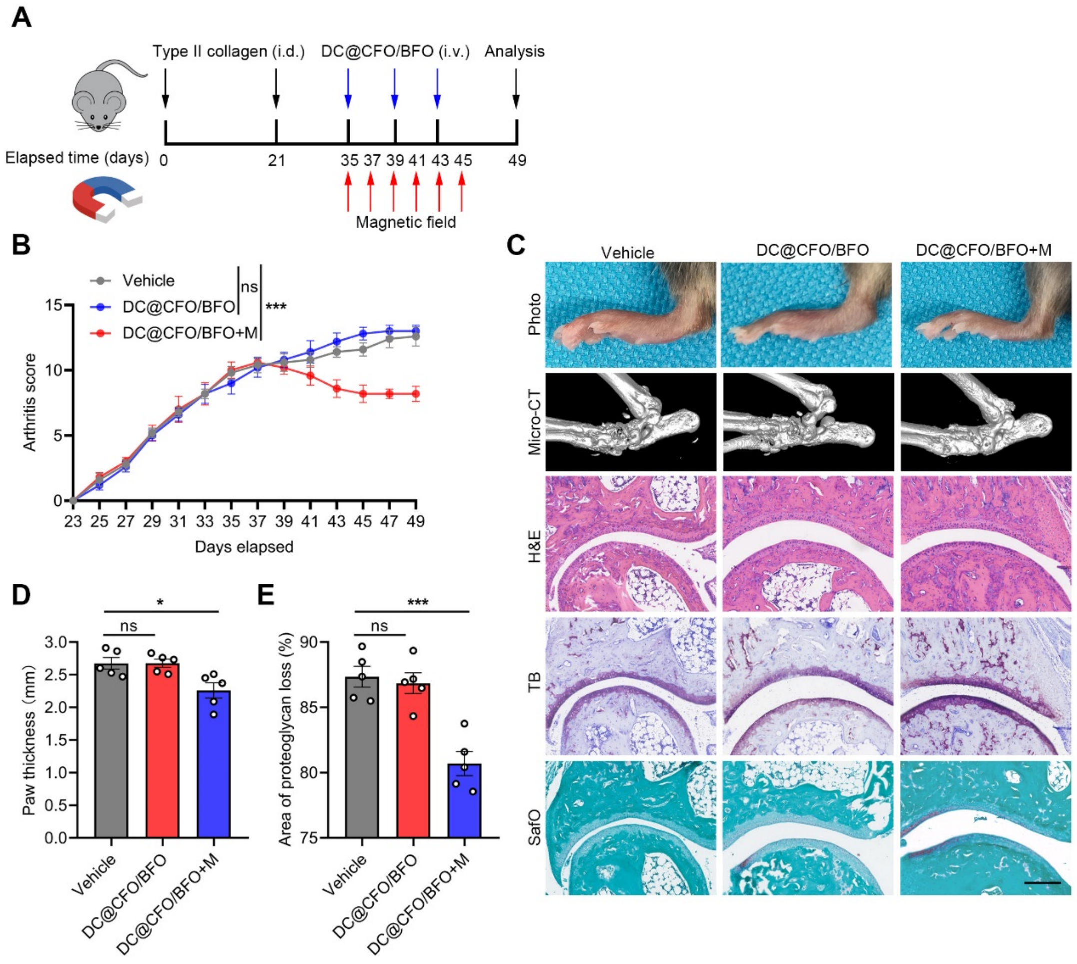

Fig. S19. Magnetoelectric nanoparticles ameliorate collagen-induced arthritis under external magnetic field stimulation.

(A) Graphic illustration of the study design of DC@CFO/BFO nanoparticles treatment in a CIA mouse model. Briefly, DBA/1 mice were injected intradermally with bovine type II collagen (CII) emulsified in Freund's complete adjuvant or Freund's incomplete adjuvant at day 0 and day 21. 10 mg/kg DC@CFO/BFO nanoparticles were intravenously injected into mice and 1.5 mT magnetic field was applied for 30 minutes after nanoparticle injection at indicated timepoints.

(B) Arthritis score of arthritic animals were assessed every other day after disease onset. The clinical scores were assigned to evaluate disease as follows: 0 = no signs of arthritis; 1 = swelling

and/or redness of the paw or one digit; 2 = two joints involved; 3 = more than two joints involved and 4 = severe arthritis of the entire paw and digits. Each limb was graded, resulting in a maximal clinical score of 16 per animal (n = 5, mean ± s.e.m., ***$P < 0.001$, (Shapiro-Wilk test, $P > 0.1$; Brown-Forsythe test, $P > 0.1$) one-way ANOVA).

(C) Representative images including gross photographs, micro-CT images, H&E staining images, toluidine blue (TB) staining images and safranin-O/ fast green (SafO) staining images of ankle joints from different treatment groups were shown. The scale bars represent 200 μm.

(D) The paw thickness of arthritic mice from different treatment groups were measured (n = 5, mean ± s.e.m., ns, not significant ($P > 0.05$), *$P < 0.05$, (Shapiro-Wilk test, $P > 0.1$; Brown-Forsythe test, $P > 0.1$) one-way ANOVA).

(E) Proteoglycan loss of ankle joints was measured by Image J Microsoft according to safranin-O/ fast green staining images (n = 5, mean ± s.e.m., ns, not significant ($P > 0.05$), ***$P < 0.001$, (Shapiro-Wilk test, $P > 0.1$; Brown-Forsythe test, $P > 0.1$) one-way ANOVA).

Fig. S20

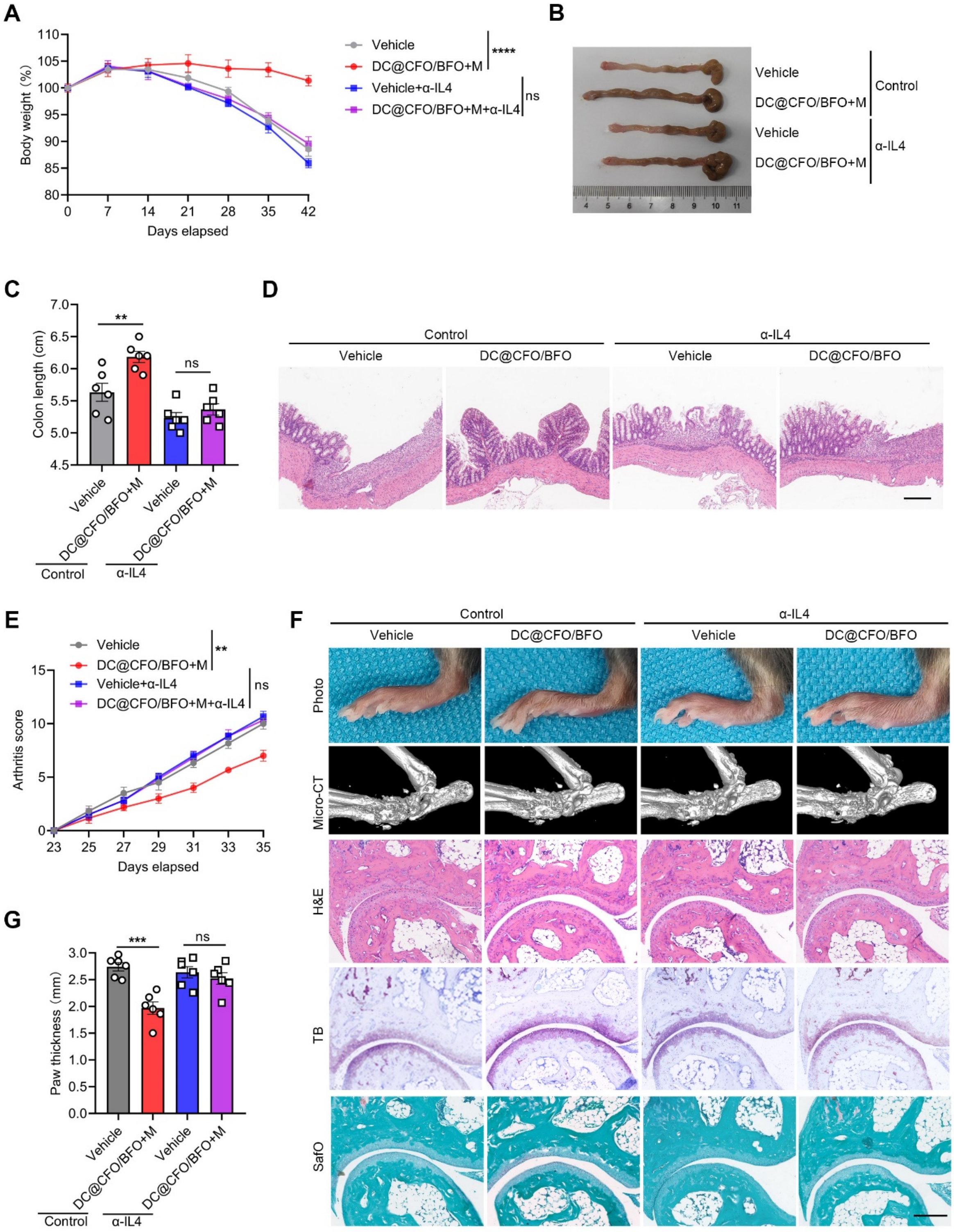

Fig. S20. Th2 cells are critical for therapeutic role of magnetoelectric nanoparticles.

(A) Body weights of mice from different treatment groups were assessed every week (n = 6, mean ± s.e.m., ns, not significant ($P > 0.05$), ****$P < 0.0001$, (Shapiro-Wilk test, $P > 0.1$; Brown-Forsythe test, $P > 0.1$) one-way ANOVA).

(B, C) Macroscopic evaluation (**B**) and colon length (**C**) of mice from different treatment groups on day 42 (n = 6, mean ± s.e.m., ns, not significant ($P > 0.05$), **$P < 0.01$, (Shapiro-Wilk test, $P > 0.1$; Brown-Forsythe test, $P > 0.1$) one-way ANOVA).

(D) Representative H&E staining images of colon tissues from mice of different treatment groups on day 42. The scale bars represent 200 μm.

(E) Arthritis score of arthritic animals were assessed every other day after disease onset. The clinical scores were assigned to evaluate arthritic disease as follows: 0 = no signs of arthritis; 1 = swelling and/or redness of the paw or one digit; 2 = two joints involved; 3 = more than two joints involved and 4 = severe arthritis of the entire paw and digits. Each limb was graded, resulting in a maximal clinical score of 16 per animals (n = 6, mean ± s.e.m., ns, not significant ($P > 0.05$), **$P < 0.01$, (Shapiro-Wilk test, $P > 0.1$; Brown-Forsythe test, $P > 0.1$) one-way ANOVA).

(F) Representative images including gross photographs, micro-CT images, H&E staining pictures, toluidine blue (TB) staining images and safranin-O/ fast green (SafO) staining images of ankle joints from different treatment groups were shown. The scale bars represent 200 μm.

(G) Paw thickness of arthritic mice from different treatment groups were measured (n = 6, mean ± s.e.m., ns, not significant ($P > 0.05$), ***$P < 0.001$, (Shapiro-Wilk test, $P > 0.1$; Brown-Forsythe test, $P > 0.1$) one-way ANOVA).

Fig. S21

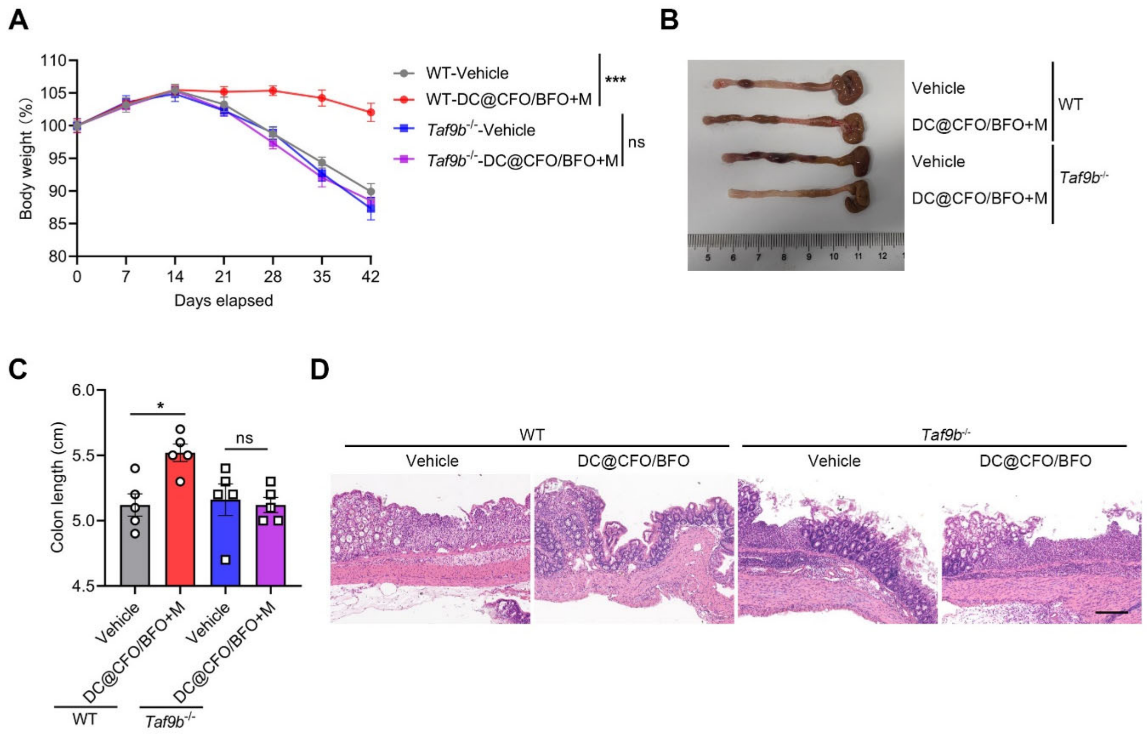

Fig. S21. TAF9B is critical for magnetoelectric nanoparticle-mediated T cell response.

(A) Body weights of mice from different treatment groups were assessed every week (n = 5, mean ± s.e.m., ns, not significant ($P > 0.05$), ***$P < 0.001$, (Shapiro-Wilk test, $P > 0.1$; Brown-Forsythe test, $P > 0.1$) one-way ANOVA).

(B, C) Macroscopic evaluation (**B**) and colon length (**C**) of mice from different treatment groups on day 42 (n = 5, mean ± s.e.m., ns, not significant ($P > 0.05$), *$P < 0.05$, (Shapiro-Wilk test, $P > 0.1$; Brown-Forsythe test, $P > 0.1$) one-way ANOVA).

(D) Representative H&E staining images of colon tissues from mice of different treatment groups on day 42. The scale bars represent 200 μm.
